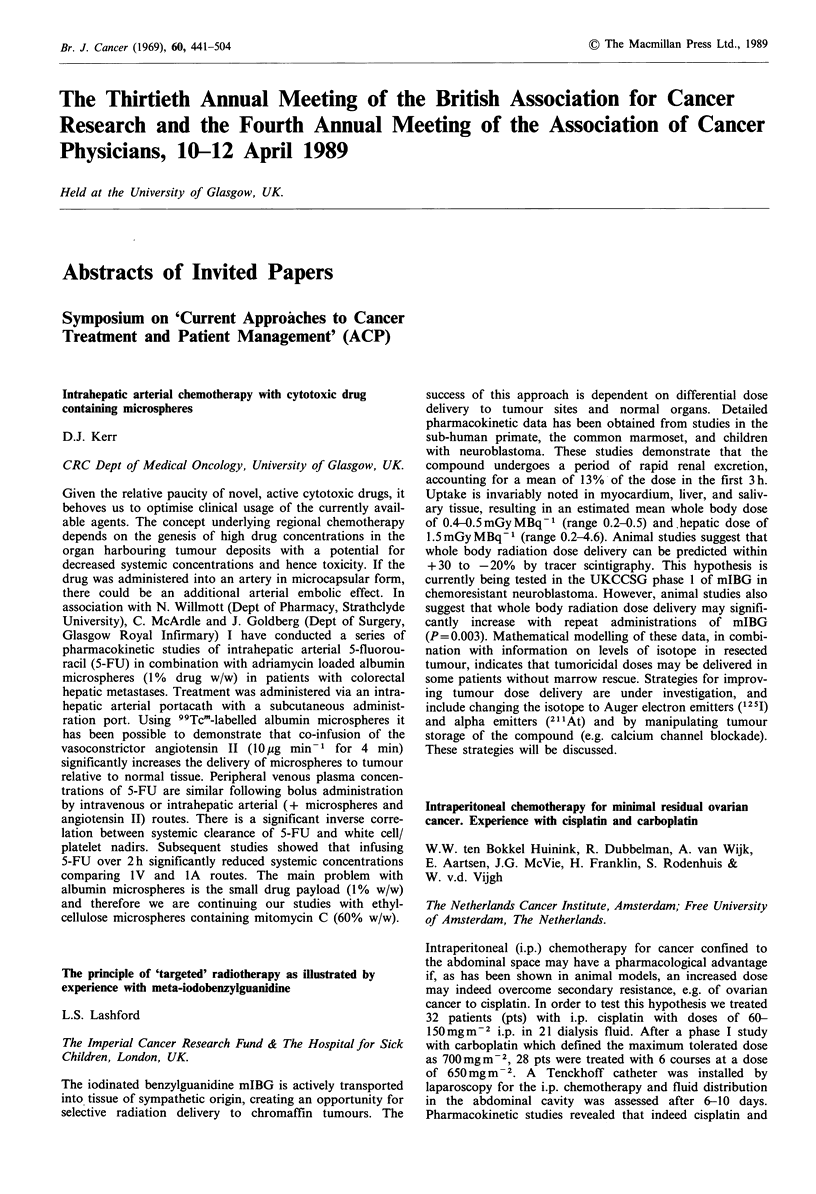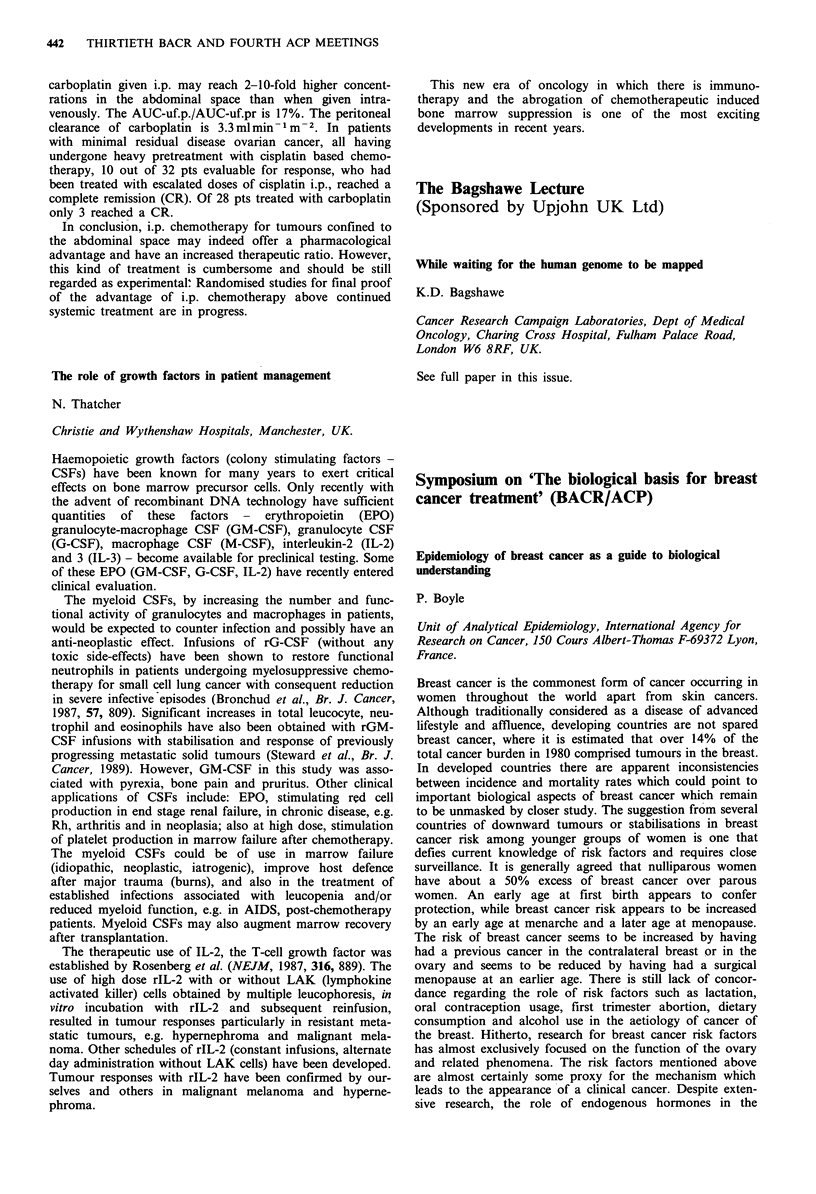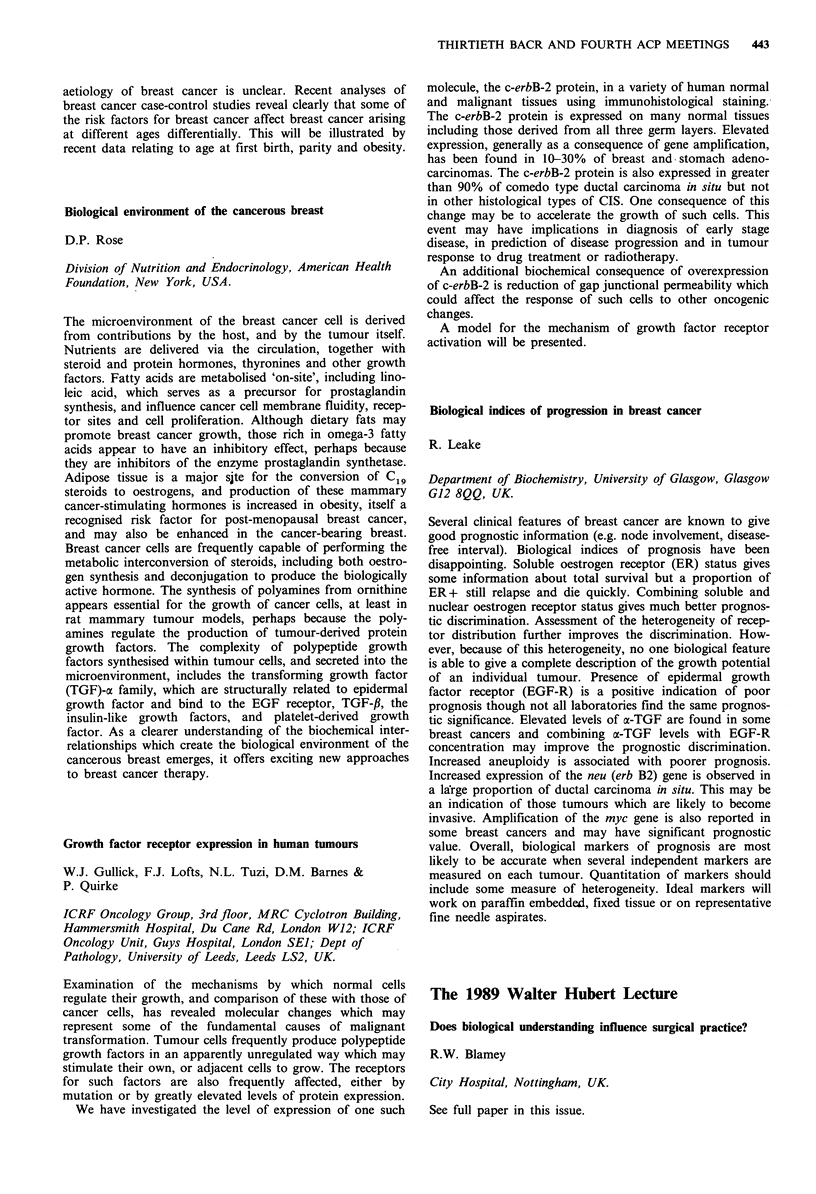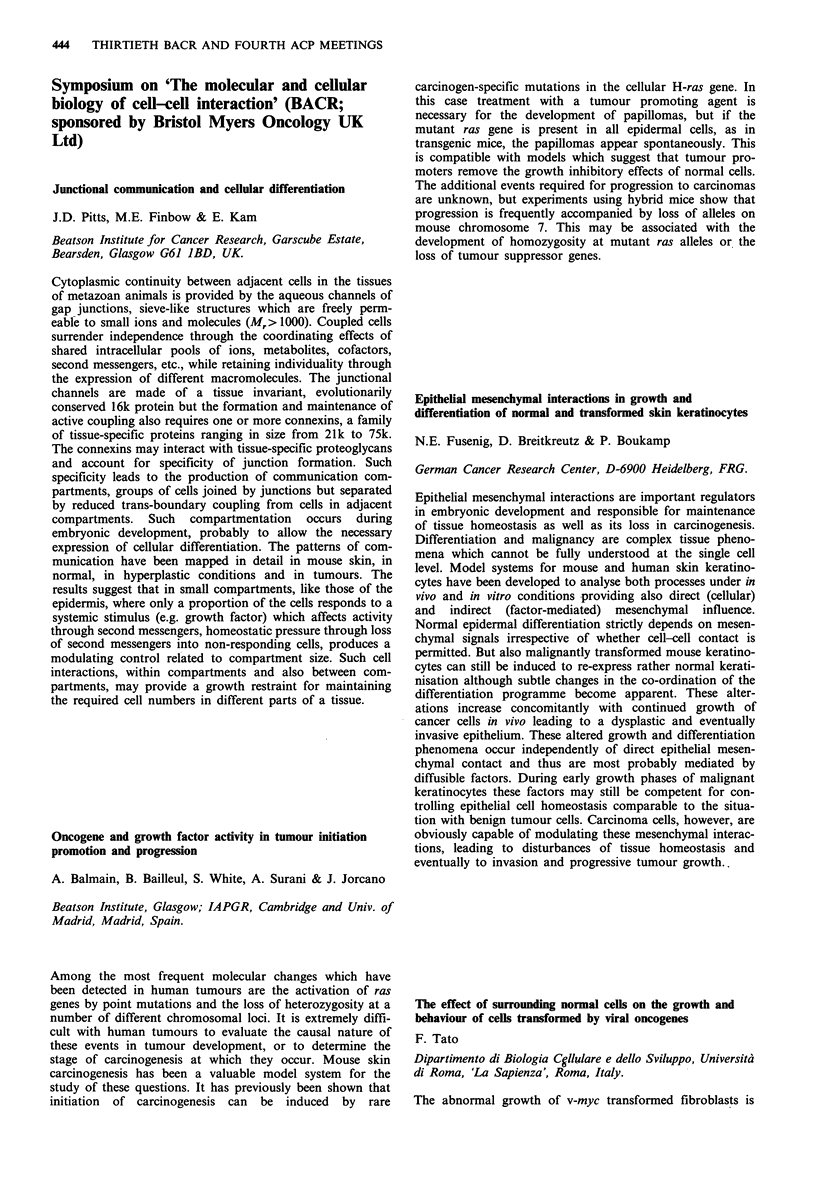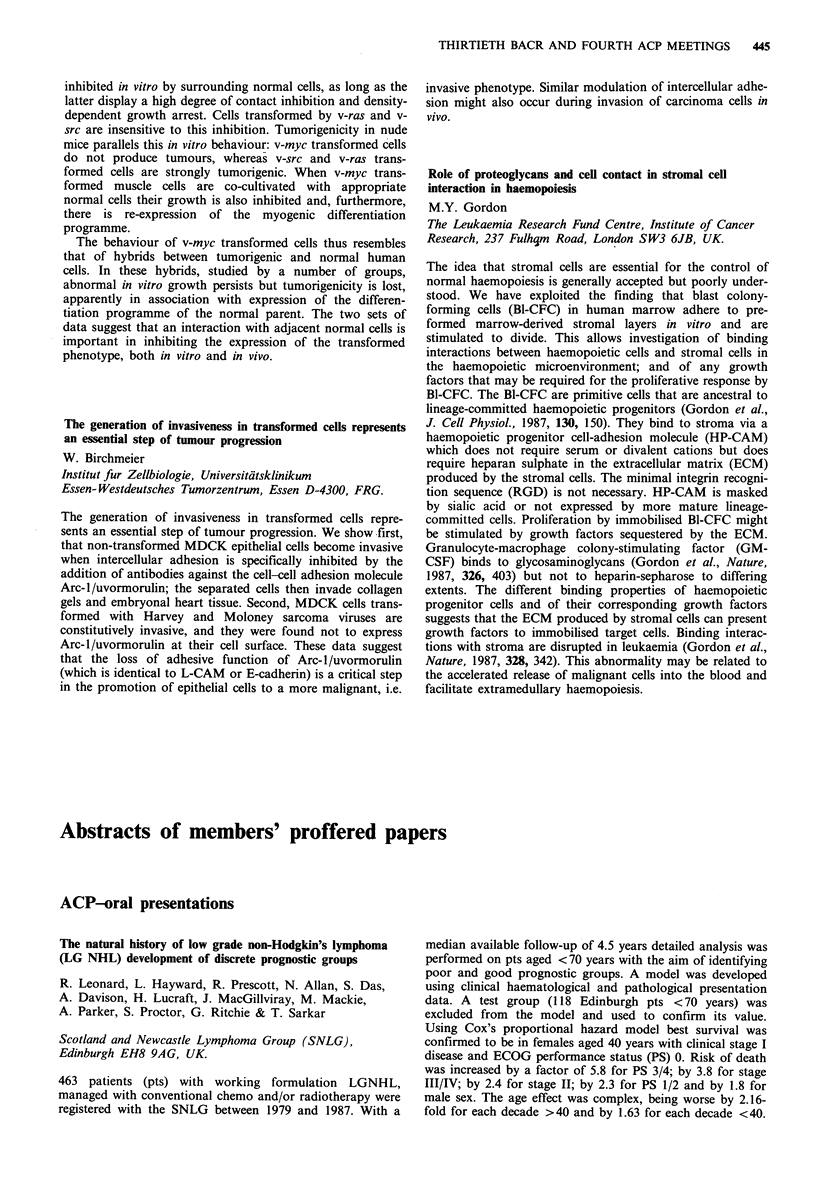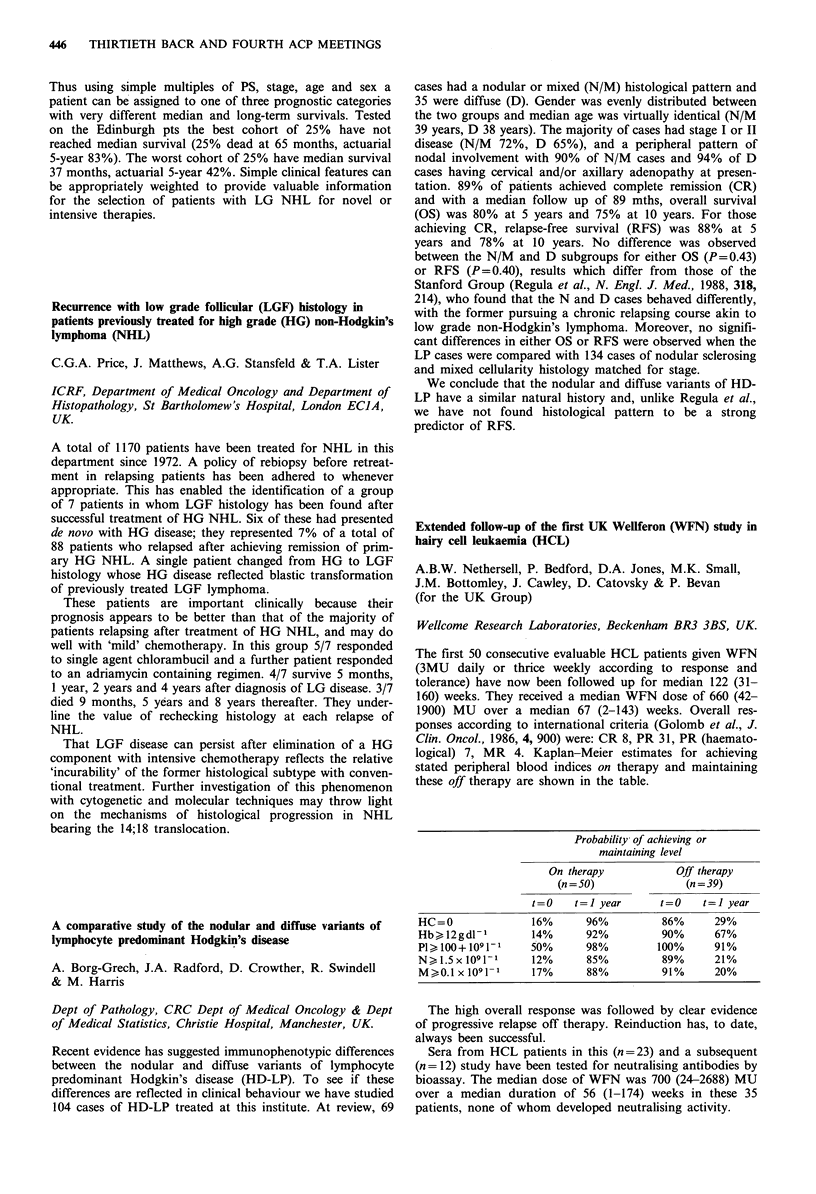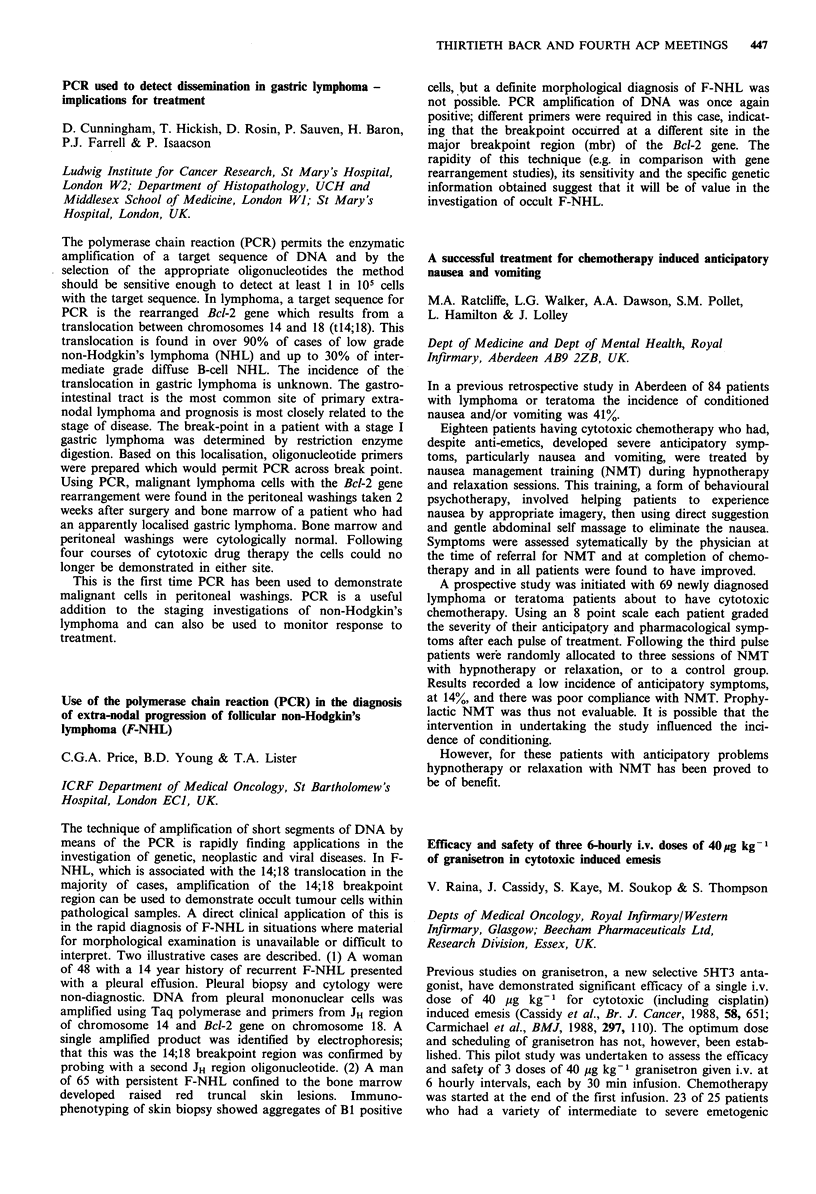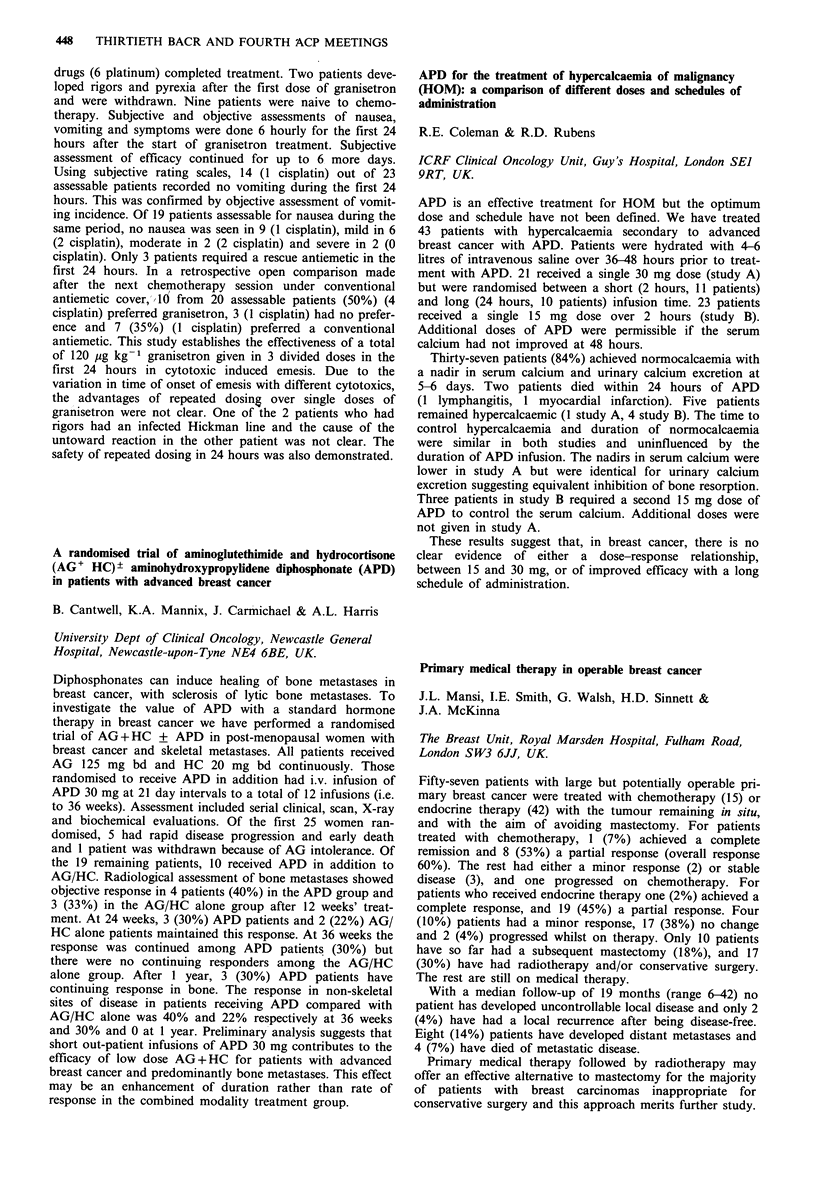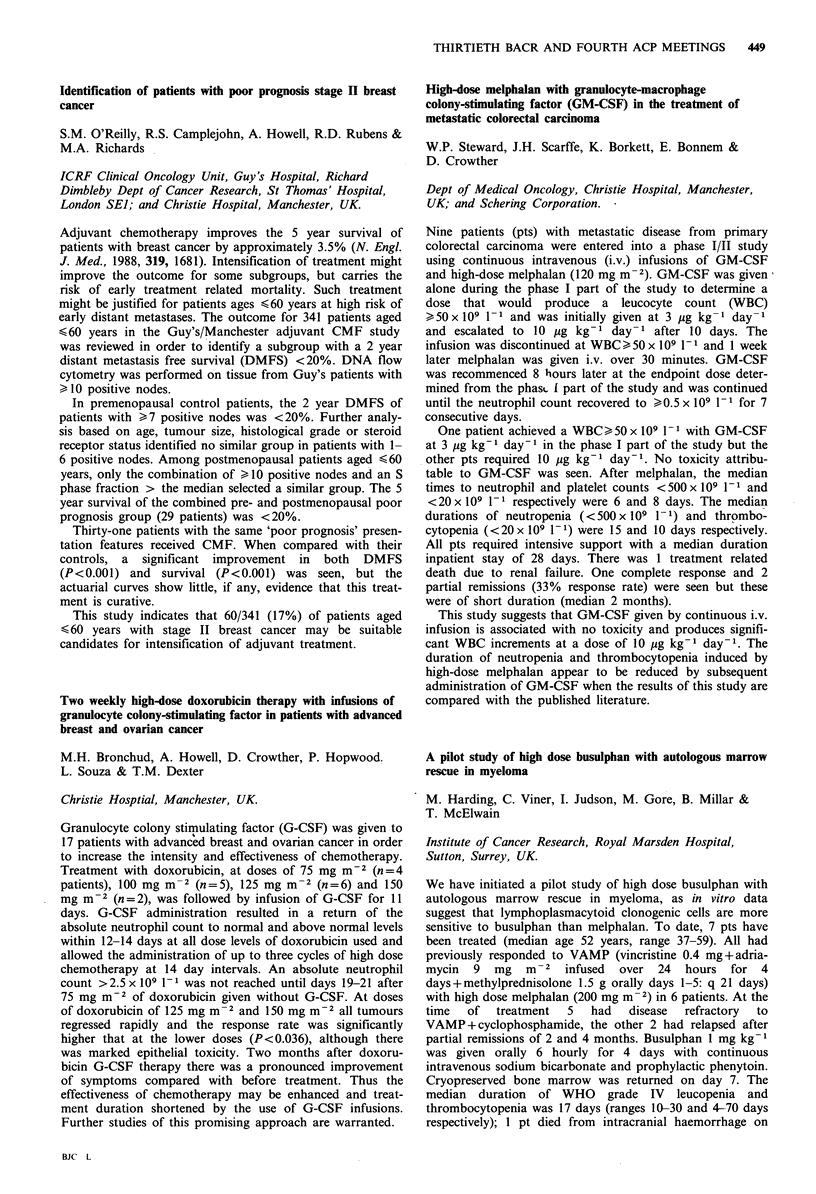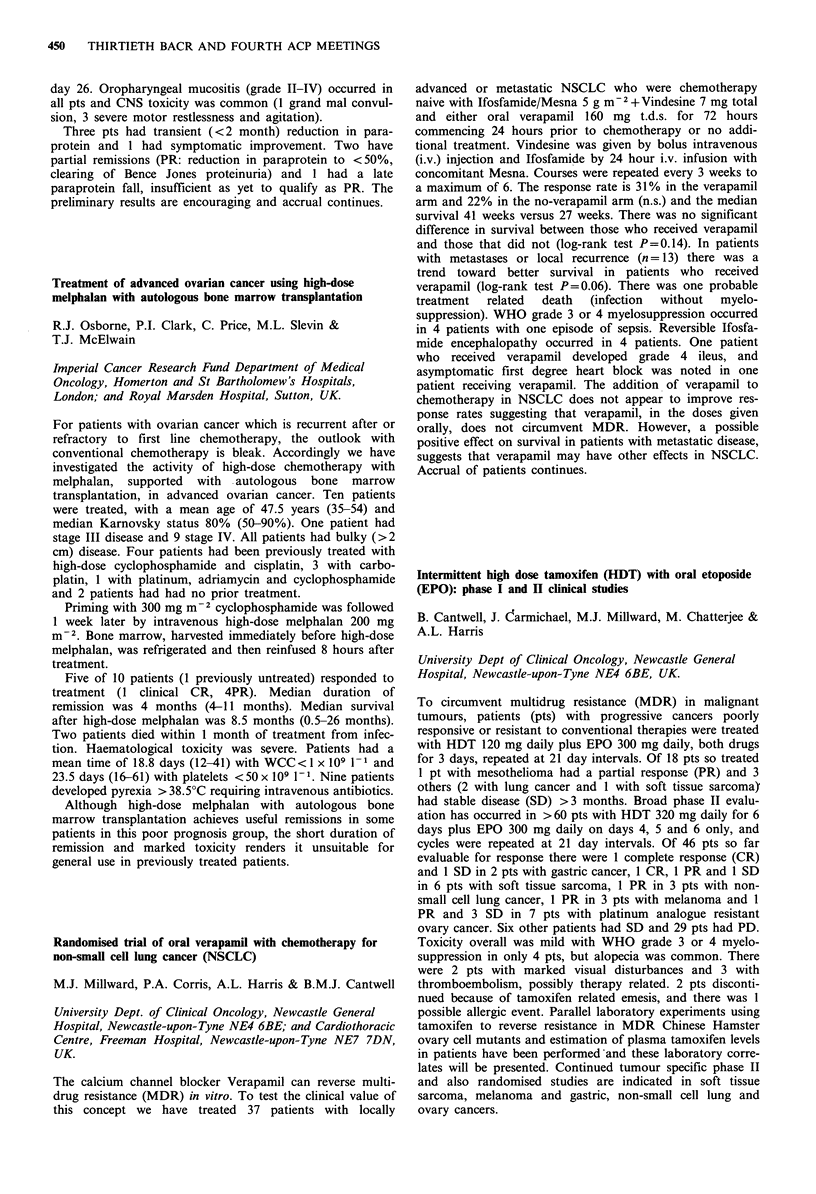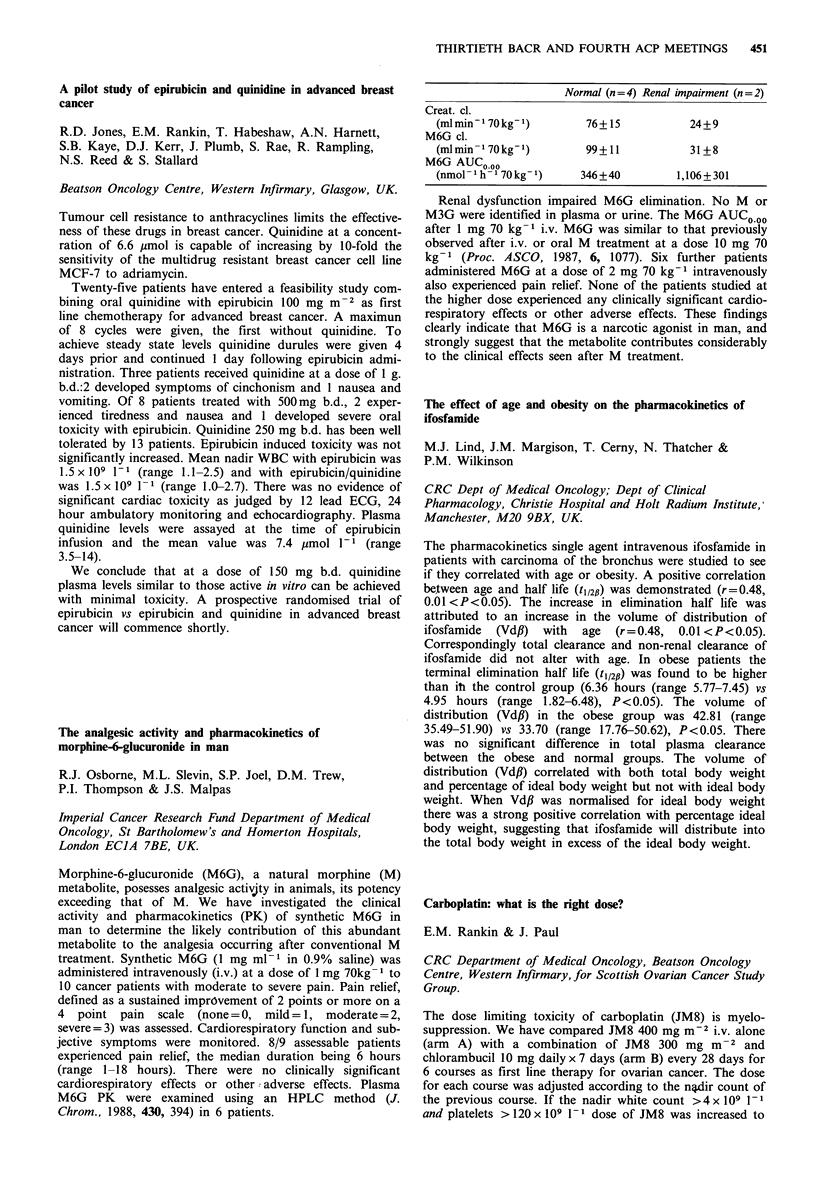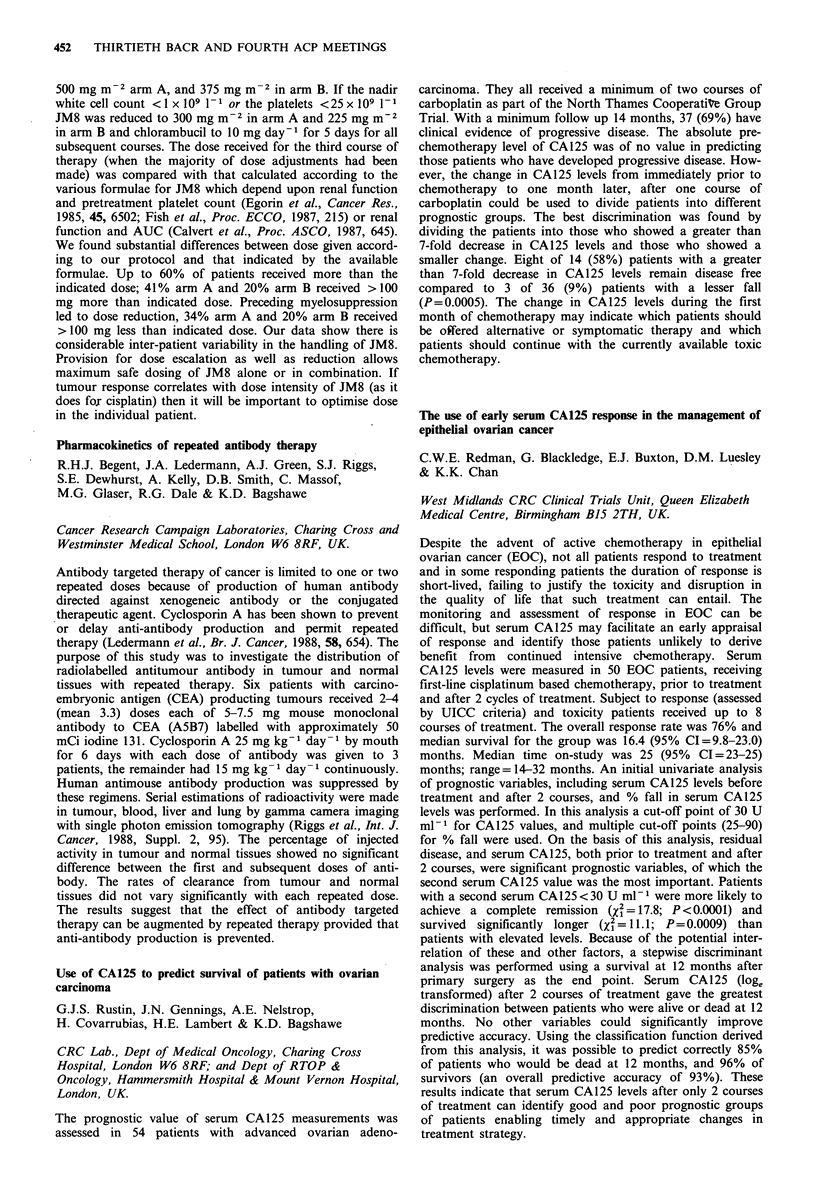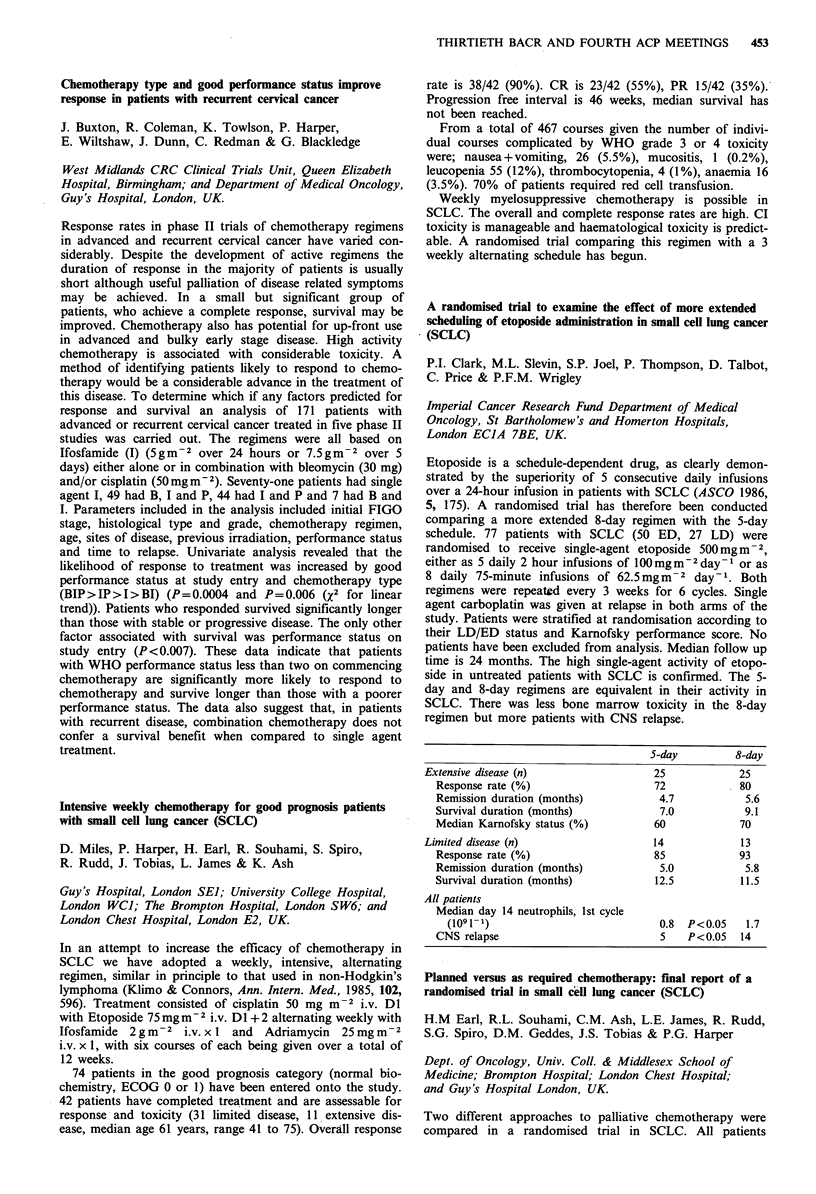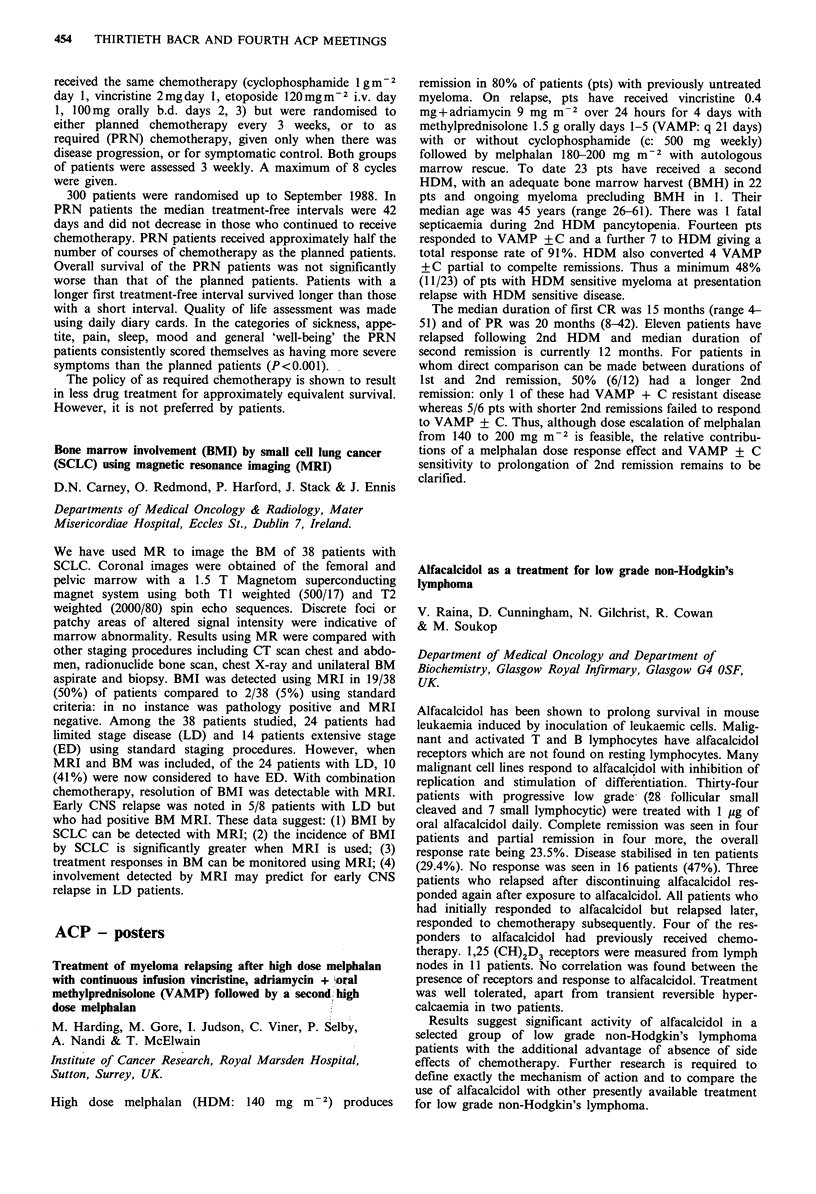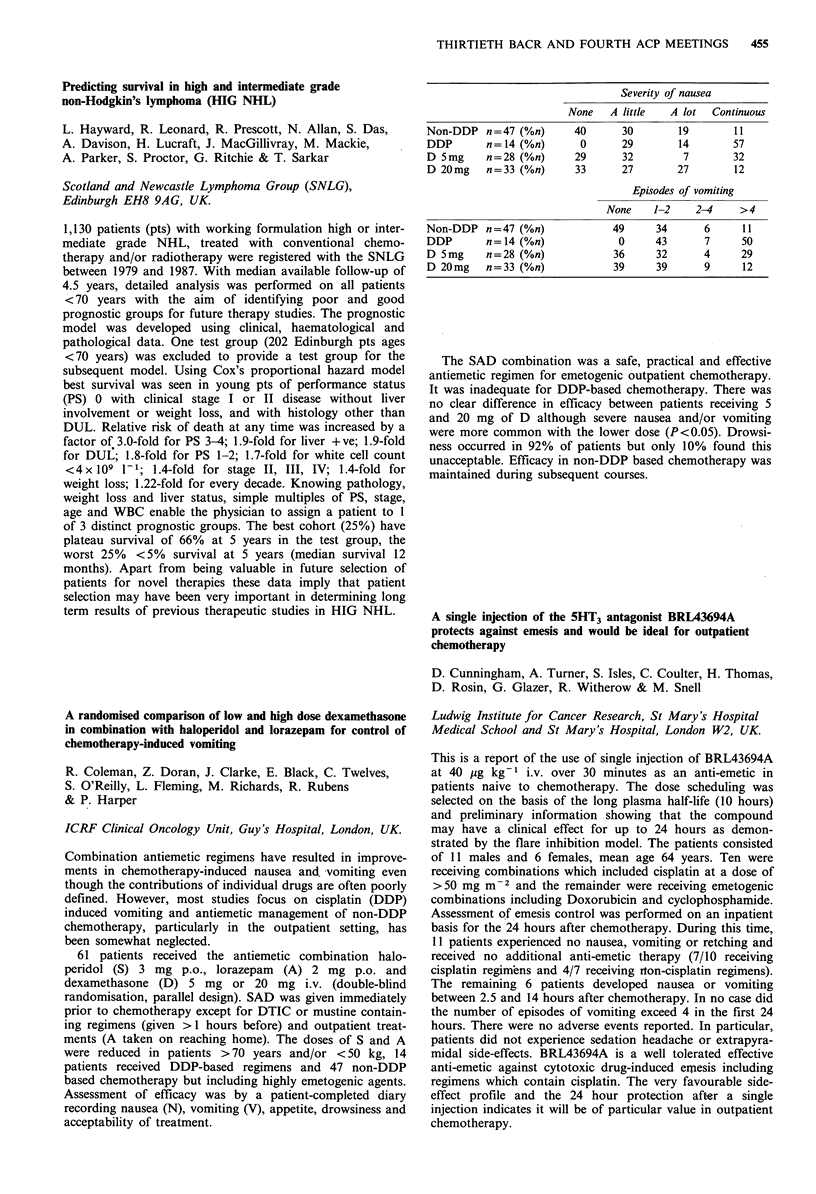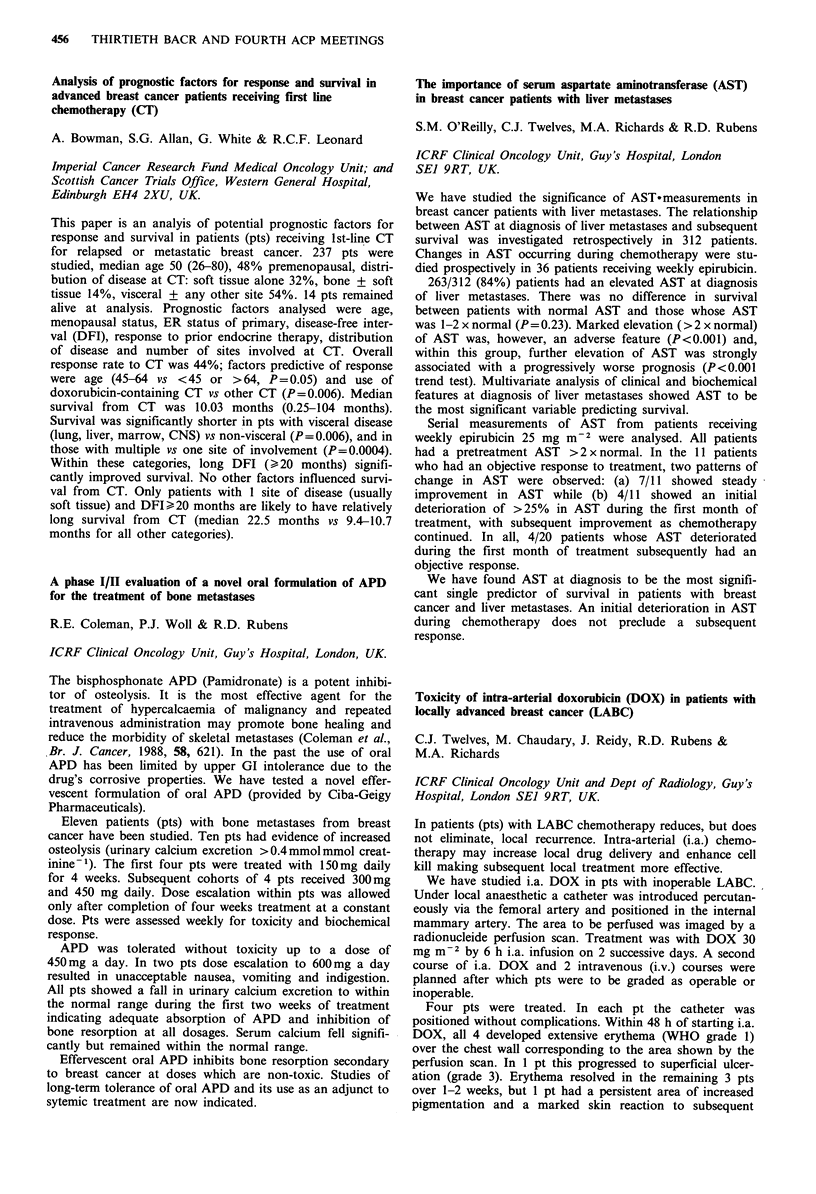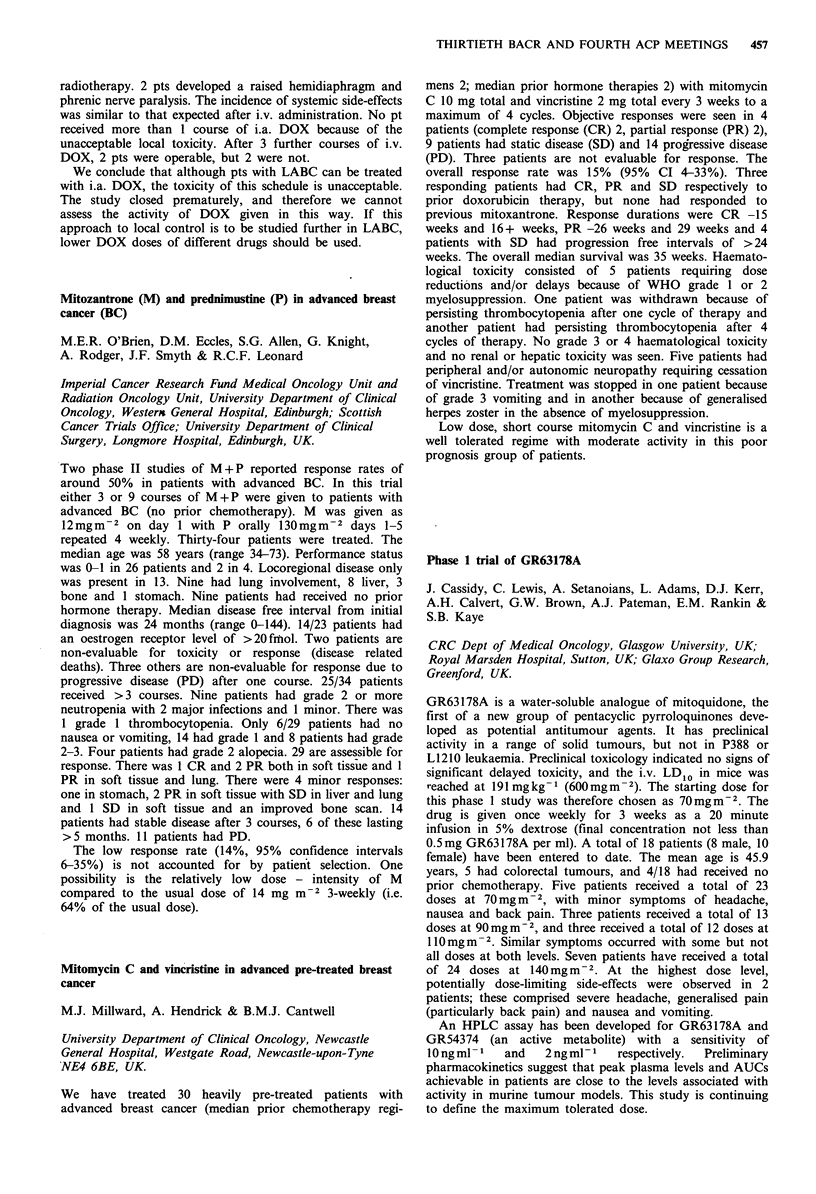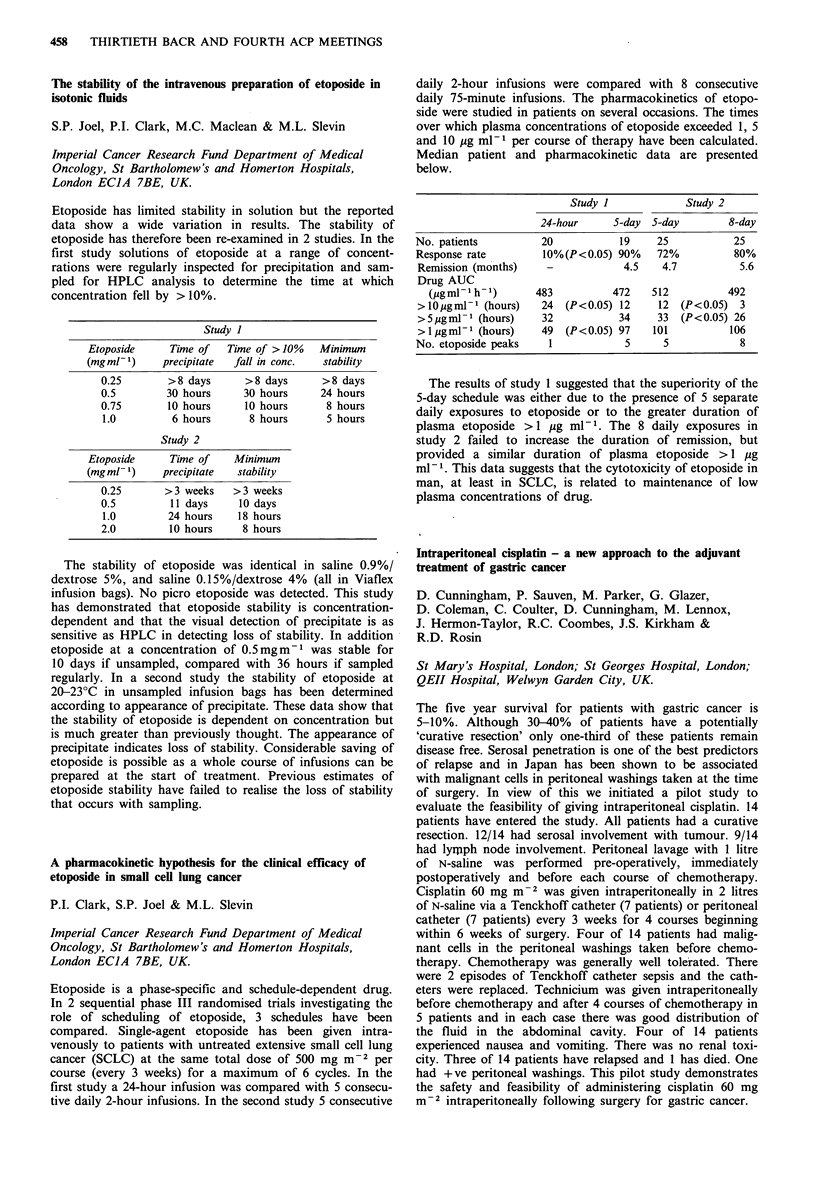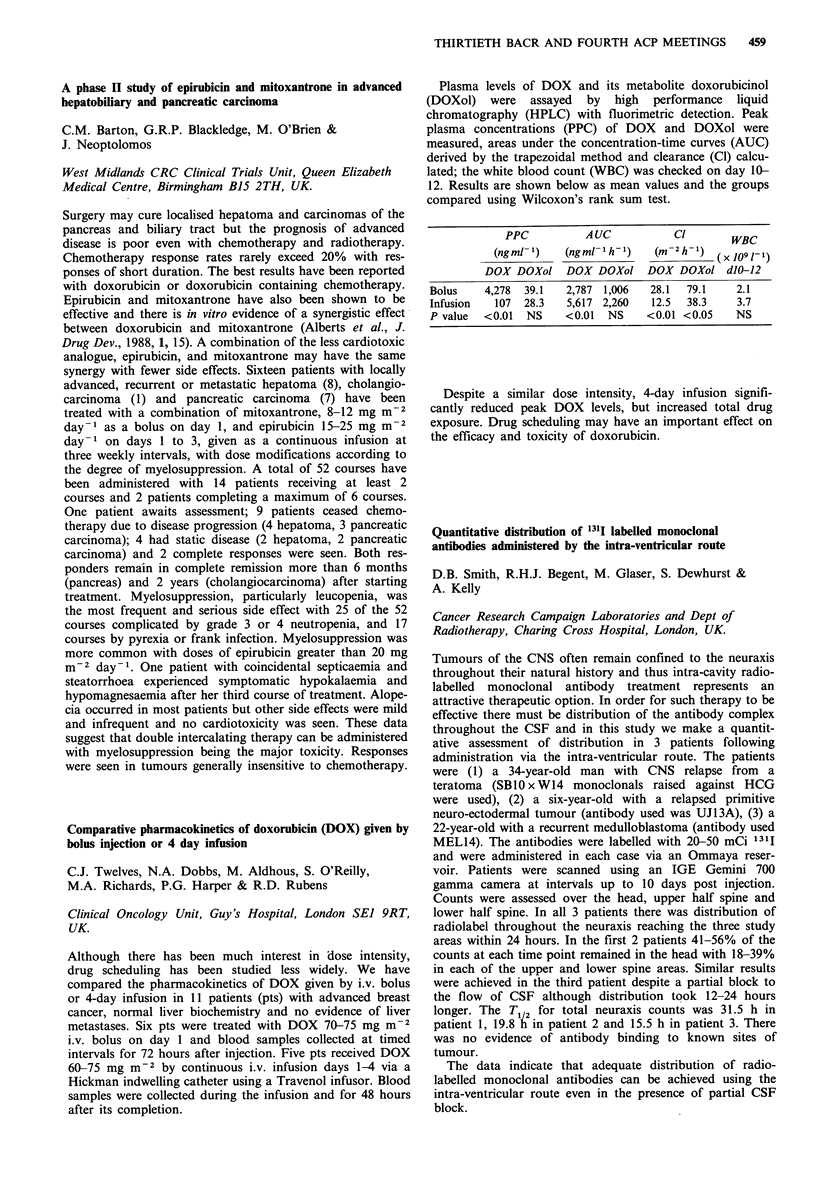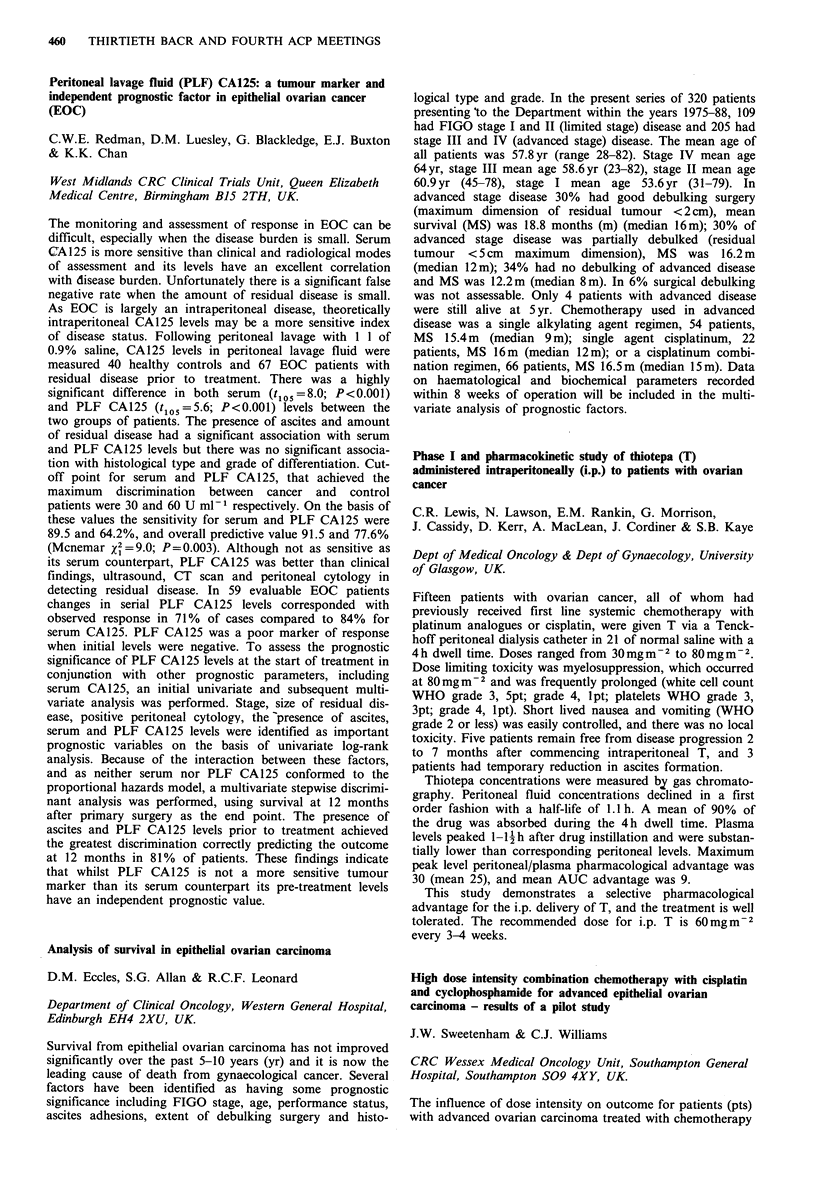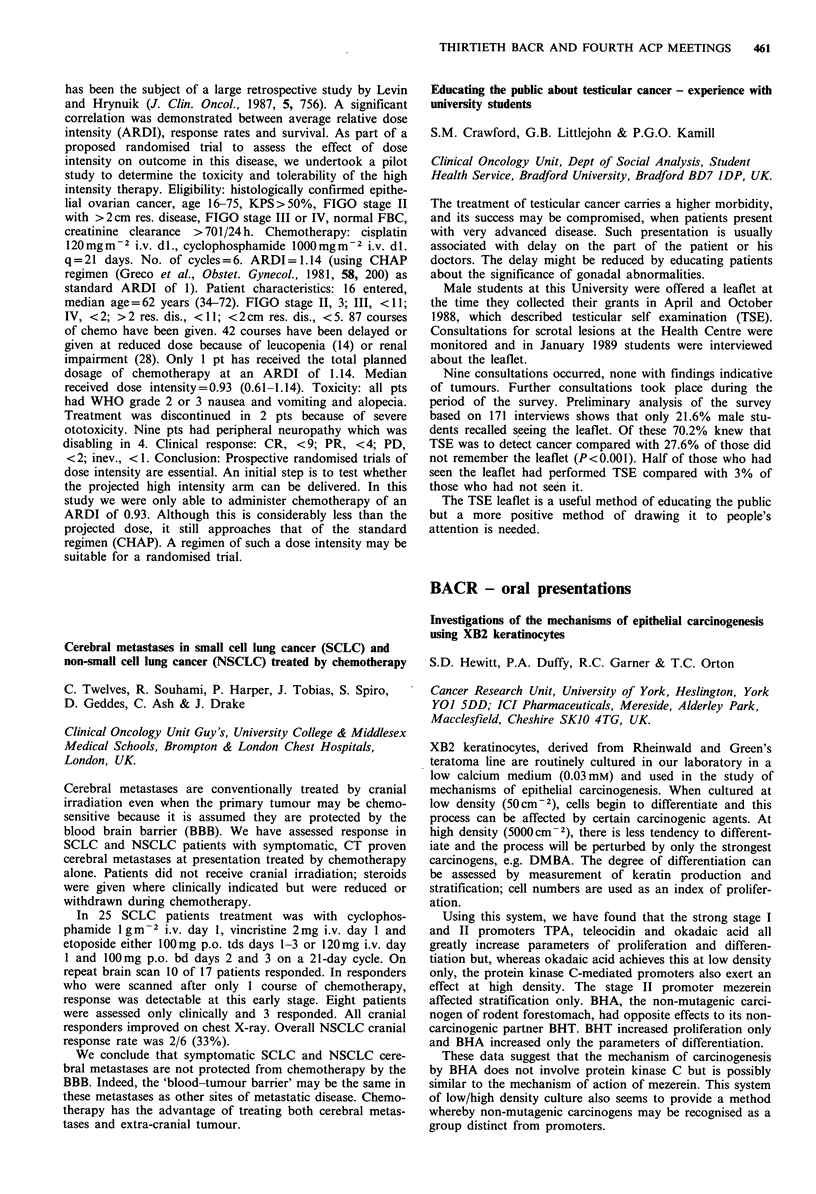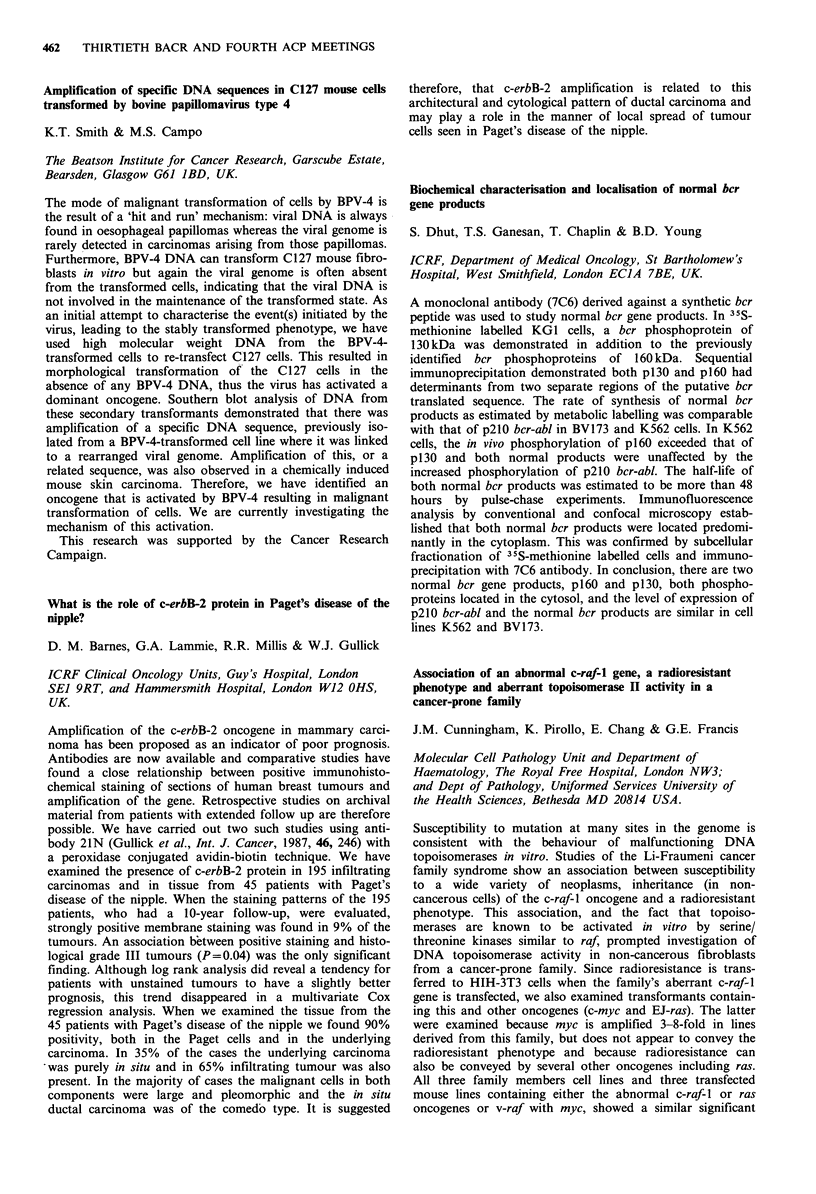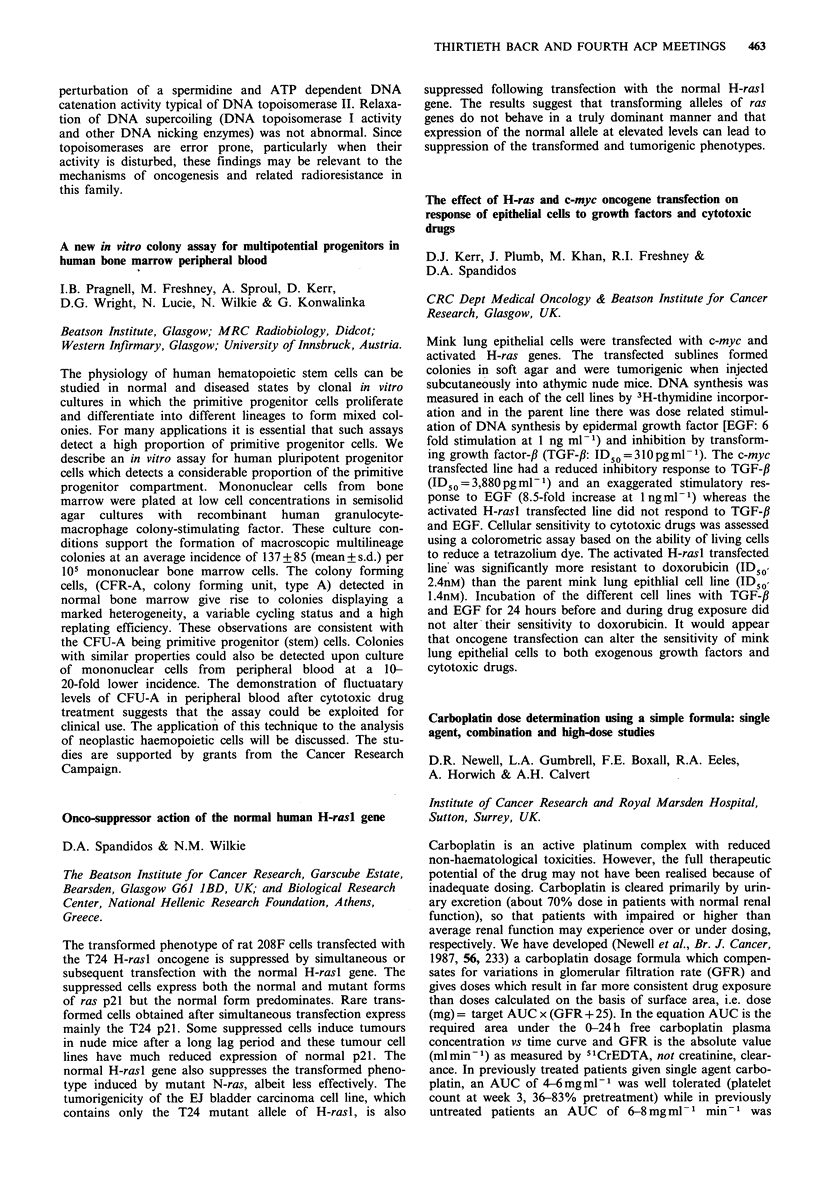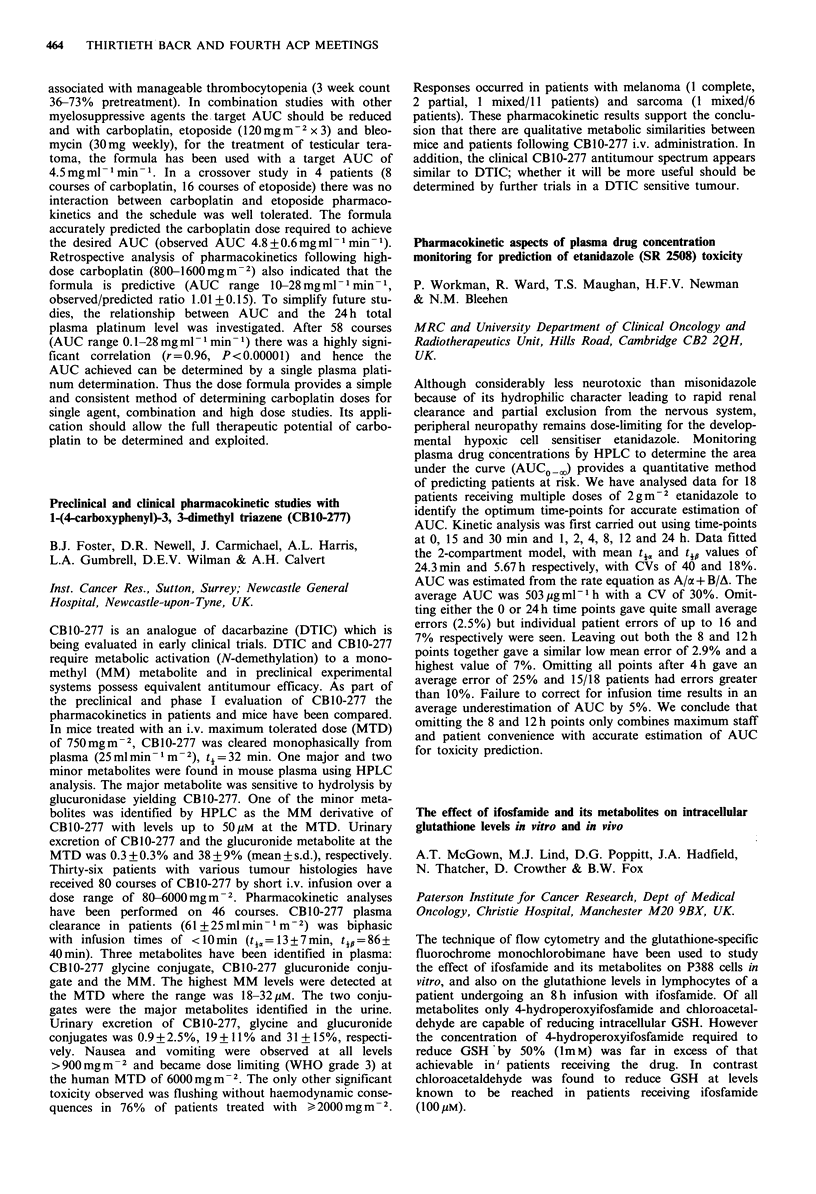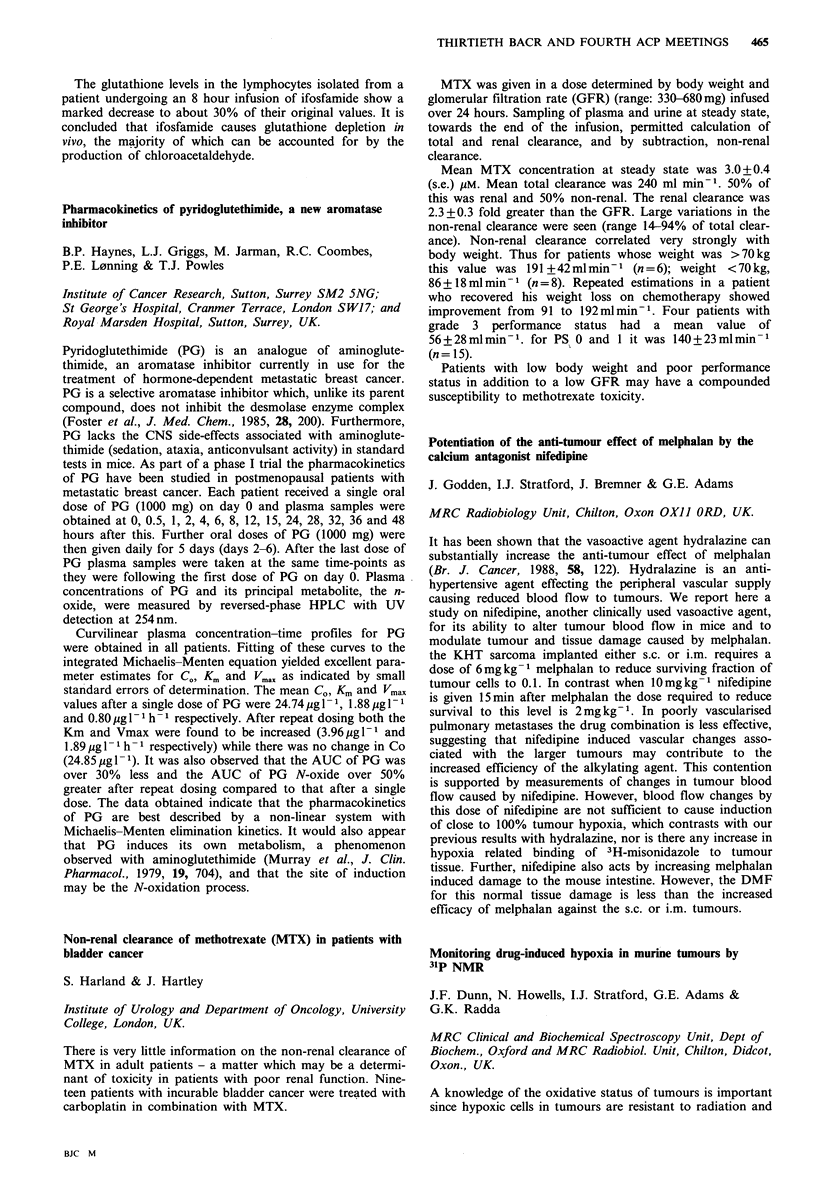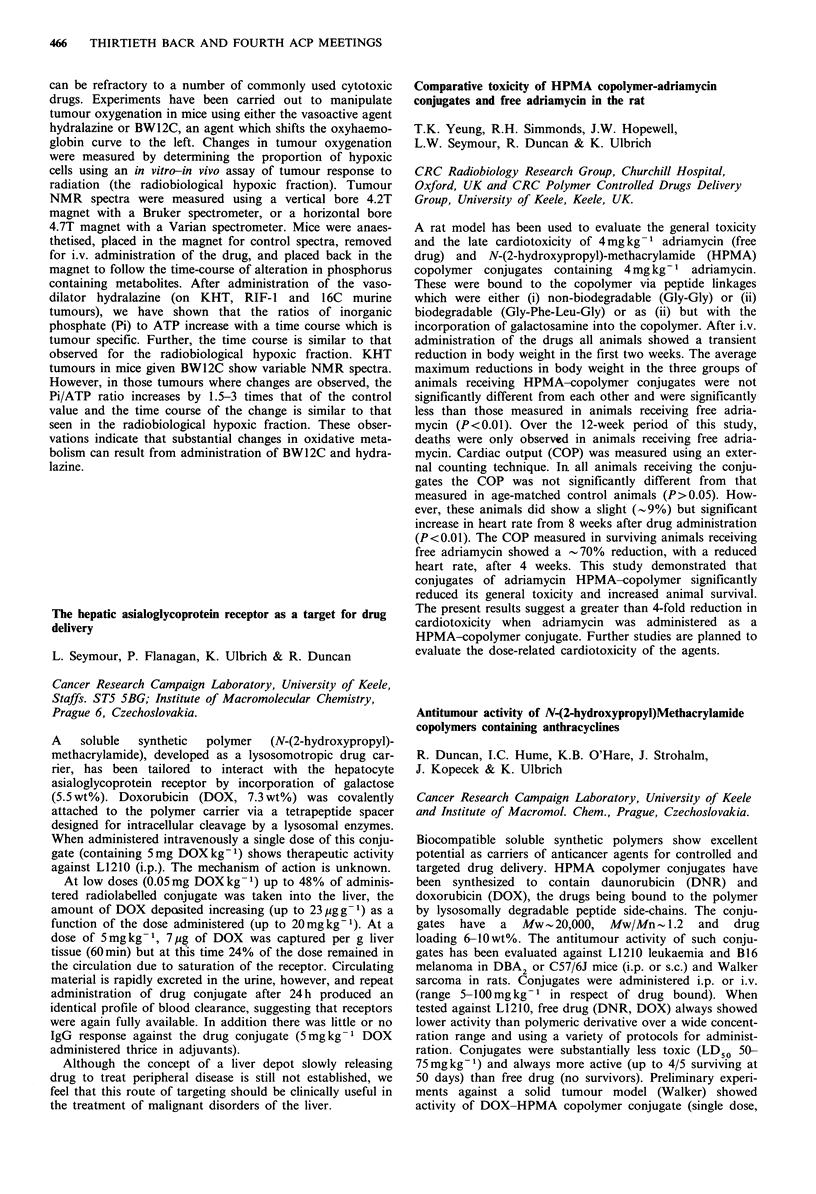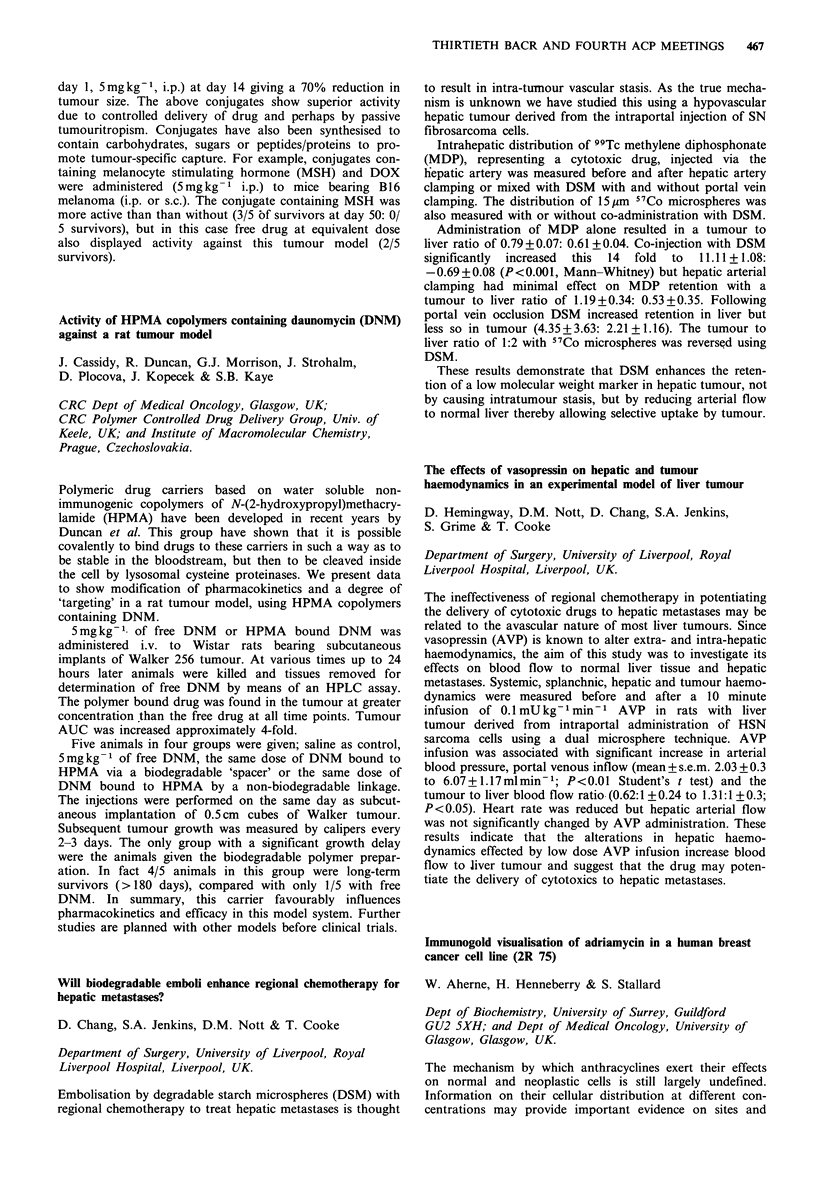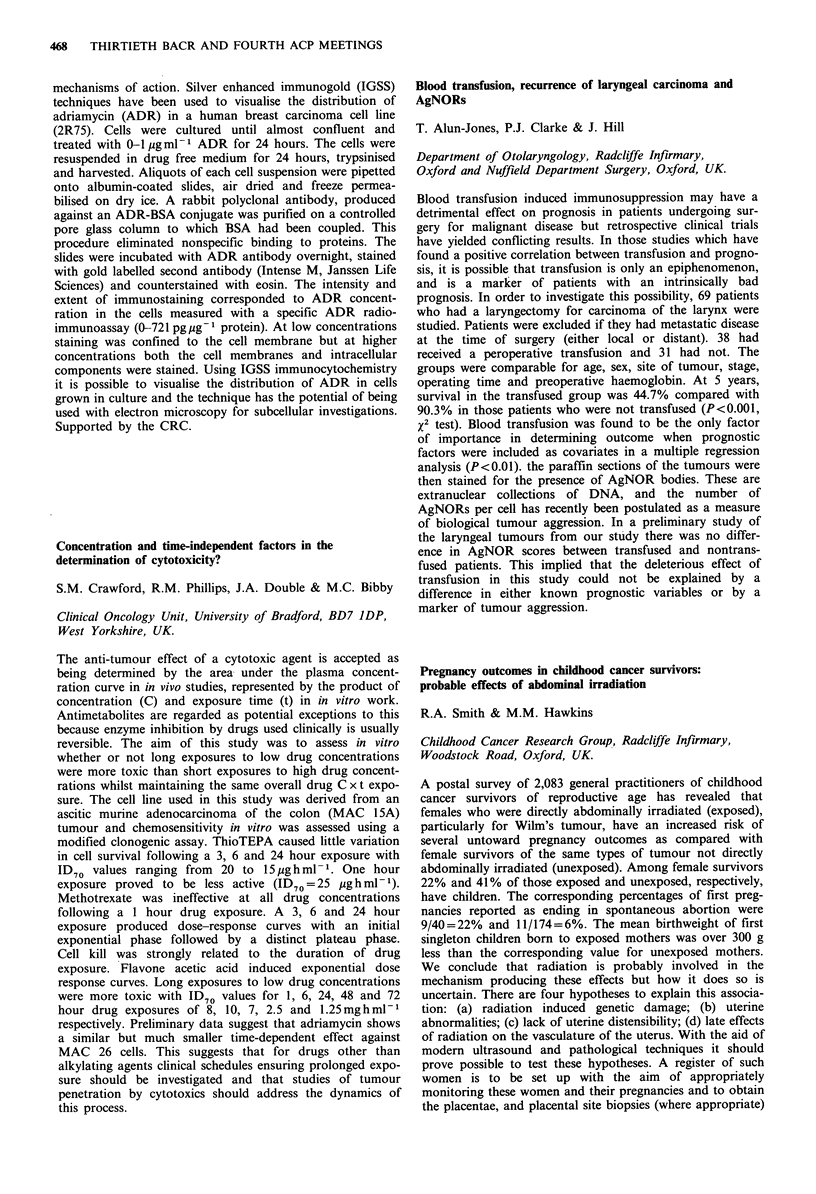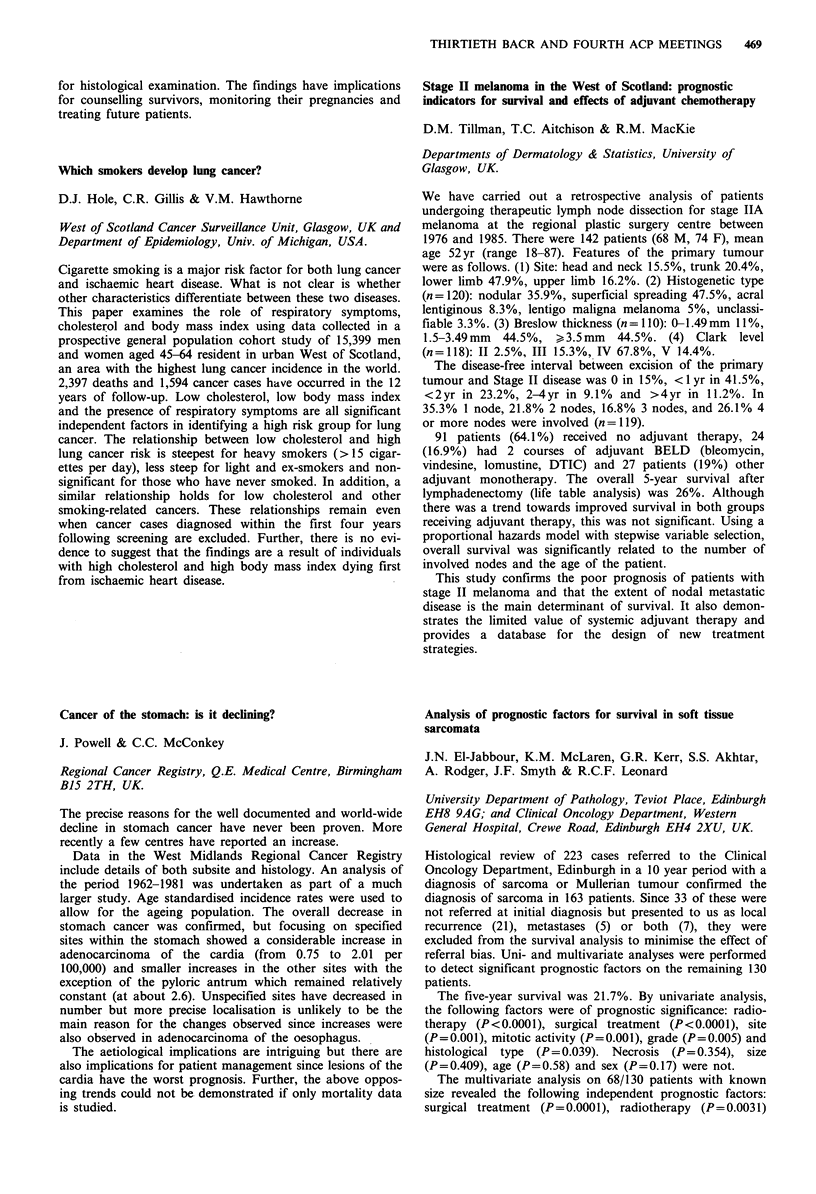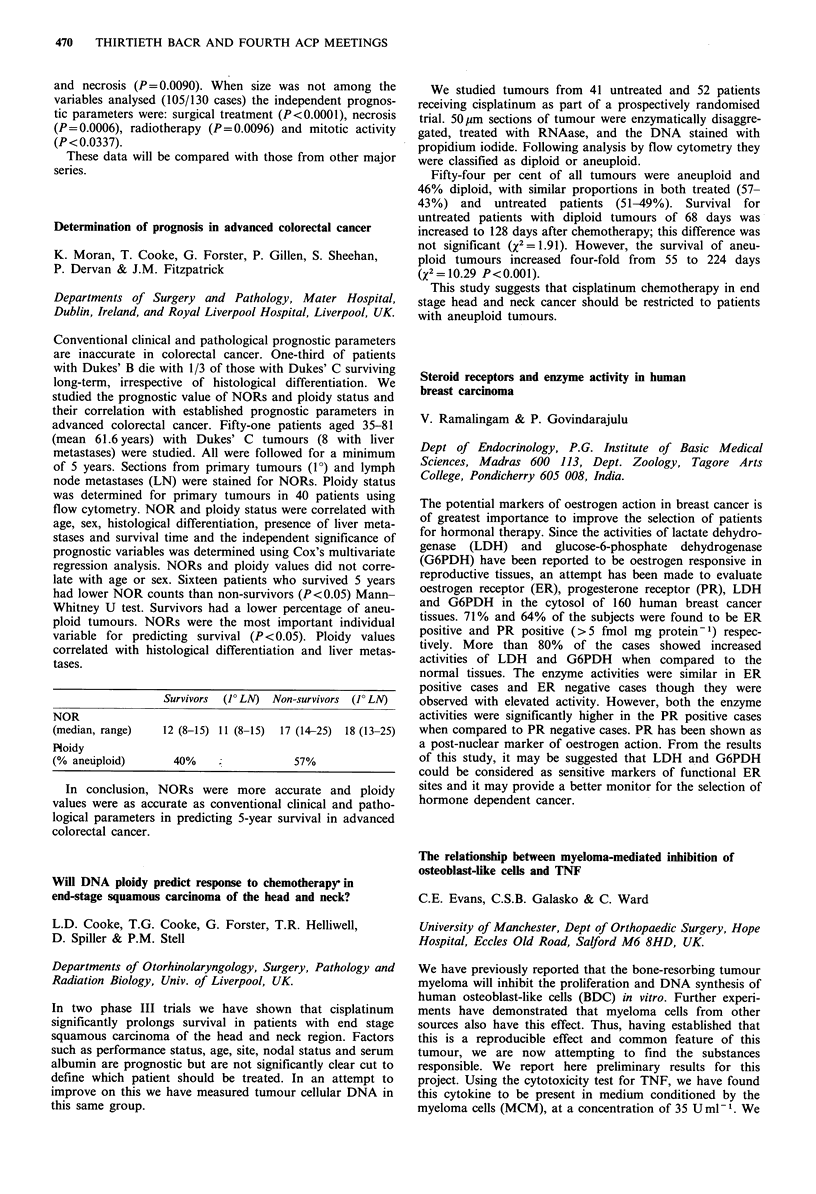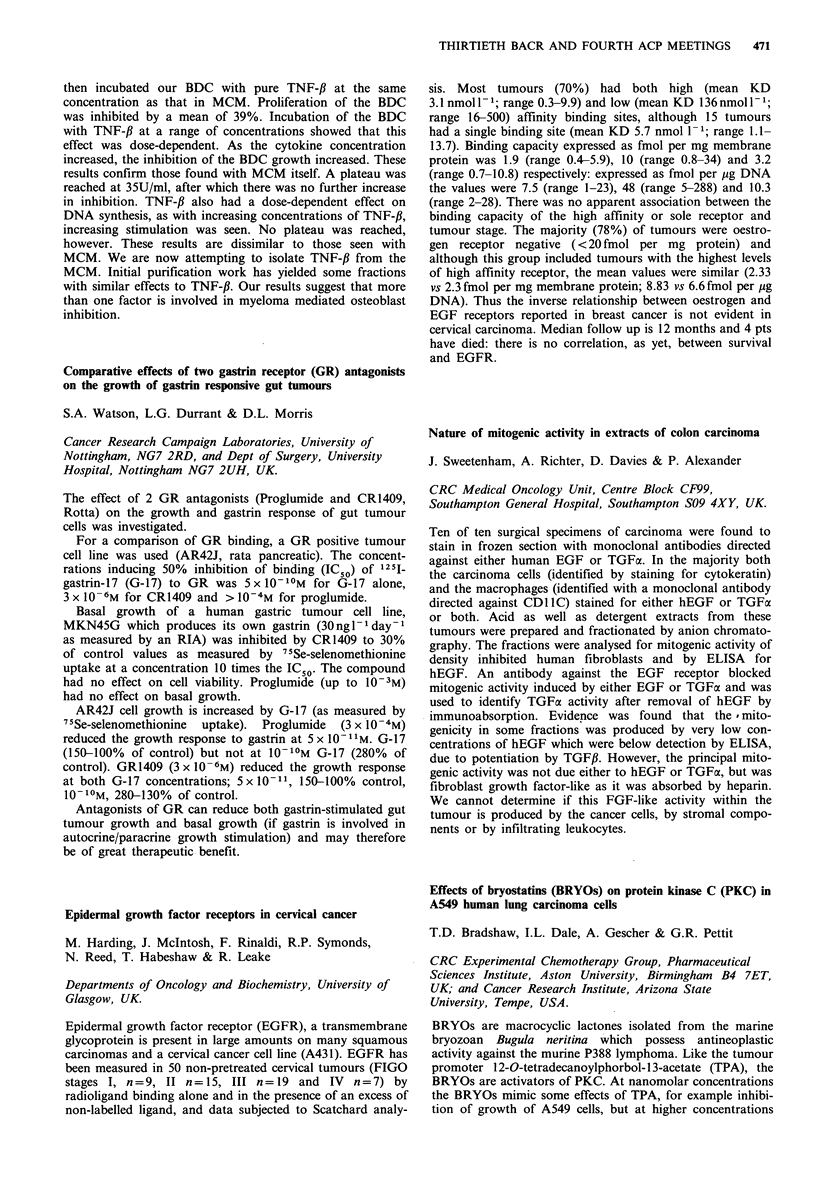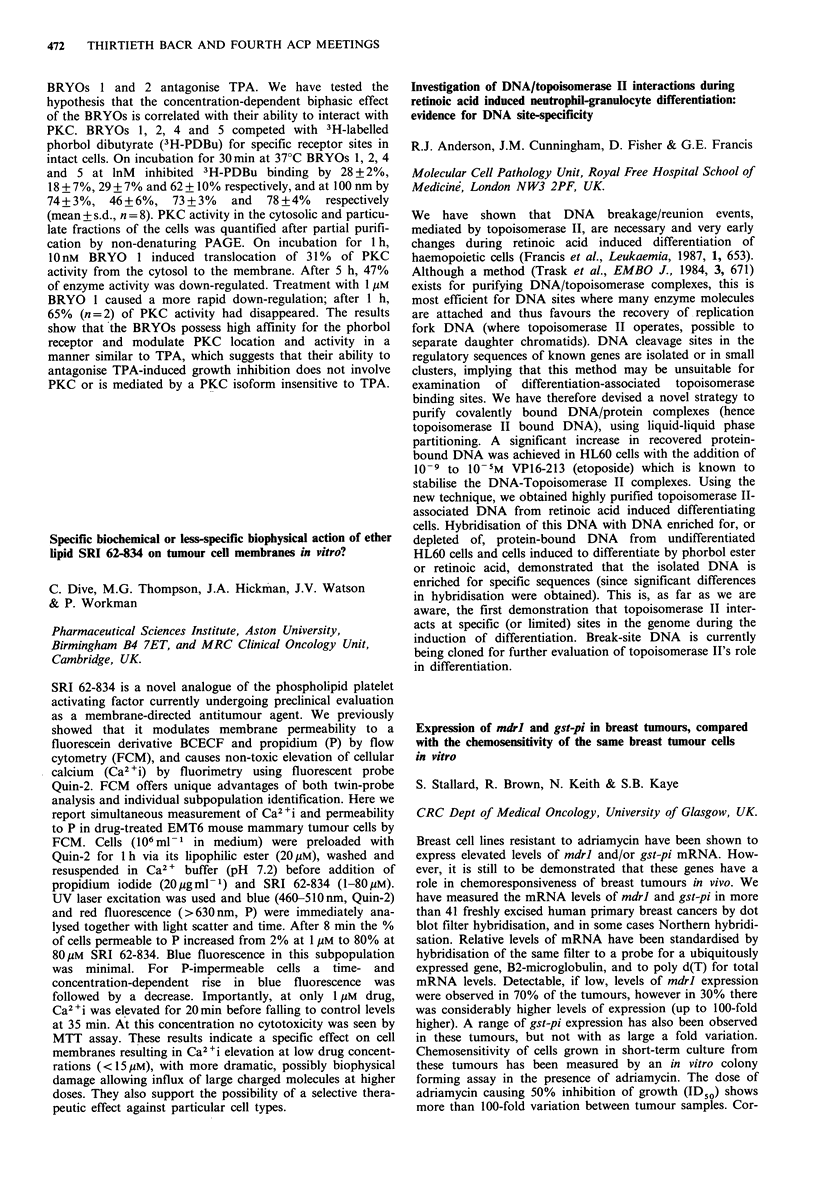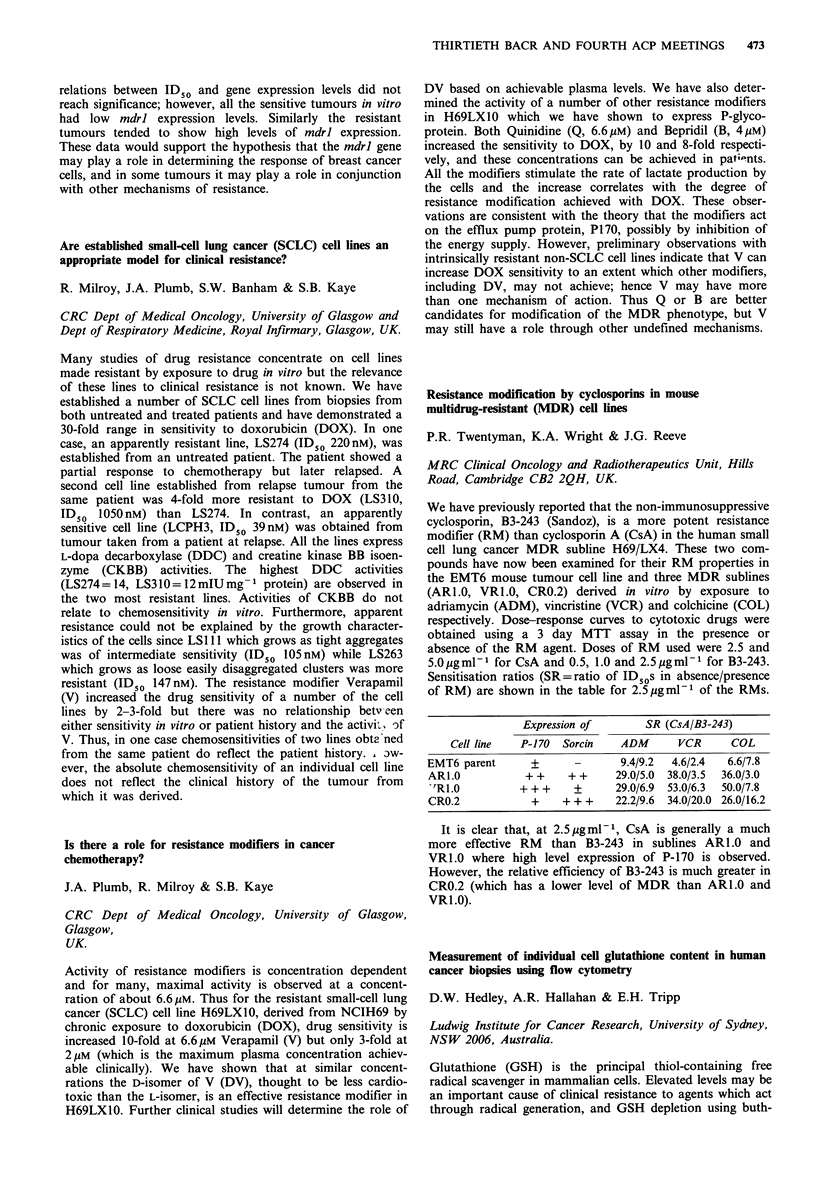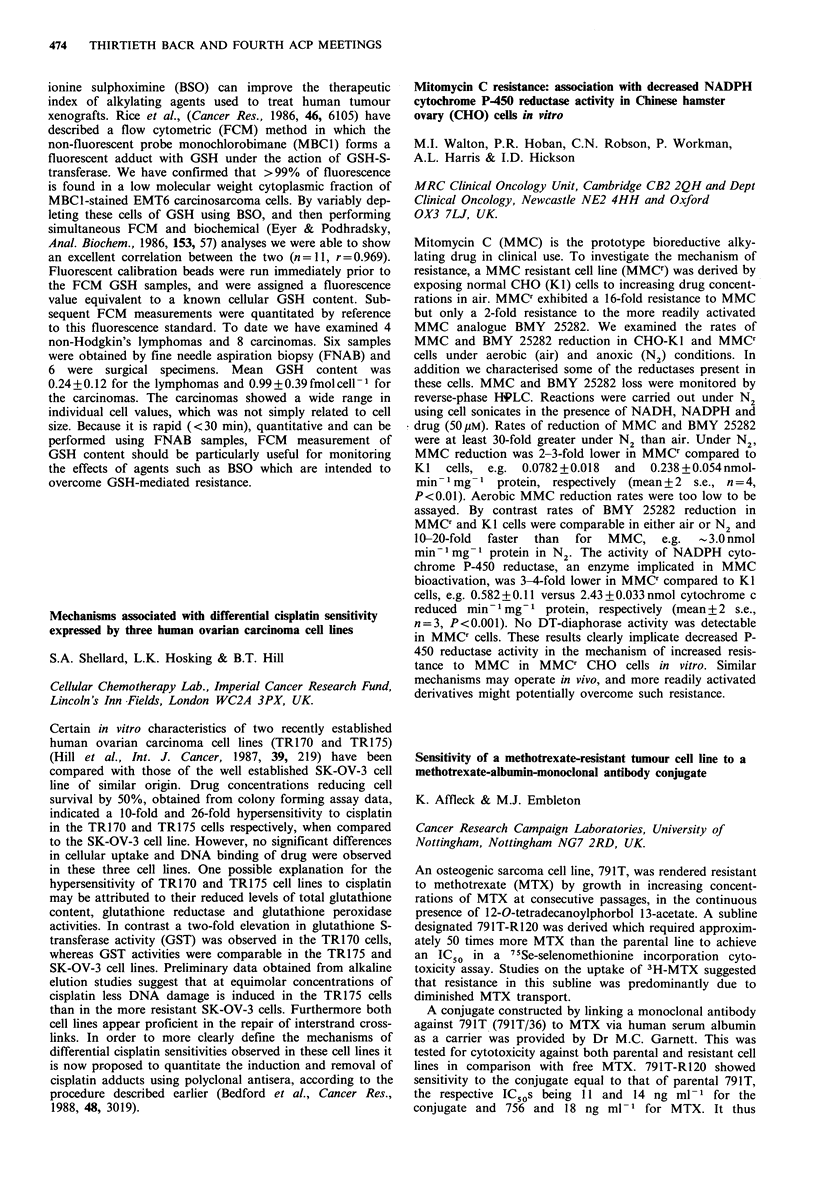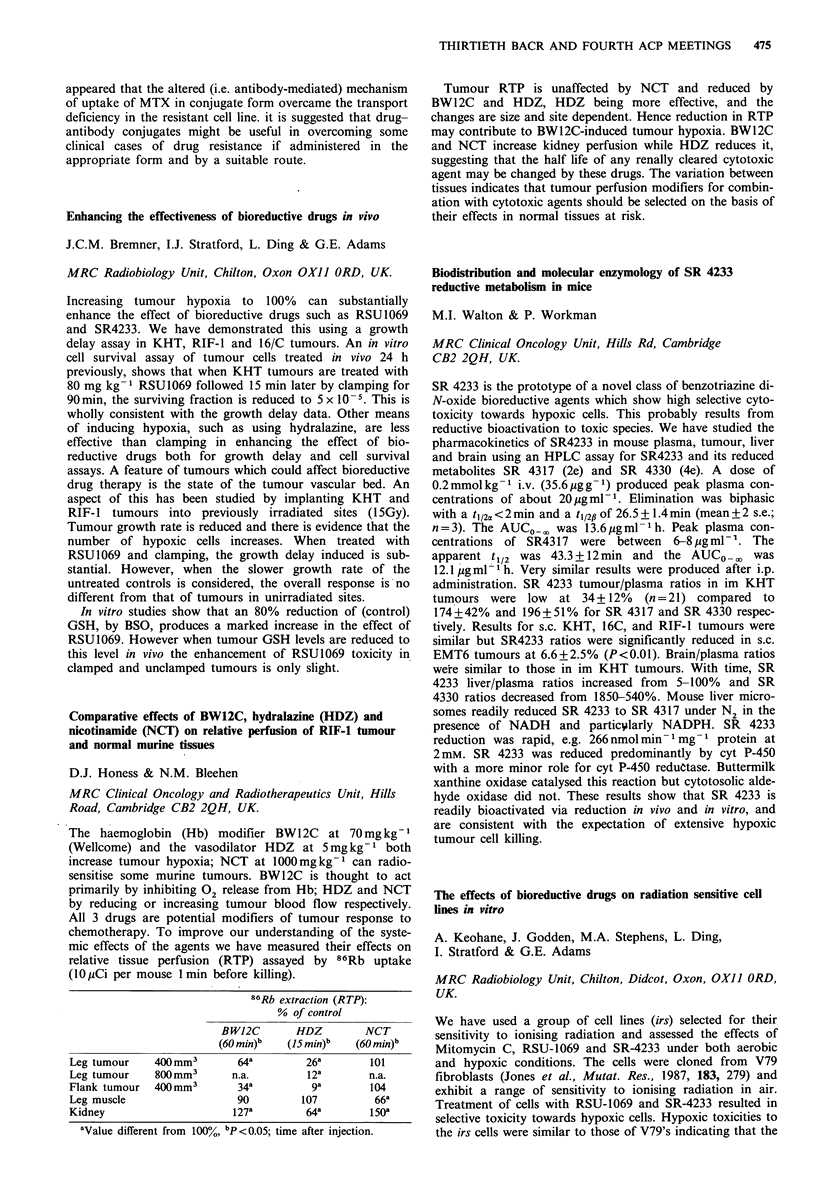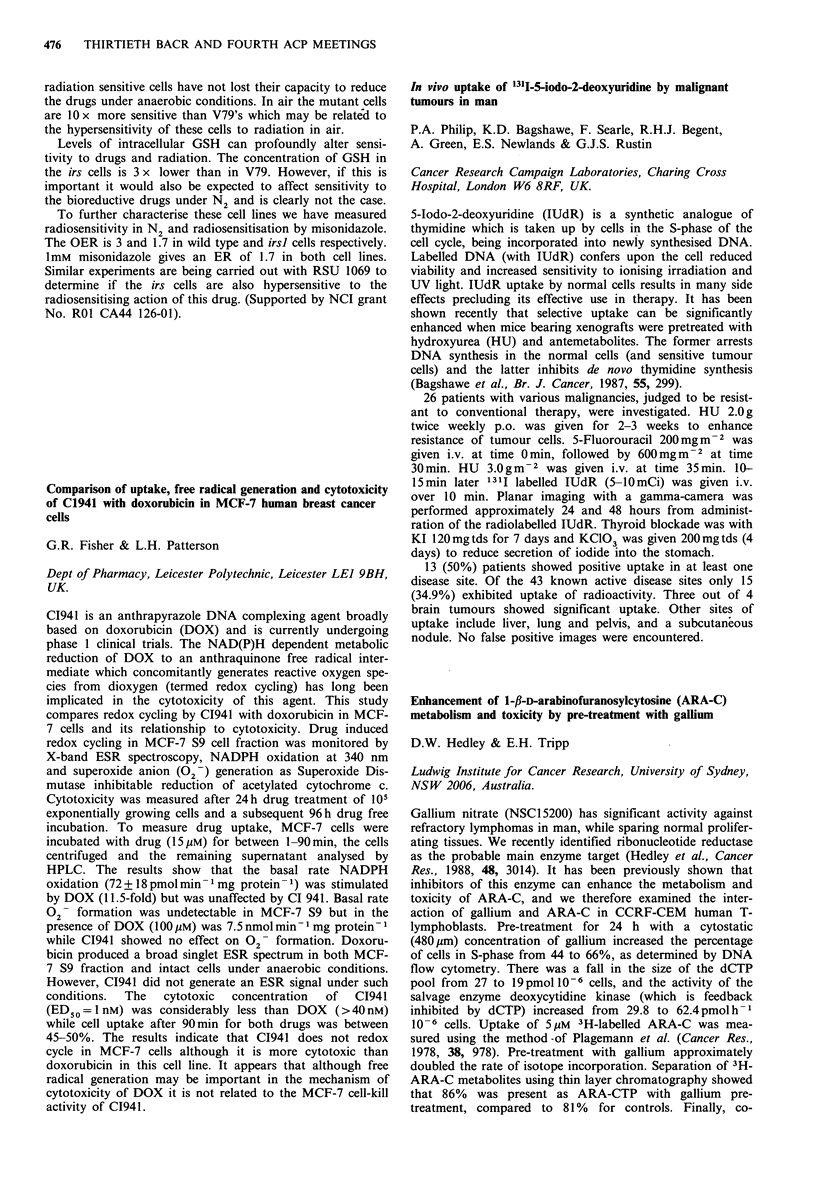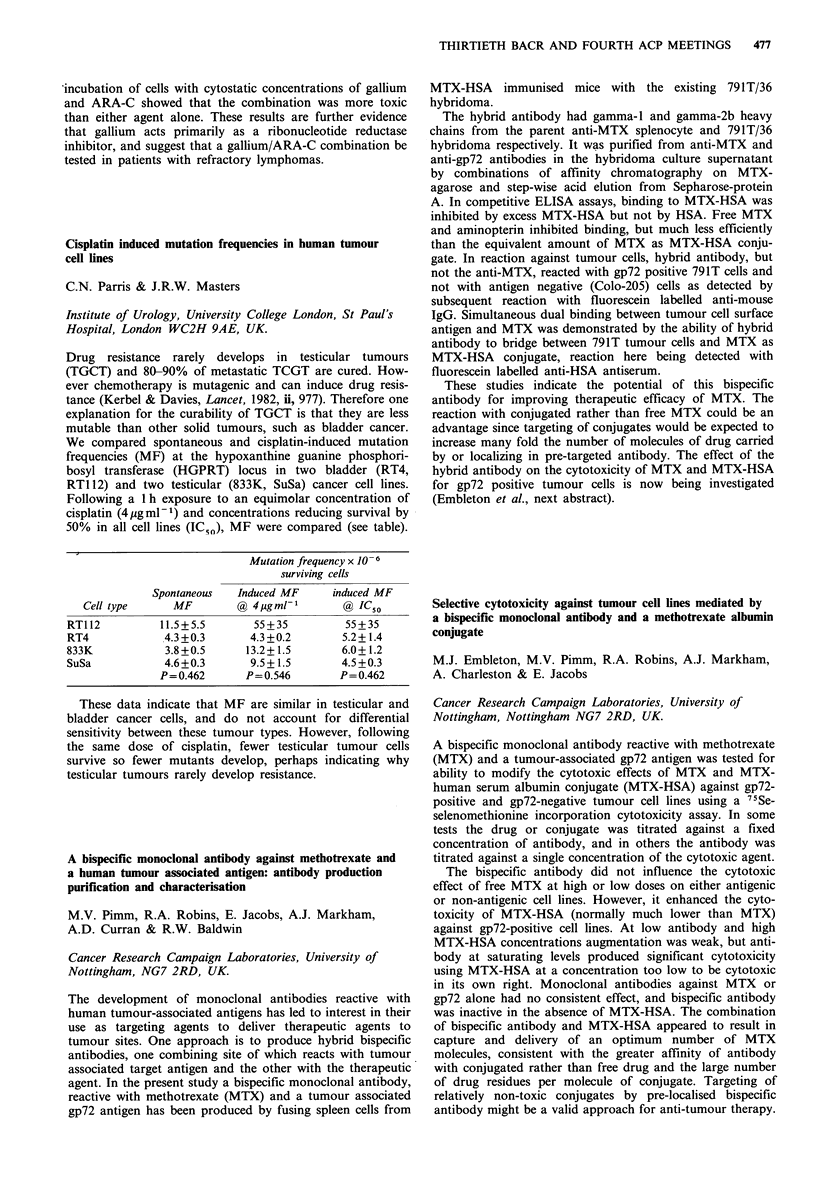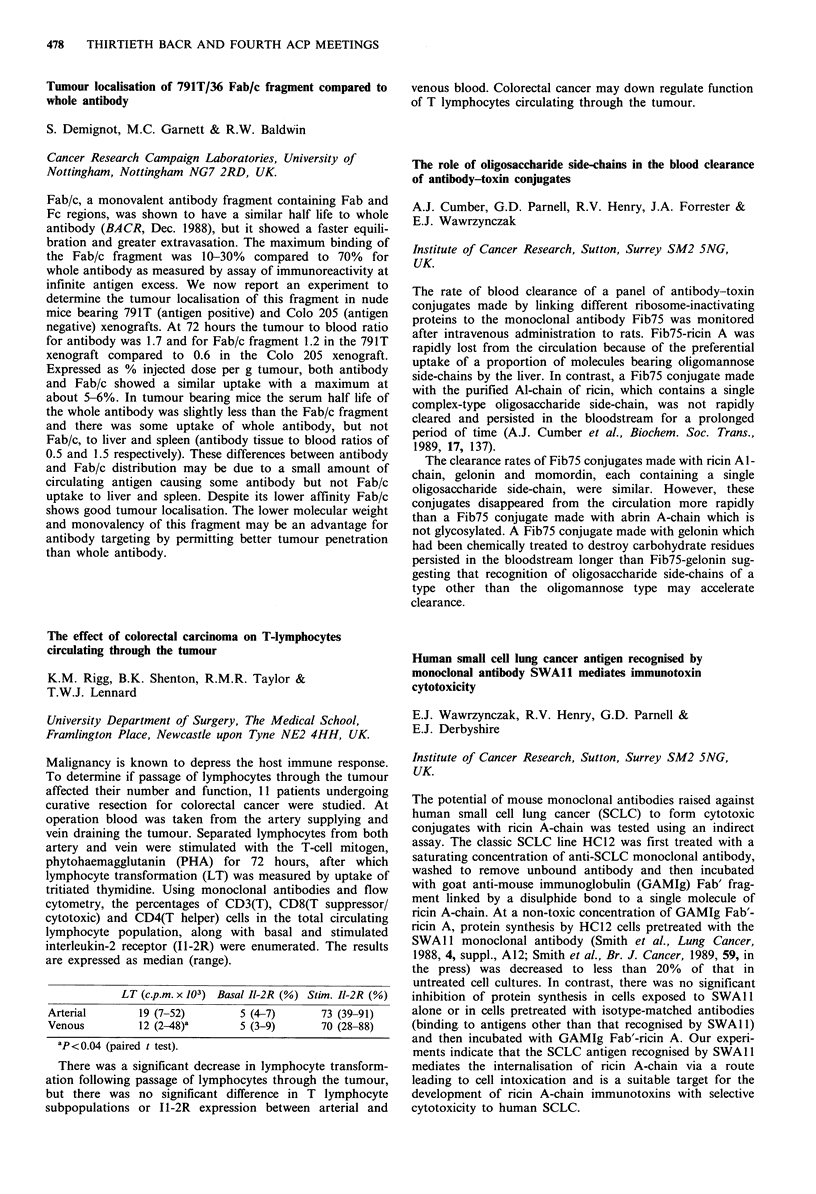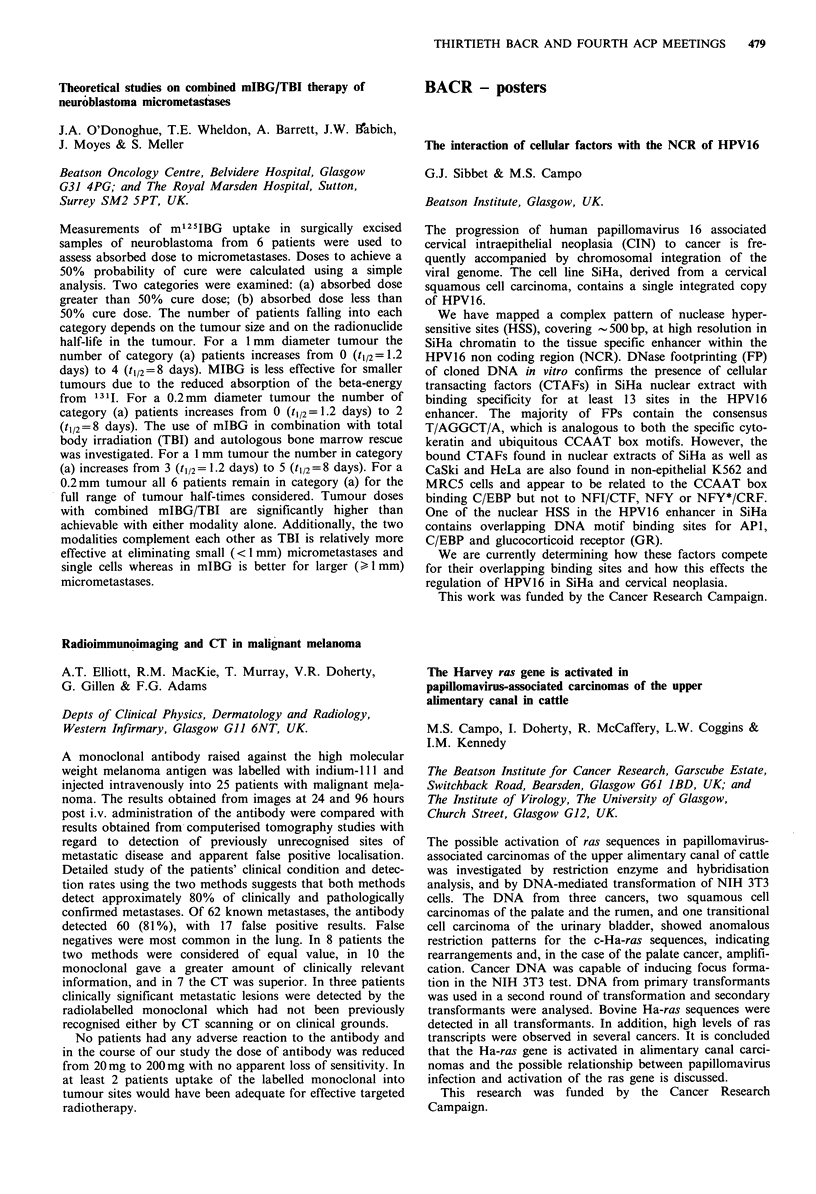# The thirtieth annual meeting of the British Association for Cancer Research and the fourth annual meeting of the Association of Cancer Physicians. 10-12 April 1989, Glasgow, UK.

**Published:** 1989-09

**Authors:** 


*Br. J. Cancer* (1969), **60**, 441–504 © The Macmillan Press Ltd., 1989

### The Thirtieth Annual Meeting of the British Association for Cancer Research and the Fourth Annual Meeting of the Association of Cancer Physicians, 10–12 April 1989

*Held at the University of Glasgow, UK.*

### Abstracts of Invited Papers

### Symposium on 'Current Approaches to Cancer Treatment and Patient Management' (ACP)

**Intrahepatic arterial chemotherapy with cytotoxic drug containing microspheres**

D.J. Kerr

*CRC Dept of Medical Oncology, University of Glasgow, UK.*

Given the relative paucity of novel, active cytotoxic drugs, it behoves us to optimise clinical usage of the currently available agents. The concept underlying regional chemotherapy depends on the genesis of high drug concentrations in the organ harbouring tumour deposits with a potential for decreased systemic concentrations and hence toxicity. If the drug was administered into an artery in microcapsular form, there could be an additional arterial embolic effect. In association with N. Willmott (Dept of Pharmacy, Strathclyde University), C. McArdle and J. Goldberg (Dept of Surgery, Glasgow Royal Infirmary) I have conducted a series of pharmacokinetic studies of intrahepatic arterial 5-fluorouracil (5-FU) in combination with adriamycin loaded albumin microspheres (1% drug w/w) in patients with colorectal hepatic metastases. Treatment was administered via an intrahepatic arterial portacath with a subcutaneous administration port. Using $^{99}Tc^{m}$-labelled albumin microspheres it has been possible to demonstrate that co-infusion of the vasoconstrictor angiotensin II ($10\,\mu g\ min^{-1}$ for 4 min) significantly increases the delivery of microspheres to tumour relative to normal tissue. Peripheral venous plasma concentrations of 5-FU are similar following bolus administration by intravenous or intrahepatic arterial (+ microspheres and angiotensin II) routes. There is a significant inverse correlation between systemic clearance of 5-FU and white cell/platelet nadirs. Subsequent studies showed that infusing 5-FU over 2 h significantly reduced systemic concentrations comparing 1V and 1A routes. The main problem with albumin microspheres is the small drug payload (1% w/w) and therefore we are continuing our studies with ethylcellulose microspheres containing mitomycin C (60% w/w).

**The principle of 'targeted' radiotherapy as illustrated by experience with meta-iodobenzylguanidine**

L.S. Lashford

*The Imperial Cancer Research Fund & The Hospital for Sick Children, London, UK.*

The iodinated benzylguanidine mIBG is actively transported into tissue of sympathetic origin, creating an opportunity for selective radiation delivery to chromaffin tumours. The success of this approach is dependent on differential dose delivery to tumour sites and normal organs. Detailed pharmacokinetic data has been obtained from studies in the sub-human primate, the common marmoset, and children with neuroblastoma. These studies demonstrate that the compound undergoes a period of rapid renal excretion, accounting for a mean of 13% of the dose in the first 3 h. Uptake is invariably noted in myocardium, liver, and salivary tissue, resulting in an estimated mean whole body dose of $0.4–0.5\,mGy\,MBq^{-1}$ (range 0.2–0.5) and hepatic dose of $1.5\,mGy\,MBq^{-1}$ (range 0.2–4.6). Animal studies suggest that whole body radiation dose delivery can be predicted within +30 to −20% by tracer scintigraphy. This hypothesis is currently being tested in the UKCCSG phase 1 of mIBG in chemoresistant neuroblastoma. However, animal studies also suggest that whole body radiation dose delivery may significantly increase with repeat administrations of mIBG ($P=0.003$). Mathematical modelling of these data, in combination with information on levels of isotope in resected tumour, indicates that tumoricidal doses may be delivered in some patients without marrow rescue. Strategies for improving tumour dose delivery are under investigation, and include changing the isotope to Auger electron emitters ($^{125}I$) and alpha emitters ($^{211}At$) and by manipulating tumour storage of the compound (e.g. calcium channel blockade). These strategies will be discussed.

**Intraperitoneal chemotherapy for minimal residual ovarian cancer. Experience with cisplatin and carboplatin**

W.W. ten Bokkel Huinink, R. Dubbelman, A. van Wijk, E. Aartsen, J.G. McVie, H. Franklin, S. Rodenhuis & W. v.d. Vijgh

*The Netherlands Cancer Institute, Amsterdam; Free University of Amsterdam, The Netherlands.*

Intraperitoneal (i.p.) chemotherapy for cancer confined to the abdominal space may have a pharmacological advantage if, as has been shown in animal models, an increased dose may indeed overcome secondary resistance, e.g. of ovarian cancer to cisplatin. In order to test this hypothesis we treated 32 patients (pts) with i.p. cisplatin with doses of 60–150 $mg\,m^{-2}$ i.p. in 21 dialysis fluid. After a phase I study with carboplatin which defined the maximum tolerated dose as 700 $mg\,m^{-2}$, 28 pts were treated with 6 courses at a dose of 650 $mg\,m^{-2}$. A Tenckhoff catheter was installed by laparoscopy for the i.p. chemotherapy and fluid distribution in the abdominal cavity was assessed after 6–10 days. Pharmacokinetic studies revealed that indeed cisplatin and carboplatin given i.p. may reach 2–10-fold higher concentrations in the abdominal space than when given intravenously. The AUC-uf.p./AUC-uf.pr is 17%. The peritoneal clearance of carboplatin is $3.3\,ml\,min^{-1}\,m^{-2}$. In patients with minimal residual disease ovarian cancer, all having undergone heavy pretreatment with cisplatin based chemotherapy, 10 out of 32 pts evaluable for response, who had been treated with escalated doses of cisplatin i.p., reached a complete remission (CR). Of 28 pts treated with carboplatin only 3 reached a CR.

In conclusion, i.p. chemotherapy for tumours confined to the abdominal space may indeed offer a pharmacological advantage and have an increased therapeutic ratio. However, this kind of treatment is cumbersome and should be still regarded as experimental: Randomised studies for final proof of the advantage of i.p. chemotherapy above continued systemic treatment are in progress.

### The role of growth factors in patient management

N. Thatcher

*Christie and Wythenshaw Hospitals, Manchester, UK.*

Haemopoietic growth factors (colony stimulating factors – CSFs) have been known for many years to exert critical effects on bone marrow precursor cells. Only recently with the advent of recombinant DNA technology have sufficient quantities of these factors – erythropoietin (EPO) granulocyte-macrophage CSF (GM-CSF), granulocyte CSF (G-CSF), macrophage CSF (M-CSF), interleukin-2 (IL-2) and 3 (IL-3) – become available for preclinical testing. Some of these EPO (GM-CSF, G-CSF, IL-2) have recently entered clinical evaluation.

The myeloid CSFs, by increasing the number and functional activity of granulocytes and macrophages in patients, would be expected to counter infection and possibly have an anti-neoplastic effect. Infusions of rG-CSF (without any toxic side-effects) have been shown to restore functional neutrophils in patients undergoing myelosuppressive chemotherapy for small cell lung cancer with consequent reduction in severe infective episodes (Bronchud *et al.*, *Br. J. Cancer*, 1987, **57**, 809). Significant increases in total leucocyte, neutrophil and eosinophils have also been obtained with rGM-CSF infusions with stabilisation and response of previously progressing metastatic solid tumours (Steward *et al.*, *Br. J. Cancer*, 1989). However, GM-CSF in this study was associated with pyrexia, bone pain and pruritus. Other clinical applications of CSFs include: EPO, stimulating red cell production in end stage renal failure, in chronic disease, e.g. Rh, arthritis and in neoplasia; also at high dose, stimulation of platelet production in marrow failure after chemotherapy. The myeloid CSFs could be of use in marrow failure (idiopathic, neoplastic, iatrogenic), improve host defence after major trauma (burns), and also in the treatment of established infections associated with leucopenia and/or reduced myeloid function, e.g. in AIDS, post-chemotherapy patients. Myeloid CSFs may also augment marrow recovery after transplantation.

The therapeutic use of IL-2, the T-cell growth factor was established by Rosenberg *et al.* (*NEJM*, 1987, **316**, 889). The use of high dose rIL-2 with or without LAK (lymphokine activated killer) cells obtained by multiple leucophoresis, *in vitro* incubation with rIL-2 and subsequent reinfusion, resulted in tumour responses particularly in resistant metastatic tumours, e.g. hypernephroma and malignant melanoma. Other schedules of rIL-2 (constant infusions, alternate day administration without LAK cells) have been developed. Tumour responses with rIL-2 have been confirmed by ourselves and others in malignant melanoma and hypernephroma.

This new era of oncology in which there is immunotherapy and the abrogation of chemotherapeutic induced bone marrow suppression is one of the most exciting developments in recent years.

### The Bagshawe Lecture
### (Sponsored by Upjohn UK Ltd)

### While waiting for the human genome to be mapped

K.D. Bagshawe

*Cancer Research Campaign Laboratories, Dept of Medical Oncology, Charing Cross Hospital, Fulham Palace Road, London W6 8RF, UK.*

See full paper in this issue.

### Symposium on 'The biological basis for breast cancer treatment' (BACR/ACP)

### Epidemiology of breast cancer as a guide to biological understanding

P. Boyle

*Unit of Analytical Epidemiology, International Agency for Research on Cancer, 150 Cours Albert-Thomas F-69372 Lyon, France.*

Breast cancer is the commonest form of cancer occurring in women throughout the world apart from skin cancers. Although traditionally considered as a disease of advanced lifestyle and affluence, developing countries are not spared breast cancer, where it is estimated that over 14% of the total cancer burden in 1980 comprised tumours in the breast. In developed countries there are apparent inconsistencies between incidence and mortality rates which could point to important biological aspects of breast cancer which remain to be unmasked by closer study. The suggestion from several countries of downward tumours or stabilisations in breast cancer risk among younger groups of women is one that defies current knowledge of risk factors and requires close surveillance. It is generally agreed that nulliparous women have about a 50% excess of breast cancer over parous women. An early age at first birth appears to confer protection, while breast cancer risk appears to be increased by an early age at menarche and a later age at menopause. The risk of breast cancer seems to be increased by having had a previous cancer in the contralateral breast or in the ovary and seems to be reduced by having had a surgical menopause at an earlier age. There is still lack of concordance regarding the role of risk factors such as lactation, oral contraception usage, first trimester abortion, dietary consumption and alcohol use in the aetiology of cancer of the breast. Hitherto, research for breast cancer risk factors has almost exclusively focused on the function of the ovary and related phenomena. The risk factors mentioned above are almost certainly some proxy for the mechanism which leads to the appearance of a clinical cancer. Despite extensive research, the role of endogenous hormones in the aetiology of breast cancer is unclear. Recent analyses of breast cancer case-control studies reveal clearly that some of the risk factors for breast cancer affect breast cancer arising at different ages differentially. This will be illustrated by recent data relating to age at first birth, parity and obesity.

**Biological environment of the cancerous breast**

D.P. Rose

*Division of Nutrition and Endocrinology, American Health Foundation, New York, USA.*

The microenvironment of the breast cancer cell is derived from contributions by the host, and by the tumour itself. Nutrients are delivered via the circulation, together with steroid and protein hormones, thyronines and other growth factors. Fatty acids are metabolised 'on-site', including linoleic acid, which serves as a precursor for prostaglandin synthesis, and influence cancer cell membrane fluidity, receptor sites and cell proliferation. Although dietary fats may promote breast cancer growth, those rich in omega-3 fatty acids appear to have an inhibitory effect, perhaps because they are inhibitors of the enzyme prostaglandin synthetase. Adipose tissue is a major site for the conversion of $C_{19}$ steroids to oestrogens, and production of these mammary cancer-stimulating hormones is increased in obesity, itself a recognised risk factor for post-menopausal breast cancer, and may also be enhanced in the cancer-bearing breast. Breast cancer cells are frequently capable of performing the metabolic interconversion of steroids, including both oestrogen synthesis and deconjugation to produce the biologically active hormone. The synthesis of polyamines from ornithine appears essential for the growth of cancer cells, at least in rat mammary tumour models, perhaps because the polyamines regulate the production of tumour-derived protein growth factors. The complexity of polypeptide growth factors synthesised within tumour cells, and secreted into the microenvironment, includes the transforming growth factor (TGF)-$\alpha$ family, which are structurally related to epidermal growth factor and bind to the EGF receptor, TGF-$\beta$, the insulin-like growth factors, and platelet-derived growth factor. As a clearer understanding of the biochemical interrelationships which create the biological environment of the cancerous breast emerges, it offers exciting new approaches to breast cancer therapy.

**Growth factor receptor expression in human tumours**

W.J. Gullick, F.J. Lofts, N.L. Tuzi, D.M. Barnes & P. Quirke

*ICRF Oncology Group, 3rd floor, MRC Cyclotron Building, Hammersmith Hospital, Du Cane Rd, London W12; ICRF Oncology Unit, Guys Hospital, London SE1; Dept of Pathology, University of Leeds, Leeds LS2, UK.*

Examination of the mechanisms by which normal cells regulate their growth, and comparison of these with those of cancer cells, has revealed molecular changes which may represent some of the fundamental causes of malignant transformation. Tumour cells frequently produce polypeptide growth factors in an apparently unregulated way which may stimulate their own, or adjacent cells to grow. The receptors for such factors are also frequently affected, either by mutation or by greatly elevated levels of protein expression.

We have investigated the level of expression of one such molecule, the c-*erb*B-2 protein, in a variety of human normal and malignant tissues using immunohistological staining. The c-*erb*B-2 protein is expressed on many normal tissues including those derived from all three germ layers. Elevated expression, generally as a consequence of gene amplification, has been found in 10–30% of breast and stomach adenocarcinomas. The c-*erb*B-2 protein is also expressed in greater than 90% of comedo type ductal carcinoma *in situ* but not in other histological types of CIS. One consequence of this change may be to accelerate the growth of such cells. This event may have implications in diagnosis of early stage disease, in prediction of disease progression and in tumour response to drug treatment or radiotherapy.

An additional biochemical consequence of overexpression of c-*erb*B-2 is reduction of gap junctional permeability which could affect the response of such cells to other oncogenic changes.

A model for the mechanism of growth factor receptor activation will be presented.

**Biological indices of progression in breast cancer**

R. Leake

*Department of Biochemistry, University of Glasgow, Glasgow G12 8QQ, UK.*

Several clinical features of breast cancer are known to give good prognostic information (e.g. node involvement, disease-free interval). Biological indices of prognosis have been disappointing. Soluble oestrogen receptor (ER) status gives some information about total survival but a proportion of ER+ still relapse and die quickly. Combining soluble and nuclear oestrogen receptor status gives much better prognostic discrimination. Assessment of the heterogeneity of receptor distribution further improves the discrimination. However, because of this heterogeneity, no one biological feature is able to give a complete description of the growth potential of an individual tumour. Presence of epidermal growth factor receptor (EGF-R) is a positive indication of poor prognosis though not all laboratories find the same prognostic significance. Elevated levels of $\alpha$-TGF are found in some breast cancers and combining $\alpha$-TGF levels with EGF-R concentration may improve the prognostic discrimination. Increased aneuploidy is associated with poorer prognosis. Increased expression of the *neu* (*erb* B2) gene is observed in a large proportion of ductal carcinoma *in situ*. This may be an indication of those tumours which are likely to become invasive. Amplification of the *myc* gene is also reported in some breast cancers and may have significant prognostic value. Overall, biological markers of prognosis are most likely to be accurate when several independent markers are measured on each tumour. Quantitation of markers should include some measure of heterogeneity. Ideal markers will work on paraffin embedded, fixed tissue or on representative fine needle aspirates.

### The 1989 Walter Hubert Lecture

**Does biological understanding influence surgical practice?**

R.W. Blamey

*City Hospital, Nottingham, UK.*

See full paper in this issue.

### Symposium on 'The molecular and cellular biology of cell–cell interaction' (BACR; sponsored by Bristol Myers Oncology UK Ltd)

### Junctional communication and cellular differentiation

J.D. Pitts, M.E. Finbow & E. Kam

*Beatson Institute for Cancer Research, Garscube Estate, Bearsden, Glasgow G61 1BD, UK.*

Cytoplasmic continuity between adjacent cells in the tissues of metazoan animals is provided by the aqueous channels of gap junctions, sieve-like structures which are freely permeable to small ions and molecules ($M_r > 1000$). Coupled cells surrender independence through the coordinating effects of shared intracellular pools of ions, metabolites, cofactors, second messengers, etc., while retaining individuality through the expression of different macromolecules. The junctional channels are made of a tissue invariant, evolutionarily conserved 16k protein but the formation and maintenance of active coupling also requires one or more connexins, a family of tissue-specific proteins ranging in size from 21k to 75k. The connexins may interact with tissue-specific proteoglycans and account for specificity of junction formation. Such specificity leads to the production of communication compartments, groups of cells joined by junctions but separated by reduced trans-boundary coupling from cells in adjacent compartments. Such compartmentation occurs during embryonic development, probably to allow the necessary expression of cellular differentiation. The patterns of communication have been mapped in detail in mouse skin, in normal, in hyperplastic conditions and in tumours. The results suggest that in small compartments, like those of the epidermis, where only a proportion of the cells responds to a systemic stimulus (e.g. growth factor) which affects activity through second messengers, homeostatic pressure through loss of second messengers into non-responding cells, produces a modulating control related to compartment size. Such cell interactions, within compartments and also between compartments, may provide a growth restraint for maintaining the required cell numbers in different parts of a tissue.

### Oncogene and growth factor activity in tumour initiation promotion and progression

A. Balmain, B. Bailleul, S. White, A. Surani & J. Jorcano

*Beatson Institute, Glasgow; IAPGR, Cambridge and Univ. of Madrid, Madrid, Spain.*

Among the most frequent molecular changes which have been detected in human tumours are the activation of *ras* genes by point mutations and the loss of heterozygosity at a number of different chromosomal loci. It is extremely difficult with human tumours to evaluate the causal nature of these events in tumour development, or to determine the stage of carcinogenesis at which they occur. Mouse skin carcinogenesis has been a valuable model system for the study of these questions. It has previously been shown that initiation of carcinogenesis can be induced by rare carcinogen-specific mutations in the cellular H-*ras* gene. In this case treatment with a tumour promoting agent is necessary for the development of papillomas, but if the mutant *ras* gene is present in all epidermal cells, as in transgenic mice, the papillomas appear spontaneously. This is compatible with models which suggest that tumour promoters remove the growth inhibitory effects of normal cells. The additional events required for progression to carcinomas are unknown, but experiments using hybrid mice show that progression is frequently accompanied by loss of alleles on mouse chromosome 7. This may be associated with the development of homozygosity at mutant *ras* alleles or the loss of tumour suppressor genes.

### Epithelial mesenchymal interactions in growth and differentiation of normal and transformed skin keratinocytes

N.E. Fusenig, D. Breitkreutz & P. Boukamp

*German Cancer Research Center, D-6900 Heidelberg, FRG.*

Epithelial mesenchymal interactions are important regulators in embryonic development and responsible for maintenance of tissue homeostasis as well as its loss in carcinogenesis. Differentiation and malignancy are complex tissue phenomena which cannot be fully understood at the single cell level. Model systems for mouse and human skin keratinocytes have been developed to analyse both processes under *in vivo* and *in vitro* conditions providing also direct (cellular) and indirect (factor-mediated) mesenchymal influence. Normal epidermal differentiation strictly depends on mesenchymal signals irrespective of whether cell–cell contact is permitted. But also malignantly transformed mouse keratinocytes can still be induced to re-express rather normal keratinisation although subtle changes in the co-ordination of the differentiation programme become apparent. These alterations increase concomitantly with continued growth of cancer cells *in vivo* leading to a dysplastic and eventually invasive epithelium. These altered growth and differentiation phenomena occur independently of direct epithelial mesenchymal contact and thus are most probably mediated by diffusible factors. During early growth phases of malignant keratinocytes these factors may still be competent for controlling epithelial cell homeostasis comparable to the situation with benign tumour cells. Carcinoma cells, however, are obviously capable of modulating these mesenchymal interactions, leading to disturbances of tissue homeostasis and eventually to invasion and progressive tumour growth.

### The effect of surrounding normal cells on the growth and behaviour of cells transformed by viral oncogenes

F. Tato

*Dipartimento di Biologia Cellulare e dello Sviluppo, Università di Roma, 'La Sapienza', Roma, Italy.*

The abnormal growth of v-*myc* transformed fibroblasts is inhibited *in vitro* by surrounding normal cells, as long as the latter display a high degree of contact inhibition and density-dependent growth arrest. Cells transformed by v-*ras* and v-*src* are insensitive to this inhibition. Tumorigenicity in nude mice parallels this *in vitro* behaviour: v-*myc* transformed cells do not produce tumours, whereas v-*src* and v-*ras* transformed cells are strongly tumorigenic. When v-*myc* transformed muscle cells are co-cultivated with appropriate normal cells their growth is also inhibited and, furthermore, there is re-expression of the myogenic differentiation programme.

The behaviour of v-*myc* transformed cells thus resembles that of hybrids between tumorigenic and normal human cells. In these hybrids, studied by a number of groups, abnormal *in vitro* growth persists but tumorigenicity is lost, apparently in association with expression of the differentiation programme of the normal parent. The two sets of data suggest that an interaction with adjacent normal cells is important in inhibiting the expression of the transformed phenotype, both *in vitro* and *in vivo*.

**The generation of invasiveness in transformed cells represents an essential step of tumour progression**

W. Birchmeier

*Institut fur Zellbiologie, Universitätsklinikum Essen-Westdeutsches Tumorzentrum, Essen D-4300, FRG.*

The generation of invasiveness in transformed cells represents an essential step of tumour progression. We show first, that non-transformed MDCK epithelial cells become invasive when intercellular adhesion is specifically inhibited by the addition of antibodies against the cell–cell adhesion molecule Arc-1/uvormorulin; the separated cells then invade collagen gels and embryonal heart tissue. Second, MDCK cells transformed with Harvey and Moloney sarcoma viruses are constitutively invasive, and they were found not to express Arc-1/uvormorulin at their cell surface. These data suggest that the loss of adhesive function of Arc-1/uvormorulin (which is identical to L-CAM or E-cadherin) is a critical step in the promotion of epithelial cells to a more malignant, i.e. invasive phenotype. Similar modulation of intercellular adhesion might also occur during invasion of carcinoma cells *in vivo*.

**Role of proteoglycans and cell contact in stromal cell interaction in haemopoiesis**

M.Y. Gordon

*The Leukaemia Research Fund Centre, Institute of Cancer Research, 237 Fulham Road, London SW3 6JB, UK.*

The idea that stromal cells are essential for the control of normal haemopoiesis is generally accepted but poorly understood. We have exploited the finding that blast colony-forming cells (Bl-CFC) in human marrow adhere to pre-formed marrow-derived stromal layers *in vitro* and are stimulated to divide. This allows investigation of binding interactions between haemopoietic cells and stromal cells in the haemopoietic microenvironment; and of any growth factors that may be required for the proliferative response by Bl-CFC. The Bl-CFC are primitive cells that are ancestral to lineage-committed haemopoietic progenitors (Gordon *et al.*, *J. Cell Physiol.*, 1987, **130**, 150). They bind to stroma via a haemopoietic progenitor cell-adhesion molecule (HP-CAM) which does not require serum or divalent cations but does require heparan sulphate in the extracellular matrix (ECM) produced by the stromal cells. The minimal integrin recognition sequence (RGD) is not necessary. HP-CAM is masked by sialic acid or not expressed by more mature lineage-committed cells. Proliferation by immobilised Bl-CFC might be stimulated by growth factors sequestered by the ECM. Granulocyte-macrophage colony-stimulating factor (GM-CSF) binds to glycosaminoglycans (Gordon *et al.*, *Nature*, 1987, **326**, 403) but not to heparin-sepharose to differing extents. The different binding properties of haemopoietic progenitor cells and of their corresponding growth factors suggests that the ECM produced by stromal cells can present growth factors to immobilised target cells. Binding interactions with stroma are disrupted in leukaemia (Gordon *et al.*, *Nature*, 1987, **328**, 342). This abnormality may be related to the accelerated release of malignant cells into the blood and facilitate extramedullary haemopoiesis.

### Abstracts of members' proffered papers

### ACP–oral presentations

**The natural history of low grade non-Hodgkin's lymphoma (LG NHL) development of discrete prognostic groups**

R. Leonard, L. Hayward, R. Prescott, N. Allan, S. Das, A. Davison, H. Lucraft, J. MacGillviray, M. Mackie, A. Parker, S. Proctor, G. Ritchie & T. Sarkar

*Scotland and Newcastle Lymphoma Group (SNLG), Edinburgh EH8 9AG, UK.*

463 patients (pts) with working formulation LGNHL, managed with conventional chemo and/or radiotherapy were registered with the SNLG between 1979 and 1987. With a median available follow-up of 4.5 years detailed analysis was performed on pts aged $<70$ years with the aim of identifying poor and good prognostic groups. A model was developed using clinical haematological and pathological presentation data. A test group (118 Edinburgh pts $<70$ years) was excluded from the model and used to confirm its value. Using Cox's proportional hazard model best survival was confirmed to be in females aged 40 years with clinical stage I disease and ECOG performance status (PS) 0. Risk of death was increased by a factor of 5.8 for PS 3/4; by 3.8 for stage III/IV; by 2.4 for stage II; by 2.3 for PS 1/2 and by 1.8 for male sex. The age effect was complex, being worse by 2.16-fold for each decade $>40$ and by 1.63 for each decade $<40$.

Thus using simple multiples of PS, stage, age and sex a patient can be assigned to one of three prognostic categories with very different median and long-term survivals. Tested on the Edinburgh pts the best cohort of 25% have not reached median survival (25% dead at 65 months, actuarial 5-year 83%). The worst cohort of 25% have median survival 37 months, actuarial 5-year 42%. Simple clinical features can be appropriately weighted to provide valuable information for the selection of patients with LG NHL for novel or intensive therapies.

### Recurrence with low grade follicular (LGF) histology in patients previously treated for high grade (HG) non-Hodgkin's lymphoma (NHL)

C.G.A. Price, J. Matthews, A.G. Stansfeld & T.A. Lister

*ICRF, Department of Medical Oncology and Department of Histopathology, St Bartholomew's Hospital, London EC1A, UK.*

A total of 1170 patients have been treated for NHL in this department since 1972. A policy of rebiopsy before retreatment in relapsing patients has been adhered to whenever appropriate. This has enabled the identification of a group of 7 patients in whom LGF histology has been found after successful treatment of HG NHL. Six of these had presented *de novo* with HG disease; they represented 7% of a total of 88 patients who relapsed after achieving remission of primary HG NHL. A single patient changed from HG to LGF histology whose HG disease reflected blastic transformation of previously treated LGF lymphoma.

These patients are important clinically because their prognosis appears to be better than that of the majority of patients relapsing after treatment of HG NHL, and may do well with 'mild' chemotherapy. In this group 5/7 responded to single agent chlorambucil and a further patient responded to an adriamycin containing regimen. 4/7 survive 5 months, 1 year, 2 years and 4 years after diagnosis of LG disease. 3/7 died 9 months, 5 years and 8 years thereafter. They underline the value of rechecking histology at each relapse of NHL.

That LGF disease can persist after elimination of a HG component with intensive chemotherapy reflects the relative 'incurability' of the former histological subtype with conventional treatment. Further investigation of this phenomenon with cytogenetic and molecular techniques may throw light on the mechanisms of histological progression in NHL bearing the 14;18 translocation.

### A comparative study of the nodular and diffuse variants of lymphocyte predominant Hodgkin's disease

A. Borg-Grech, J.A. Radford, D. Crowther, R. Swindell & M. Harris

*Dept of Pathology, CRC Dept of Medical Oncology & Dept of Medical Statistics, Christie Hospital, Manchester, UK.*

Recent evidence has suggested immunophenotypic differences between the nodular and diffuse variants of lymphocyte predominant Hodgkin's disease (HD-LP). To see if these differences are reflected in clinical behaviour we have studied 104 cases of HD-LP treated at this institute. At review, 69 cases had a nodular or mixed (N/M) histological pattern and 35 were diffuse (D). Gender was evenly distributed between the two groups and median age was virtually identical (N/M 39 years, D 38 years). The majority of cases had stage I or II disease (N/M 72%, D 65%), and a peripheral pattern of nodal involvement with 90% of N/M cases and 94% of D cases having cervical and/or axillary adenopathy at presentation. 89% of patients achieved complete remission (CR) and with a median follow up of 89 mths, overall survival (OS) was 80% at 5 years and 75% at 10 years. For those achieving CR, relapse-free survival (RFS) was 88% at 5 years and 78% at 10 years. No difference was observed between the N/M and D subgroups for either OS ($P=0.43$) or RFS ($P=0.40$), results which differ from those of the Stanford Group (Regula *et al.*, *N. Engl. J. Med.*, 1988, **318,** 214), who found that the N and D cases behaved differently, with the former pursuing a chronic relapsing course akin to low grade non-Hodgkin's lymphoma. Moreover, no significant differences in either OS or RFS were observed when the LP cases were compared with 134 cases of nodular sclerosing and mixed cellularity histology matched for stage.

We conclude that the nodular and diffuse variants of HD-LP have a similar natural history and, unlike Regula *et al.*, we have not found histological pattern to be a strong predictor of RFS.

### Extended follow-up of the first UK Wellferon (WFN) study in hairy cell leukaemia (HCL)

A.B.W. Nethersell, P. Bedford, D.A. Jones, M.K. Small, J.M. Bottomley, J. Cawley, D. Catovsky & P. Bevan (for the UK Group)

*Wellcome Research Laboratories, Beckenham BR3 3BS, UK.*

The first 50 consecutive evaluable HCL patients given WFN (3MU daily or thrice weekly according to response and tolerance) have now been followed up for median 122 (31–160) weeks. They received a median WFN dose of 660 (42–1900) MU over a median 67 (2–143) weeks. Overall responses according to international criteria (Golomb *et al.*, *J. Clin. Oncol.*, 1986, **4,** 900) were: CR 8, PR 31, PR (haematological) 7, MR 4. Kaplan–Meier estimates for achieving stated peripheral blood indices *on* therapy and maintaining these *off* therapy are shown in the table.

| | *Probability of achieving or maintaining level* | | | |
|---|---|---|---|---|
| | *On therapy ($n=50$)* | | *Off therapy ($n=39$)* | |
| | $t=0$ | $t=1$ year | $t=0$ | $t=1$ year |
| HC=0 | 16% | 96% | 86% | 29% |
| $Hb \geqslant 12 g dl^{-1}$ | 14% | 92% | 90% | 67% |
| $Pl \geqslant 100+10^9 l^{-1}$ | 50% | 98% | 100% | 91% |
| $N \geqslant 1.5 \times 10^9 l^{-1}$ | 12% | 85% | 89% | 21% |
| $M \geqslant 0.1 \times 10^9 l^{-1}$ | 17% | 88% | 91% | 20% |

The high overall response was followed by clear evidence of progressive relapse off therapy. Reinduction has, to date, always been successful.

Sera from HCL patients in this ($n=23$) and a subsequent ($n=12$) study have been tested for neutralising antibodies by bioassay. The median dose of WFN was 700 (24–2688) MU over a median duration of 56 (1–174) weeks in these 35 patients, none of whom developed neutralising activity.

**PCR used to detect dissemination in gastric lymphoma – implications for treatment**

D. Cunningham, T. Hickish, D. Rosin, P. Sauven, H. Baron, P.J. Farrell & P. Isaacson

*Ludwig Institute for Cancer Research, St Mary's Hospital, London W2; Department of Histopathology, UCH and Middlesex School of Medicine, London W1; St Mary's Hospital, London, UK.*

The polymerase chain reaction (PCR) permits the enzymatic amplification of a target sequence of DNA and by the selection of the appropriate oligonucleotides the method should be sensitive enough to detect at least 1 in $10^5$ cells with the target sequence. In lymphoma, a target sequence for PCR is the rearranged *Bcl*-2 gene which results from a translocation between chromosomes 14 and 18 (t14;18). This translocation is found in over 90% of cases of low grade non-Hodgkin's lymphoma (NHL) and up to 30% of intermediate grade diffuse B-cell NHL. The incidence of the translocation in gastric lymphoma is unknown. The gastrointestinal tract is the most common site of primary extranodal lymphoma and prognosis is most closely related to the stage of disease. The break-point in a patient with a stage I gastric lymphoma was determined by restriction enzyme digestion. Based on this localisation, oligonucleotide primers were prepared which would permit PCR across break point. Using PCR, malignant lymphoma cells with the *Bcl*-2 gene rearrangement were found in the peritoneal washings taken 2 weeks after surgery and bone marrow of a patient who had an apparently localised gastric lymphoma. Bone marrow and peritoneal washings were cytologically normal. Following four courses of cytotoxic drug therapy the cells could no longer be demonstrated in either site.

This is the first time PCR has been used to demonstrate malignant cells in peritoneal washings. PCR is a useful addition to the staging investigations of non-Hodgkin's lymphoma and can also be used to monitor response to treatment.

**Use of the polymerase chain reaction (PCR) in the diagnosis of extra-nodal progression of follicular non-Hodgkin's lymphoma (*F*-NHL)**

C.G.A. Price, B.D. Young & T.A. Lister

*ICRF Department of Medical Oncology, St Bartholomew's Hospital, London EC1, UK.*

The technique of amplification of short segments of DNA by means of the PCR is rapidly finding applications in the investigation of genetic, neoplastic and viral diseases. In F-NHL, which is associated with the 14;18 translocation in the majority of cases, amplification of the 14;18 breakpoint region can be used to demonstrate occult tumour cells within pathological samples. A direct clinical application of this is in the rapid diagnosis of F-NHL in situations where material for morphological examination is unavailable or difficult to interpret. Two illustrative cases are described. (1) A woman of 48 with a 14 year history of recurrent F-NHL presented with a pleural effusion. Pleural biopsy and cytology were non-diagnostic. DNA from pleural mononuclear cells was amplified using Taq polymerase and primers from $J_H$ region of chromosome 14 and *Bcl*-2 gene on chromosome 18. A single amplified product was identified by electrophoresis; that this was the 14;18 breakpoint region was confirmed by probing with a second $J_H$ region oligonucleotide. (2) A man of 65 with persistent F-NHL confined to the bone marrow developed raised red truncal skin lesions. Immunophenotyping of skin biopsy showed aggregates of B1 positive cells, but a definite morphological diagnosis of F-NHL was not possible. PCR amplification of DNA was once again positive; different primers were required in this case, indicating that the breakpoint occurred at a different site in the major breakpoint region (mbr) of the *Bcl*-2 gene. The rapidity of this technique (e.g. in comparison with gene rearrangement studies), its sensitivity and the specific genetic information obtained suggest that it will be of value in the investigation of occult F-NHL.

**A successful treatment for chemotherapy induced anticipatory nausea and vomiting**

M.A. Ratcliffe, L.G. Walker, A.A. Dawson, S.M. Pollet, L. Hamilton & J. Lolley

*Dept of Medicine and Dept of Mental Health, Royal Infirmary, Aberdeen AB9 2ZB, UK.*

In a previous retrospective study in Aberdeen of 84 patients with lymphoma or teratoma the incidence of conditioned nausea and/or vomiting was 41%.

Eighteen patients having cytotoxic chemotherapy who had, despite anti-emetics, developed severe anticipatory symptoms, particularly nausea and vomiting, were treated by nausea management training (NMT) during hypnotherapy and relaxation sessions. This training, a form of behavioural psychotherapy, involved helping patients to experience nausea by appropriate imagery, then using direct suggestion and gentle abdominal self massage to eliminate the nausea. Symptoms were assessed sytematically by the physician at the time of referral for NMT and at completion of chemotherapy and in all patients were found to have improved.

A prospective study was initiated with 69 newly diagnosed lymphoma or teratoma patients about to have cytotoxic chemotherapy. Using an 8 point scale each patient graded the severity of their anticipatory and pharmacological symptoms after each pulse of treatment. Following the third pulse patients were randomly allocated to three sessions of NMT with hypnotherapy or relaxation, or to a control group. Results recorded a low incidence of anticipatory symptoms, at 14%, and there was poor compliance with NMT. Prophylactic NMT was thus not evaluable. It is possible that the intervention in undertaking the study influenced the incidence of conditioning.

However, for these patients with anticipatory problems hypnotherapy or relaxation with NMT has been proved to be of benefit.

**Efficacy and safety of three 6-hourly i.v. doses of 40 $\mu$g kg$^{-1}$ of granisetron in cytotoxic induced emesis**

V. Raina, J. Cassidy, S. Kaye, M. Soukop & S. Thompson

*Depts of Medical Oncology, Royal Infirmary/Western Infirmary, Glasgow; Beecham Pharmaceuticals Ltd, Research Division, Essex, UK.*

Previous studies on granisetron, a new selective 5HT3 antagonist, have demonstrated significant efficacy of a single i.v. dose of 40 $\mu$g kg$^{-1}$ for cytotoxic (including cisplatin) induced emesis (Cassidy *et al.*, *Br. J. Cancer*, 1988, **58**, 651; Carmichael *et al.*, *BMJ*, 1988, **297**, 110). The optimum dose and scheduling of granisetron has not, however, been established. This pilot study was undertaken to assess the efficacy and safety of 3 doses of 40 $\mu$g kg$^{-1}$ granisetron given i.v. at 6 hourly intervals, each by 30 min infusion. Chemotherapy was started at the end of the first infusion. 23 of 25 patients who had a variety of intermediate to severe emetogenic drugs (6 platinum) completed treatment. Two patients developed rigors and pyrexia after the first dose of granisetron and were withdrawn. Nine patients were naive to chemotherapy. Subjective and objective assessments of nausea, vomiting and symptoms were done 6 hourly for the first 24 hours after the start of granisetron treatment. Subjective assessment of efficacy continued for up to 6 more days. Using subjective rating scales, 14 (1 cisplatin) out of 23 assessable patients recorded no vomiting during the first 24 hours. This was confirmed by objective assessment of vomiting incidence. Of 19 patients assessable for nausea during the same period, no nausea was seen in 9 (1 cisplatin), mild in 6 (2 cisplatin), moderate in 2 (2 cisplatin) and severe in 2 (0 cisplatin). Only 3 patients required a rescue antiemetic in the first 24 hours. In a retrospective open comparison made after the next chemotherapy session under conventional antiemetic cover, 10 from 20 assessable patients (50%) (4 cisplatin) preferred granisetron, 3 (1 cisplatin) had no preference and 7 (35%) (1 cisplatin) preferred a conventional antiemetic. This study establishes the effectiveness of a total of 120 $\mu g\ kg^{-1}$ granisetron given in 3 divided doses in the first 24 hours in cytotoxic induced emesis. Due to the variation in time of onset of emesis with different cytotoxics, the advantages of repeated dosing over single doses of granisetron were not clear. One of the 2 patients who had rigors had an infected Hickman line and the cause of the untoward reaction in the other patient was not clear. The safety of repeated dosing in 24 hours was also demonstrated.

**A randomised trial of aminoglutethimide and hydrocortisone ($AG^+$ HC)$^\pm$ aminohydroxypropylidene diphosphonate (APD) in patients with advanced breast cancer**

B. Cantwell, K.A. Mannix, J. Carmichael & A.L. Harris

*University Dept of Clinical Oncology, Newcastle General Hospital, Newcastle-upon-Tyne NE4 6BE, UK.*

Diphosphonates can induce healing of bone metastases in breast cancer, with sclerosis of lytic bone metastases. To investigate the value of APD with a standard hormone therapy in breast cancer we have performed a randomised trial of AG+HC ± APD in post-menopausal women with breast cancer and skeletal metastases. All patients received AG 125 mg bd and HC 20 mg bd continuously. Those randomised to receive APD in addition had i.v. infusion of APD 30 mg at 21 day intervals to a total of 12 infusions (i.e. to 36 weeks). Assessment included serial clinical, scan, X-ray and biochemical evaluations. Of the first 25 women randomised, 5 had rapid disease progression and early death and 1 patient was withdrawn because of AG intolerance. Of the 19 remaining patients, 10 received APD in addition to AG/HC. Radiological assessment of bone metastases showed objective response in 4 patients (40%) in the APD group and 3 (33%) in the AG/HC alone group after 12 weeks' treatment. At 24 weeks, 3 (30%) APD patients and 2 (22%) AG/HC alone patients maintained this response. At 36 weeks the response was continued among APD patients (30%) but there were no continuing responders among the AG/HC alone group. After 1 year, 3 (30%) APD patients have continuing response in bone. The response in non-skeletal sites of disease in patients receiving APD compared with AG/HC alone was 40% and 22% respectively at 36 weeks and 30% and 0 at 1 year. Preliminary analysis suggests that short out-patient infusions of APD 30 mg contributes to the efficacy of low dose AG+HC for patients with advanced breast cancer and predominantly bone metastases. This effect may be an enhancement of duration rather than rate of response in the combined modality treatment group.

**APD for the treatment of hypercalcaemia of malignancy (HOM): a comparison of different doses and schedules of administration**

R.E. Coleman & R.D. Rubens

*ICRF Clinical Oncology Unit, Guy's Hospital, London SE1 9RT, UK.*

APD is an effective treatment for HOM but the optimum dose and schedule have not been defined. We have treated 43 patients with hypercalcaemia secondary to advanced breast cancer with APD. Patients were hydrated with 4–6 litres of intravenous saline over 36–48 hours prior to treatment with APD. 21 received a single 30 mg dose (study A) but were randomised between a short (2 hours, 11 patients) and long (24 hours, 10 patients) infusion time. 23 patients received a single 15 mg dose over 2 hours (study B). Additional doses of APD were permissible if the serum calcium had not improved at 48 hours.

Thirty-seven patients (84%) achieved normocalcaemia with a nadir in serum calcium and urinary calcium excretion at 5–6 days. Two patients died within 24 hours of APD (1 lymphangitis, 1 myocardial infarction). Five patients remained hypercalcaemic (1 study A, 4 study B). The time to control hypercalcaemia and duration of normocalcaemia were similar in both studies and uninfluenced by the duration of APD infusion. The nadirs in serum calcium were lower in study A but were identical for urinary calcium excretion suggesting equivalent inhibition of bone resorption. Three patients in study B required a second 15 mg dose of APD to control the serum calcium. Additional doses were not given in study A.

These results suggest that, in breast cancer, there is no clear evidence of either a dose–response relationship, between 15 and 30 mg, or of improved efficacy with a long schedule of administration.

**Primary medical therapy in operable breast cancer**

J.L. Mansi, I.E. Smith, G. Walsh, H.D. Sinnett & J.A. McKinna

*The Breast Unit, Royal Marsden Hospital, Fulham Road, London SW3 6JJ, UK.*

Fifty-seven patients with large but potentially operable primary breast cancer were treated with chemotherapy (15) or endocrine therapy (42) with the tumour remaining *in situ*, and with the aim of avoiding mastectomy. For patients treated with chemotherapy, 1 (7%) achieved a complete remission and 8 (53%) a partial response (overall response 60%). The rest had either a minor response (2) or stable disease (3), and one progressed on chemotherapy. For patients who received endocrine therapy one (2%) achieved a complete response, and 19 (45%) a partial response. Four (10%) patients had a minor response, 17 (38%) no change and 2 (4%) progressed whilst on therapy. Only 10 patients have so far had a subsequent mastectomy (18%), and 17 (30%) have had radiotherapy and/or conservative surgery. The rest are still on medical therapy.

With a median follow-up of 19 months (range 6–42) no patient has developed uncontrollable local disease and only 2 (4%) have had a local recurrence after being disease-free. Eight (14%) patients have developed distant metastases and 4 (7%) have died of metastatic disease.

Primary medical therapy followed by radiotherapy may offer an effective alternative to mastectomy for the majority of patients with breast carcinomas inappropriate for conservative surgery and this approach merits further study.

**Identification of patients with poor prognosis stage II breast cancer**

S.M. O'Reilly, R.S. Camplejohn, A. Howell, R.D. Rubens & M.A. Richards

*ICRF Clinical Oncology Unit, Guy's Hospital, Richard Dimbleby Dept of Cancer Research, St Thomas' Hospital, London SE1; and Christie Hospital, Manchester, UK.*

Adjuvant chemotherapy improves the 5 year survival of patients with breast cancer by approximately 3.5% (*N. Engl. J. Med.*, 1988, **319,** 1681). Intensification of treatment might improve the outcome for some subgroups, but carries the risk of early treatment related mortality. Such treatment might be justified for patients ages ≤60 years at high risk of early distant metastases. The outcome for 341 patients aged ≤60 years in the Guy's/Manchester adjuvant CMF study was reviewed in order to identify a subgroup with a 2 year distant metastasis free survival (DMFS) <20%. DNA flow cytometry was performed on tissue from Guy's patients with ≥10 positive nodes.

In premenopausal control patients, the 2 year DMFS of patients with ≥7 positive nodes was <20%. Further analysis based on age, tumour size, histological grade or steroid receptor status identified no similar group in patients with 1–6 positive nodes. Among postmenopausal patients aged ≤60 years, only the combination of ≥10 positive nodes and an S phase fraction > the median selected a similar group. The 5 year survival of the combined pre- and postmenopausal poor prognosis group (29 patients) was <20%.

Thirty-one patients with the same 'poor prognosis' presentation features received CMF. When compared with their controls, a significant improvement in both DMFS ($P<0.001$) and survival ($P<0.001$) was seen, but the actuarial curves show little, if any, evidence that this treatment is curative.

This study indicates that 60/341 (17%) of patients aged ≤60 years with stage II breast cancer may be suitable candidates for intensification of adjuvant treatment.

**Two weekly high-dose doxorubicin therapy with infusions of granulocyte colony-stimulating factor in patients with advanced breast and ovarian cancer**

M.H. Bronchud, A. Howell, D. Crowther, P. Hopwood, L. Souza & T.M. Dexter

*Christie Hosptial, Manchester, UK.*

Granulocyte colony stimulating factor (G-CSF) was given to 17 patients with advanced breast and ovarian cancer in order to increase the intensity and effectiveness of chemotherapy. Treatment with doxorubicin, at doses of 75 mg m$^{-2}$ ($n=4$ patients), 100 mg m$^{-2}$ ($n=5$), 125 mg m$^{-2}$ ($n=6$) and 150 mg m$^{-2}$ ($n=2$), was followed by infusion of G-CSF for 11 days. G-CSF administration resulted in a return of the absolute neutrophil count to normal and above normal levels within 12–14 days at all dose levels of doxorubicin used and allowed the administration of up to three cycles of high dose chemotherapy at 14 day intervals. An absolute neutrophil count $>2.5\times10^9$ l$^{-1}$ was not reached until days 19–21 after 75 mg m$^{-2}$ of doxorubicin given without G-CSF. At doses of doxorubicin of 125 mg m$^{-2}$ and 150 mg m$^{-2}$ all tumours regressed rapidly and the response rate was significantly higher that at the lower doses ($P<0.036$), although there was marked epithelial toxicity. Two months after doxorubicin G-CSF therapy there was a pronounced improvement of symptoms compared with before treatment. Thus the effectiveness of chemotherapy may be enhanced and treatment duration shortened by the use of G-CSF infusions. Further studies of this promising approach are warranted.

**High-dose melphalan with granulocyte-macrophage colony-stimulating factor (GM-CSF) in the treatment of metastatic colorectal carcinoma**

W.P. Steward, J.H. Scarffe, K. Borkett, E. Bonnem & D. Crowther

*Dept of Medical Oncology, Christie Hospital, Manchester, UK; and Schering Corporation.*

Nine patients (pts) with metastatic disease from primary colorectal carcinoma were entered into a phase I/II study using continuous intravenous (i.v.) infusions of GM-CSF and high-dose melphalan (120 mg m$^{-2}$). GM-CSF was given alone during the phase I part of the study to determine a dose that would produce a leucocyte count (WBC) $\geq50\times10^9$ l$^{-1}$ and was initially given at 3 μg kg$^{-1}$ day$^{-1}$ and escalated to 10 μg kg$^{-1}$ day$^{-1}$ after 10 days. The infusion was discontinued at WBC$\geq50\times10^9$ l$^{-1}$ and 1 week later melphalan was given i.v. over 30 minutes. GM-CSF was recommenced 8 hours later at the endpoint dose determined from the phase I part of the study and was continued until the neutrophil count recovered to $\geq0.5\times10^9$ l$^{-1}$ for 7 consecutive days.

One patient achieved a WBC$\geq50\times10^9$ l$^{-1}$ with GM-CSF at 3 μg kg$^{-1}$ day$^{-1}$ in the phase I part of the study but the other pts required 10 μg kg$^{-1}$ day$^{-1}$. No toxicity attributable to GM-CSF was seen. After melphalan, the median times to neutrophil and platelet counts $<500\times10^9$ l$^{-1}$ and $<20\times10^9$ l$^{-1}$ respectively were 6 and 8 days. The median durations of neutropenia ($<500\times10^9$ l$^{-1}$) and thrombocytopenia ($<20\times10^9$ l$^{-1}$) were 15 and 10 days respectively. All pts required intensive support with a median duration inpatient stay of 28 days. There was 1 treatment related death due to renal failure. One complete response and 2 partial remissions (33% response rate) were seen but these were of short duration (median 2 months).

This study suggests that GM-CSF given by continuous i.v. infusion is associated with no toxicity and produces significant WBC increments at a dose of 10 μg kg$^{-1}$ day$^{-1}$. The duration of neutropenia and thrombocytopenia induced by high-dose melphalan appear to be reduced by subsequent administration of GM-CSF when the results of this study are compared with the published literature.

**A pilot study of high dose busulphan with autologous marrow rescue in myeloma**

M. Harding, C. Viner, I. Judson, M. Gore, B. Millar & T. McElwain

*Institute of Cancer Research, Royal Marsden Hospital, Sutton, Surrey, UK.*

We have initiated a pilot study of high dose busulphan with autologous marrow rescue in myeloma, as *in vitro* data suggest that lymphoplasmacytoid clonogenic cells are more sensitive to busulphan than melphalan. To date, 7 pts have been treated (median age 52 years, range 37–59). All had previously responded to VAMP (vincristine 0.4 mg+adriamycin 9 mg m$^{-2}$ infused over 24 hours for 4 days+methylprednisolone 1.5 g orally days 1–5: q 21 days) with high dose melphalan (200 mg m$^{-2}$) in 6 patients. At the time of treatment 5 had disease refractory to VAMP+cyclophosphamide, the other 2 had relapsed after partial remissions of 2 and 4 months. Busulphan 1 mg kg$^{-1}$ was given orally 6 hourly for 4 days with continuous intravenous sodium bicarbonate and prophylactic phenytoin. Cryopreserved bone marrow was returned on day 7. The median duration of WHO grade IV leucopenia and thrombocytopenia was 17 days (ranges 10–30 and 4–70 days respectively); 1 pt died from intracranial haemorrhage on day 26. Oropharyngeal mucositis (grade II–IV) occurred in all pts and CNS toxicity was common (1 grand mal convulsion, 3 severe motor restlessness and agitation).

Three pts had transient (<2 month) reduction in paraprotein and 1 had symptomatic improvement. Two have partial remissions (PR: reduction in paraprotein to <50%, clearing of Bence Jones proteinuria) and 1 had a late paraprotein fall, insufficient as yet to qualify as PR. The preliminary results are encouraging and accrual continues.

**Treatment of advanced ovarian cancer using high-dose melphalan with autologous bone marrow transplantation**

R.J. Osborne, P.I. Clark, C. Price, M.L. Slevin & T.J. McElwain

*Imperial Cancer Research Fund Department of Medical Oncology, Homerton and St Bartholomew's Hospitals, London; and Royal Marsden Hospital, Sutton, UK.*

For patients with ovarian cancer which is recurrent after or refractory to first line chemotherapy, the outlook with conventional chemotherapy is bleak. Accordingly we have investigated the activity of high-dose chemotherapy with melphalan, supported with autologous bone marrow transplantation, in advanced ovarian cancer. Ten patients were treated, with a mean age of 47.5 years (35–54) and median Karnovsky status 80% (50–90%). One patient had stage III disease and 9 stage IV. All patients had bulky (>2 cm) disease. Four patients had been previously treated with high-dose cyclophosphamide and cisplatin, 3 with carboplatin, 1 with platinum, adriamycin and cyclophosphamide and 2 patients had had no prior treatment.

Priming with 300 mg $m^{-2}$ cyclophosphamide was followed 1 week later by intravenous high-dose melphalan 200 mg $m^{-2}$. Bone marrow, harvested immediately before high-dose melphalan, was refrigerated and then reinfused 8 hours after treatment.

Five of 10 patients (1 previously untreated) responded to treatment (1 clinical CR, 4PR). Median duration of remission was 4 months (4–11 months). Median survival after high-dose melphalan was 8.5 months (0.5–26 months). Two patients died within 1 month of treatment from infection. Haematological toxicity was severe. Patients had a mean time of 18.8 days (12–41) with WCC $<1\times10^9$ $l^{-1}$ and 23.5 days (16–61) with platelets $<50\times10^9$ $l^{-1}$. Nine patients developed pyrexia >38.5°C requiring intravenous antibiotics.

Although high-dose melphalan with autologous bone marrow transplantation achieves useful remissions in some patients in this poor prognosis group, the short duration of remission and marked toxicity renders it unsuitable for general use in previously treated patients.

**Randomised trial of oral verapamil with chemotherapy for non-small cell lung cancer (NSCLC)**

M.J. Millward, P.A. Corris, A.L. Harris & B.M.J. Cantwell

*University Dept. of Clinical Oncology, Newcastle General Hospital, Newcastle-upon-Tyne NE4 6BE; and Cardiothoracic Centre, Freeman Hospital, Newcastle-upon-Tyne NE7 7DN, UK.*

The calcium channel blocker Verapamil can reverse multidrug resistance (MDR) *in vitro*. To test the clinical value of this concept we have treated 37 patients with locally advanced or metastatic NSCLC who were chemotherapy naive with Ifosfamide/Mesna 5 g $m^{-2}$ + Vindesine 7 mg total and either oral verapamil 160 mg t.d.s. for 72 hours commencing 24 hours prior to chemotherapy or no additional treatment. Vindesine was given by bolus intravenous (i.v.) injection and Ifosfamide by 24 hour i.v. infusion with concomitant Mesna. Courses were repeated every 3 weeks to a maximum of 6. The response rate is 31% in the verapamil arm and 22% in the no-verapamil arm (n.s.) and the median survival 41 weeks versus 27 weeks. There was no significant difference in survival between those who received verapamil and those that did not (log-rank test $P=0.14$). In patients with metastases or local recurrence ($n=13$) there was a trend toward better survival in patients who received verapamil (log-rank test $P=0.06$). There was one probable treatment related death (infection without myelosuppression). WHO grade 3 or 4 myelosuppression occurred in 4 patients with one episode of sepsis. Reversible Ifosfamide encephalopathy occurred in 4 patients. One patient who received verapamil developed grade 4 ileus, and asymptomatic first degree heart block was noted in one patient receiving verapamil. The addition of verapamil to chemotherapy in NSCLC does not appear to improve response rates suggesting that verapamil, in the doses given orally, does not circumvent MDR. However, a possible positive effect on survival in patients with metastatic disease, suggests that verapamil may have other effects in NSCLC. Accrual of patients continues.

**Intermittent high dose tamoxifen (HDT) with oral etoposide (EPO): phase I and II clinical studies**

B. Cantwell, J. Carmichael, M.J. Millward, M. Chatterjee & A.L. Harris

*University Dept of Clinical Oncology, Newcastle General Hospital, Newcastle-upon-Tyne NE4 6BE, UK.*

To circumvent multidrug resistance (MDR) in malignant tumours, patients (pts) with progressive cancers poorly responsive or resistant to conventional therapies were treated with HDT 120 mg daily plus EPO 300 mg daily, both drugs for 3 days, repeated at 21 day intervals. Of 18 pts so treated 1 pt with mesothelioma had a partial response (PR) and 3 others (2 with lung cancer and 1 with soft tissue sarcoma) had stable disease (SD) >3 months. Broad phase II evaluation has occurred in >60 pts with HDT 320 mg daily for 6 days plus EPO 300 mg daily on days 4, 5 and 6 only, and cycles were repeated at 21 day intervals. Of 46 pts so far evaluable for response there were 1 complete response (CR) and 1 SD in 2 pts with gastric cancer, 1 CR, 1 PR and 1 SD in 6 pts with soft tissue sarcoma, 1 PR in 3 pts with non-small cell lung cancer, 1 PR in 3 pts with melanoma and 1 PR and 3 SD in 7 pts with platinum analogue resistant ovary cancer. Six other patients had SD and 29 pts had PD. Toxicity overall was mild with WHO grade 3 or 4 myelosuppression in only 4 pts, but alopecia was common. There were 2 pts with marked visual disturbances and 3 with thromboembolism, possibly therapy related. 2 pts discontinued because of tamoxifen related emesis, and there was 1 possible allergic event. Parallel laboratory experiments using tamoxifen to reverse resistance in MDR Chinese Hamster ovary cell mutants and estimation of plasma tamoxifen levels in patients have been performed and these laboratory correlates will be presented. Continued tumour specific phase II and also randomised studies are indicated in soft tissue sarcoma, melanoma and gastric, non-small cell lung and ovary cancers.

### A pilot study of epirubicin and quinidine in advanced breast cancer

R.D. Jones, E.M. Rankin, T. Habeshaw, A.N. Harnett, S.B. Kaye, D.J. Kerr, J. Plumb, S. Rae, R. Rampling, N.S. Reed & S. Stallard

*Beatson Oncology Centre, Western Infirmary, Glasgow, UK.*

Tumour cell resistance to anthracyclines limits the effectiveness of these drugs in breast cancer. Quinidine at a concentration of 6.6 μmol is capable of increasing by 10-fold the sensitivity of the multidrug resistant breast cancer cell line MCF-7 to adriamycin.

Twenty-five patients have entered a feasibility study combining oral quinidine with epirubicin 100 mg $m^{-2}$ as first line chemotherapy for advanced breast cancer. A maximun of 8 cycles were given, the first without quinidine. To achieve steady state levels quinidine durules were given 4 days prior and continued 1 day following epirubicin administration. Three patients received quinidine at a dose of 1 g. b.d.:2 developed symptoms of cinchonism and 1 nausea and vomiting. Of 8 patients treated with 500 mg b.d., 2 experienced tiredness and nausea and 1 developed severe oral toxicity with epirubicin. Quinidine 250 mg b.d. has been well tolerated by 13 patients. Epirubicin induced toxicity was not significantly increased. Mean nadir WBC with epirubicin was $1.5 \times 10^9$ $l^{-1}$ (range 1.1–2.5) and with epirubicin/quinidine was $1.5 \times 10^9$ $l^{-1}$ (range 1.0–2.7). There was no evidence of significant cardiac toxicity as judged by 12 lead ECG, 24 hour ambulatory monitoring and echocardiography. Plasma quinidine levels were assayed at the time of epirubicin infusion and the mean value was 7.4 μmol $l^{-1}$ (range 3.5–14).

We conclude that at a dose of 150 mg b.d. quinidine plasma levels similar to those active *in vitro* can be achieved with minimal toxicity. A prospective randomised trial of epirubicin *vs* epirubicin and quinidine in advanced breast cancer will commence shortly.

### The analgesic activity and pharmacokinetics of morphine-6-glucuronide in man

R.J. Osborne, M.L. Slevin, S.P. Joel, D.M. Trew, P.I. Thompson & J.S. Malpas

*Imperial Cancer Research Fund Department of Medical Oncology, St Bartholomew's and Homerton Hospitals, London EC1A 7BE, UK.*

Morphine-6-glucuronide (M6G), a natural morphine (M) metabolite, posesses analgesic activity in animals, its potency exceeding that of M. We have investigated the clinical activity and pharmacokinetics (PK) of synthetic M6G in man to determine the likely contribution of this abundant metabolite to the analgesia occurring after conventional M treatment. Synthetic M6G (1 mg $ml^{-1}$ in 0.9% saline) was administered intravenously (i.v.) at a dose of 1 mg $70kg^{-1}$ to 10 cancer patients with moderate to severe pain. Pain relief, defined as a sustained improvement of 2 points or more on a 4 point pain scale (none = 0, mild = 1, moderate = 2, severe = 3) was assessed. Cardiorespiratory function and subjective symptoms were monitored. 8/9 assessable patients experienced pain relief, the median duration being 6 hours (range 1–18 hours). There were no clinically significant cardiorespiratory effects or other adverse effects. Plasma M6G PK were examined using an HPLC method (*J. Chrom.*, 1988, **430**, 394) in 6 patients.

| | *Normal (n = 4)* | *Renal impairment (n = 2)* |
|---|---|---|
| Creat. cl. ($ml\,min^{-1}\,70\,kg^{-1}$) | 76 ± 15 | 24 ± 9 |
| M6G cl. ($ml\,min^{-1}\,70\,kg^{-1}$) | 99 ± 11 | 31 ± 8 |
| M6G $AUC_{0.00}$ ($nmol^{-1}\,h^{-1}\,70\,kg^{-1}$) | 346 ± 40 | 1,106 ± 301 |

Renal dysfunction impaired M6G elimination. No M or M3G were identified in plasma or urine. The M6G $AUC_{0.00}$ after 1 mg 70 $kg^{-1}$ i.v. M6G was similar to that previously observed after i.v. or oral M treatment at a dose 10 mg 70 $kg^{-1}$ (*Proc. ASCO*, 1987, **6**, 1077). Six further patients administered M6G at a dose of 2 mg 70 $kg^{-1}$ intravenously also experienced pain relief. None of the patients studied at the higher dose experienced any clinically significant cardiorespiratory effects or other adverse effects. These findings clearly indicate that M6G is a narcotic agonist in man, and strongly suggest that the metabolite contributes considerably to the clinical effects seen after M treatment.

### The effect of age and obesity on the pharmacokinetics of ifosfamide

M.J. Lind, J.M. Margison, T. Cerny, N. Thatcher & P.M. Wilkinson

*CRC Dept of Medical Oncology; Dept of Clinical Pharmacology, Christie Hospital and Holt Radium Institute, Manchester, M20 9BX, UK.*

The pharmacokinetics single agent intravenous ifosfamide in patients with carcinoma of the bronchus were studied to see if they correlated with age or obesity. A positive correlation between age and half life ($t_{1/2\beta}$) was demonstrated ($r = 0.48$, $0.01 < P < 0.05$). The increase in elimination half life was attributed to an increase in the volume of distribution of ifosfamide (Vdβ) with age ($r = 0.48$, $0.01 < P < 0.05$). Correspondingly total clearance and non-renal clearance of ifosfamide did not alter with age. In obese patients the terminal elimination half life ($t_{1/2\beta}$) was found to be higher than in the control group (6.36 hours (range 5.77–7.45) *vs* 4.95 hours (range 1.82–6.48), $P < 0.05$). The volume of distribution (Vdβ) in the obese group was 42.81 (range 35.49–51.90) *vs* 33.70 (range 17.76–50.62), $P < 0.05$. There was no significant difference in total plasma clearance between the obese and normal groups. The volume of distribution (Vdβ) correlated with both total body weight and percentage of ideal body weight but not with ideal body weight. When Vdβ was normalised for ideal body weight there was a strong positive correlation with percentage ideal body weight, suggesting that ifosfamide will distribute into the total body weight in excess of the ideal body weight.

### Carboplatin: what is the right dose?

E.M. Rankin & J. Paul

*CRC Department of Medical Oncology, Beatson Oncology Centre, Western Infirmary, for Scottish Ovarian Cancer Study Group.*

The dose limiting toxicity of carboplatin (JM8) is myelosuppression. We have compared JM8 400 mg $m^{-2}$ i.v. alone (arm A) with a combination of JM8 300 mg $m^{-2}$ and chlorambucil 10 mg daily × 7 days (arm B) every 28 days for 6 courses as first line therapy for ovarian cancer. The dose for each course was adjusted according to the nadir count of the previous course. If the nadir white count $> 4 \times 10^9$ $l^{-1}$ *and* platelets $> 120 \times 10^9$ $l^{-1}$ dose of JM8 was increased to

500 mg m$^{-2}$ arm A, and 375 mg m$^{-2}$ in arm B. If the nadir white cell count $<1\times10^9$ l$^{-1}$ *or* the platelets $<25\times10^9$ l$^{-1}$ JM8 was reduced to 300 mg m$^{-2}$ in arm A and 225 mg m$^{-2}$ in arm B and chlorambucil to 10 mg day$^{-1}$ for 5 days for all subsequent courses. The dose received for the third course of therapy (when the majority of dose adjustments had been made) was compared with that calculated according to the various formulae for JM8 which depend upon renal function and pretreatment platelet count (Egorin *et al*., *Cancer Res*., 1985, **45**, 6502; Fish *et al*., *Proc. ECCO*, 1987, 215) or renal function and AUC (Calvert *et al*., *Proc. ASCO*, 1987, 645). We found substantial differences between dose given according to our protocol and that indicated by the available formulae. Up to 60% of patients received more than the indicated dose; 41% arm A and 20% arm B received >100 mg more than indicated dose. Preceding myelosuppression led to dose reduction, 34% arm A and 20% arm B received >100 mg less than indicated dose. Our data show there is considerable inter-patient variability in the handling of JM8. Provision for dose escalation as well as reduction allows maximum safe dosing of JM8 alone or in combination. If tumour response correlates with dose intensity of JM8 (as it does for cisplatin) then it will be important to optimise dose in the individual patient.

**Pharmacokinetics of repeated antibody therapy**

R.H.J. Begent, J.A. Ledermann, A.J. Green, S.J. Riggs, S.E. Dewhurst, A. Kelly, D.B. Smith, C. Massof, M.G. Glaser, R.G. Dale & K.D. Bagshawe

*Cancer Research Campaign Laboratories, Charing Cross and Westminster Medical School, London W6 8RF, UK.*

Antibody targeted therapy of cancer is limited to one or two repeated doses because of production of human antibody directed against xenogeneic antibody or the conjugated therapeutic agent. Cyclosporin A has been shown to prevent or delay anti-antibody production and permit repeated therapy (Ledermann *et al*., *Br. J. Cancer*, 1988, **58**, 654). The purpose of this study was to investigate the distribution of radiolabelled antitumour antibody in tumour and normal tissues with repeated therapy. Six patients with carcinoembryonic antigen (CEA) producting tumours received 2–4 (mean 3.3) doses each of 5–7.5 mg mouse monoclonal antibody to CEA (A5B7) labelled with approximately 50 mCi iodine 131. Cyclosporin A 25 mg kg$^{-1}$ day$^{-1}$ by mouth for 6 days with each dose of antibody was given to 3 patients, the remainder had 15 mg kg$^{-1}$ day$^{-1}$ continuously. Human antimouse antibody production was suppressed by these regimens. Serial estimations of radioactivity were made in tumour, blood, liver and lung by gamma camera imaging with single photon emission tomography (Riggs *et al*., *Int. J. Cancer*, 1988, Suppl. 2, 95). The percentage of injected activity in tumour and normal tissues showed no significant difference between the first and subsequent doses of antibody. The rates of clearance from tumour and normal tissues did not vary significantly with each repeated dose. The results suggest that the effect of antibody targeted therapy can be augmented by repeated therapy provided that anti-antibody production is prevented.

**Use of CA125 to predict survival of patients with ovarian carcinoma**

G.J.S. Rustin, J.N. Gennings, A.E. Nelstrop, H. Covarrubias, H.E. Lambert & K.D. Bagshawe

*CRC Lab., Dept of Medical Oncology, Charing Cross Hospital, London W6 8RF; and Dept of RTOP & Oncology, Hammersmith Hospital & Mount Vernon Hospital, London, UK.*

The prognostic value of serum CA125 measurements was assessed in 54 patients with advanced ovarian adenocarcinoma. They all received a minimum of two courses of carboplatin as part of the North Thames Cooperative Group Trial. With a minimum follow up 14 months, 37 (69%) have clinical evidence of progressive disease. The absolute prechemotherapy level of CA125 was of no value in predicting those patients who have developed progressive disease. However, the change in CA125 levels from immediately prior to chemotherapy to one month later, after one course of carboplatin could be used to divide patients into different prognostic groups. The best discrimination was found by dividing the patients into those who showed a greater than 7-fold decrease in CA125 levels and those who showed a smaller change. Eight of 14 (58%) patients with a greater than 7-fold decrease in CA125 levels remain disease free compared to 3 of 36 (9%) patients with a lesser fall ($P=0.0005$). The change in CA125 levels during the first month of chemotherapy may indicate which patients should be offered alternative or symptomatic therapy and which patients should continue with the currently available toxic chemotherapy.

**The use of early serum CA125 response in the management of epithelial ovarian cancer**

C.W.E. Redman, G. Blackledge, E.J. Buxton, D.M. Luesley & K.K. Chan

*West Midlands CRC Clinical Trials Unit, Queen Elizabeth Medical Centre, Birmingham B15 2TH, UK.*

Despite the advent of active chemotherapy in epithelial ovarian cancer (EOC), not all patients respond to treatment and in some responding patients the duration of response is short-lived, failing to justify the toxicity and disruption in the quality of life that such treatment can entail. The monitoring and assessment of response in EOC can be difficult, but serum CA125 may facilitate an early appraisal of response and identify those patients unlikely to derive benefit from continued intensive chemotherapy. Serum CA125 levels were measured in 50 EOC patients, receiving first-line cisplatinum based chemotherapy, prior to treatment and after 2 cycles of treatment. Subject to response (assessed by UICC criteria) and toxicity patients received up to 8 courses of treatment. The overall response rate was 76% and median survival for the group was 16.4 (95% CI=9.8–23.0) months. Median time on-study was 25 (95% CI=23–25) months; range=14–32 months. An initial univariate analysis of prognostic variables, including serum CA125 levels before treatment and after 2 courses, and % fall in serum CA125 levels was performed. In this analysis a cut-off point of 30 U ml$^{-1}$ for CA125 values, and multiple cut-off points (25–90) for % fall were used. On the basis of this analysis, residual disease, and serum CA125, both prior to treatment and after 2 courses, were significant prognostic variables, of which the second serum CA125 value was the most important. Patients with a second serum CA125<30 U ml$^{-1}$ were more likely to achieve a complete remission ($\chi_1^2=17.8$; $P<0.0001$) and survived significantly longer ($\chi_1^2=11.1$; $P=0.0009$) than patients with elevated levels. Because of the potential interrelation of these and other factors, a stepwise discriminant analysis was performed using a survival at 12 months after primary surgery as the end point. Serum CA125 ($\log_e$ transformed) after 2 courses of treatment gave the greatest discrimination between patients who were alive or dead at 12 months. No other variables could significantly improve predictive accuracy. Using the classification function derived from this analysis, it was possible to predict correctly 85% of patients who would be dead at 12 months, and 96% of survivors (an overall predictive accuracy of 93%). These results indicate that serum CA125 levels after only 2 courses of treatment can identify good and poor prognostic groups of patients enabling timely and appropriate changes in treatment strategy.

**Chemotherapy type and good performance status improve response in patients with recurrent cervical cancer**

J. Buxton, R. Coleman, K. Towlson, P. Harper,
E. Wiltshaw, J. Dunn, C. Redman & G. Blackledge

*West Midlands CRC Clinical Trials Unit, Queen Elizabeth Hospital, Birmingham; and Department of Medical Oncology, Guy's Hospital, London, UK.*

Response rates in phase II trials of chemotherapy regimens in advanced and recurrent cervical cancer have varied considerably. Despite the development of active regimens the duration of response in the majority of patients is usually short although useful palliation of disease related symptoms may be achieved. In a small but significant group of patients, who achieve a complete response, survival may be improved. Chemotherapy also has potential for up-front use in advanced and bulky early stage disease. High activity chemotherapy is associated with considerable toxicity. A method of identifying patients likely to respond to chemotherapy would be a considerable advance in the treatment of this disease. To determine which if any factors predicted for response and survival an analysis of 171 patients with advanced or recurrent cervical cancer treated in five phase II studies was carried out. The regimens were all based on Ifosfamide (I) ($5\,g\,m^{-2}$ over 24 hours or $7.5\,g\,m^{-2}$ over 5 days) either alone or in combination with bleomycin (30 mg) and/or cisplatin ($50\,mg\,m^{-2}$). Seventy-one patients had single agent I, 49 had B, I and P, 44 had I and P and 7 had B and I. Parameters included in the analysis included initial FIGO stage, histological type and grade, chemotherapy regimen, age, sites of disease, previous irradiation, performance status and time to relapse. Univariate analysis revealed that the likelihood of response to treatment was increased by good performance status at study entry and chemotherapy type (BIP>IP>I>BI) ($P=0.0004$ and $P=0.006$ ($\chi^2$ for linear trend)). Patients who responded survived significantly longer than those with stable or progressive disease. The only other factor associated with survival was performance status on study entry ($P<0.007$). These data indicate that patients with WHO performance status less than two on commencing chemotherapy are significantly more likely to respond to chemotherapy and survive longer than those with a poorer performance status. The data also suggest that, in patients with recurrent disease, combination chemotherapy does not confer a survival benefit when compared to single agent treatment.

**Intensive weekly chemotherapy for good prognosis patients with small cell lung cancer (SCLC)**

D. Miles, P. Harper, H. Earl, R. Souhami, S. Spiro,
R. Rudd, J. Tobias, L. James & K. Ash

*Guy's Hospital, London SE1; University College Hospital, London WC1; The Brompton Hospital, London SW6; and London Chest Hospital, London E2, UK.*

In an attempt to increase the efficacy of chemotherapy in SCLC we have adopted a weekly, intensive, alternating regimen, similar in principle to that used in non-Hodgkin's lymphoma (Klimo & Connors, *Ann. Intern. Med.*, 1985, **102**, 596). Treatment consisted of cisplatin 50 mg $m^{-2}$ i.v. D1 with Etoposide $75\,mg\,m^{-2}$ i.v. D1+2 alternating weekly with Ifosfamide $2\,g\,m^{-2}$ i.v.×1 and Adriamycin $25\,mg\,m^{-2}$ i.v.×1, with six courses of each being given over a total of 12 weeks.

74 patients in the good prognosis category (normal biochemistry, ECOG 0 or 1) have been entered onto the study. 42 patients have completed treatment and are assessable for response and toxicity (31 limited disease, 11 extensive disease, median age 61 years, range 41 to 75). Overall response rate is 38/42 (90%). CR is 23/42 (55%), PR 15/42 (35%). Progression free interval is 46 weeks, median survival has not been reached.

From a total of 467 courses given the number of individual courses complicated by WHO grade 3 or 4 toxicity were; nausea+vomiting, 26 (5.5%), mucositis, 1 (0.2%), leucopenia 55 (12%), thrombocytopenia, 4 (1%), anaemia 16 (3.5%). 70% of patients required red cell transfusion.

Weekly myelosuppressive chemotherapy is possible in SCLC. The overall and complete response rates are high. CI toxicity is manageable and haematological toxicity is predictable. A randomised trial comparing this regimen with a 3 weekly alternating schedule has begun.

**A randomised trial to examine the effect of more extended scheduling of etoposide administration in small cell lung cancer (SCLC)**

P.I. Clark, M.L. Slevin, S.P. Joel, P. Thompson, D. Talbot, C. Price & P.F.M. Wrigley

*Imperial Cancer Research Fund Department of Medical Oncology, St Bartholomew's and Homerton Hospitals, London EC1A 7BE, UK.*

Etoposide is a schedule-dependent drug, as clearly demonstrated by the superiority of 5 consecutive daily infusions over a 24-hour infusion in patients with SCLC (*ASCO* 1986, **5**, 175). A randomised trial has therefore been conducted comparing a more extended 8-day regimen with the 5-day schedule. 77 patients with SCLC (50 ED, 27 LD) were randomised to receive single-agent etoposide $500\,mg\,m^{-2}$, either as 5 daily 2 hour infusions of $100\,mg\,m^{-2}\,day^{-1}$ or as 8 daily 75-minute infusions of $62.5\,mg\,m^{-2}\,day^{-1}$. Both regimens were repeated every 3 weeks for 6 cycles. Single agent carboplatin was given at relapse in both arms of the study. Patients were stratified at randomisation according to their LD/ED status and Karnofsky performance score. No patients have been excluded from analysis. Median follow up time is 24 months. The high single-agent activity of etoposide in untreated patients with SCLC is confirmed. The 5-day and 8-day regimens are equivalent in their activity in SCLC. There was less bone marrow toxicity in the 8-day regimen but more patients with CNS relapse.

| | *5-day* | | *8-day* |
|---|---|---|---|
| *Extensive disease (n)* | 25 | | 25 |
| Response rate (%) | 72 | | 80 |
| Remission duration (months) | 4.7 | | 5.6 |
| Survival duration (months) | 7.0 | | 9.1 |
| Median Karnofsky status (%) | 60 | | 70 |
| *Limited disease (n)* | 14 | | 13 |
| Response rate (%) | 85 | | 93 |
| Remission duration (months) | 5.0 | | 5.8 |
| Survival duration (months) | 12.5 | | 11.5 |
| *All patients* | | | |
| Median day 14 neutrophils, 1st cycle ($10^9\,l^{-1}$) | 0.8 | $P<0.05$ | 1.7 |
| CNS relapse | 5 | $P<0.05$ | 14 |

**Planned versus as required chemotherapy: final report of a randomised trial in small cell lung cancer (SCLC)**

H.M Earl, R.L. Souhami, C.M. Ash, L.E. James, R. Rudd, S.G. Spiro, D.M. Geddes, J.S. Tobias & P.G. Harper

*Dept. of Oncology, Univ. Coll. & Middlesex School of Medicine; Brompton Hospital; London Chest Hospital; and Guy's Hospital London, UK.*

Two different approaches to palliative chemotherapy were compared in a randomised trial in SCLC. All patients received the same chemotherapy (cyclophosphamide 1 g m$^{-2}$ day 1, vincristine 2 mg day 1, etoposide 120 mg m$^{-2}$ i.v. day 1, 100 mg orally b.d. days 2, 3) but were randomised to either planned chemotherapy every 3 weeks, or to as required (PRN) chemotherapy, given only when there was disease progression, or for symptomatic control. Both groups of patients were assessed 3 weekly. A maximum of 8 cycles were given.

300 patients were randomised up to September 1988. In PRN patients the median treatment-free intervals were 42 days and did not decrease in those who continued to receive chemotherapy. PRN patients received approximately half the number of courses of chemotherapy as the planned patients. Overall survival of the PRN patients was not significantly worse than that of the planned patients. Patients with a longer first treatment-free interval survived longer than those with a short interval. Quality of life assessment was made using daily diary cards. In the categories of sickness, appetite, pain, sleep, mood and general 'well-being' the PRN patients consistently scored themselves as having more severe symptoms than the planned patients ($P<0.001$).

The policy of as required chemotherapy is shown to result in less drug treatment for approximately equivalent survival. However, it is not preferred by patients.

**Bone marrow involvement (BMI) by small cell lung cancer (SCLC) using magnetic resonance imaging (MRI)**

D.N. Carney, O. Redmond, P. Harford, J. Stack & J. Ennis

*Departments of Medical Oncology & Radiology, Mater Misericordiae Hospital, Eccles St., Dublin 7, Ireland.*

We have used MR to image the BM of 38 patients with SCLC. Coronal images were obtained of the femoral and pelvic marrow with a 1.5 T Magnetom superconducting magnet system using both T1 weighted (500/17) and T2 weighted (2000/80) spin echo sequences. Discrete foci or patchy areas of altered signal intensity were indicative of marrow abnormality. Results using MR were compared with other staging procedures including CT scan chest and abdomen, radionuclide bone scan, chest X-ray and unilateral BM aspirate and biopsy. BMI was detected using MRI in 19/38 (50%) of patients compared to 2/38 (5%) using standard criteria: in no instance was pathology positive and MRI negative. Among the 38 patients studied, 24 patients had limited stage disease (LD) and 14 patients extensive stage (ED) using standard staging procedures. However, when MRI and BM was included, of the 24 patients with LD, 10 (41%) were now considered to have ED. With combination chemotherapy, resolution of BMI was detectable with MRI. Early CNS relapse was noted in 5/8 patients with LD but who had positive BM MRI. These data suggest: (1) BMI by SCLC can be detected with MRI; (2) the incidence of BMI by SCLC is significantly greater when MRI is used; (3) treatment responses in BM can be monitored using MRI; (4) involvement detected by MRI may predict for early CNS relapse in LD patients.

### ACP – posters

**Treatment of myeloma relapsing after high dose melphalan with continuous infusion vincristine, adriamycin + oral methylprednisolone (VAMP) followed by a second high dose melphalan**

M. Harding, M. Gore, I. Judson, C. Viner, P. Selby, A. Nandi & T. McElwain

*Institute of Cancer Research, Royal Marsden Hospital, Sutton, Surrey, UK.*

High dose melphalan (HDM: 140 mg m$^{-2}$) produces remission in 80% of patients (pts) with previously untreated myeloma. On relapse, pts have received vincristine 0.4 mg + adriamycin 9 mg m$^{-2}$ over 24 hours for 4 days with methylprednisolone 1.5 g orally days 1–5 (VAMP: q 21 days) with or without cyclophosphamide (c: 500 mg weekly) followed by melphalan 180–200 mg m$^{-2}$ with autologous marrow rescue. To date 23 pts have received a second HDM, with an adequate bone marrow harvest (BMH) in 22 pts and ongoing myeloma precluding BMH in 1. Their median age was 45 years (range 26–61). There was 1 fatal septicaemia during 2nd HDM pancytopenia. Fourteen pts responded to VAMP $\pm$C and a further 7 to HDM giving a total response rate of 91%. HDM also converted 4 VAMP $\pm$C partial to compelte remissions. Thus a minimum 48% (11/23) of pts with HDM sensitive myeloma at presentation relapse with HDM sensitive disease.

The median duration of first CR was 15 months (range 4–51) and of PR was 20 months (8–42). Eleven patients have relapsed following 2nd HDM and median duration of second remission is currently 12 months. For patients in whom direct comparison can be made between durations of 1st and 2nd remission, 50% (6/12) had a longer 2nd remission: only 1 of these had VAMP + C resistant disease whereas 5/6 pts with shorter 2nd remissions failed to respond to VAMP $\pm$ C. Thus, although dose escalation of melphalan from 140 to 200 mg m$^{-2}$ is feasible, the relative contributions of a melphalan dose response effect and VAMP $\pm$ C sensitivity to prolongation of 2nd remission remains to be clarified.

**Alfacalcidol as a treatment for low grade non-Hodgkin's lymphoma**

V. Raina, D. Cunningham, N. Gilchrist, R. Cowan & M. Soukop

*Department of Medical Oncology and Department of Biochemistry, Glasgow Royal Infirmary, Glasgow G4 0SF, UK.*

Alfacalcidol has been shown to prolong survival in mouse leukaemia induced by inoculation of leukaemic cells. Malignant and activated T and B lymphocytes have alfacalcidol receptors which are not found on resting lymphocytes. Many malignant cell lines respond to alfacalcidol with inhibition of replication and stimulation of differentiation. Thirty-four patients with progressive low grade (28 follicular small cleaved and 7 small lymphocytic) were treated with 1 $\mu$g of oral alfacalcidol daily. Complete remission was seen in four patients and partial remission in four more, the overall response rate being 23.5%. Disease stabilised in ten patients (29.4%). No response was seen in 16 patients (47%). Three patients who relapsed after discontinuing alfacalcidol responded again after exposure to alfacalcidol. All patients who had initially responded to alfacalcidol but relapsed later, responded to chemotherapy subsequently. Four of the responders to alfacalcidol had previously received chemotherapy. 1,25 $(CH)_2D_3$ receptors were measured from lymph nodes in 11 patients. No correlation was found between the presence of receptors and response to alfacalcidol. Treatment was well tolerated, apart from transient reversible hypercalcaemia in two patients.

Results suggest significant activity of alfacalcidol in a selected group of low grade non-Hodgkin's lymphoma patients with the additional advantage of absence of side effects of chemotherapy. Further research is required to define exactly the mechanism of action and to compare the use of alfacalcidol with other presently available treatment for low grade non-Hodgkin's lymphoma.

**Predicting survival in high and intermediate grade non-Hodgkin's lymphoma (HIG NHL)**

L. Hayward, R. Leonard, R. Prescott, N. Allan, S. Das, A. Davison, H. Lucraft, J. MacGillivray, M. Mackie, A. Parker, S. Proctor, G. Ritchie & T. Sarkar

*Scotland and Newcastle Lymphoma Group (SNLG), Edinburgh EH8 9AG, UK.*

1,130 patients (pts) with working formulation high or intermediate grade NHL, treated with conventional chemotherapy and/or radiotherapy were registered with the SNLG between 1979 and 1987. With median available follow-up of 4.5 years, detailed analysis was performed on all patients <70 years with the aim of identifying poor and good prognostic groups for future therapy studies. The prognostic model was developed using clinical, haematological and pathological data. One test group (202 Edinburgh pts ages <70 years) was excluded to provide a test group for the subsequent model. Using Cox's proportional hazard model best survival was seen in young pts of performance status (PS) 0 with clinical stage I or II disease without liver involvement or weight loss, and with histology other than DUL. Relative risk of death at any time was increased by a factor of 3.0-fold for PS 3–4; 1.9-fold for liver +ve; 1.9-fold for DUL; 1.8-fold for PS 1–2; 1.7-fold for white cell count $<4\times10^9$ $l^{-1}$; 1.4-fold for stage II, III, IV; 1.4-fold for weight loss; 1.22-fold for every decade. Knowing pathology, weight loss and liver status, simple multiples of PS, stage, age and WBC enable the physician to assign a patient to 1 of 3 distinct prognostic groups. The best cohort (25%) have plateau survival of 66% at 5 years in the test group, the worst 25% <5% survival at 5 years (median survival 12 months). Apart from being valuable in future selection of patients for novel therapies these data imply that patient selection may have been very important in determining long term results of previous therapeutic studies in HIG NHL.

**A randomised comparison of low and high dose dexamethasone in combination with haloperidol and lorazepam for control of chemotherapy-induced vomiting**

R. Coleman, Z. Doran, J. Clarke, E. Black, C. Twelves, S. O'Reilly, L. Fleming, M. Richards, R. Rubens & P. Harper

*ICRF Clinical Oncology Unit, Guy's Hospital, London, UK.*

Combination antiemetic regimens have resulted in improvements in chemotherapy-induced nausea and vomiting even though the contributions of individual drugs are often poorly defined. However, most studies focus on cisplatin (DDP) induced vomiting and antiemetic management of non-DDP chemotherapy, particularly in the outpatient setting, has been somewhat neglected.

61 patients received the antiemetic combination haloperidol (S) 3 mg p.o., lorazepam (A) 2 mg p.o. and dexamethasone (D) 5 mg or 20 mg i.v. (double-blind randomisation, parallel design). SAD was given immediately prior to chemotherapy except for DTIC or mustine containing regimens (given >1 hours before) and outpatient treatments (A taken on reaching home). The doses of S and A were reduced in patients >70 years and/or <50 kg, 14 patients received DDP-based regimens and 47 non-DDP based chemotherapy but including highly emetogenic agents. Assessment of efficacy was by a patient-completed diary recording nausea (N), vomiting (V), appetite, drowsiness and acceptability of treatment.

| | | *Severity of nausea* | | | |
|---|---|---|---|---|---|
| | | *None* | *A little* | *A lot* | *Continuous* |
| Non-DDP | $n=47$ (%$n$) | 40 | 30 | 19 | 11 |
| DDP | $n=14$ (%$n$) | 0 | 29 | 14 | 57 |
| D 5 mg | $n=28$ (%$n$) | 29 | 32 | 7 | 32 |
| D 20 mg | $n=33$ (%$n$) | 33 | 27 | 27 | 12 |
| | | *Episodes of vomiting* | | | |
| | | *None* | *1–2* | *2–4* | *>4* |
| Non-DDP | $n=47$ (%$n$) | 49 | 34 | 6 | 11 |
| DDP | $n=14$ (%$n$) | 0 | 43 | 7 | 50 |
| D 5 mg | $n=28$ (%$n$) | 36 | 32 | 4 | 29 |
| D 20 mg | $n=33$ (%$n$) | 39 | 39 | 9 | 12 |

The SAD combination was a safe, practical and effective antiemetic regimen for emetogenic outpatient chemotherapy. It was inadequate for DDP-based chemotherapy. There was no clear difference in efficacy between patients receiving 5 and 20 mg of D although severe nausea and/or vomiting were more common with the lower dose ($P<0.05$). Drowsiness occurred in 92% of patients but only 10% found this unacceptable. Efficacy in non-DDP based chemotherapy was maintained during subsequent courses.

**A single injection of the $5HT_3$ antagonist BRL43694A protects against emesis and would be ideal for outpatient chemotherapy**

D. Cunningham, A. Turner, S. Isles, C. Coulter, H. Thomas, D. Rosin, G. Glazer, R. Witherow & M. Snell

*Ludwig Institute for Cancer Research, St Mary's Hospital Medical School and St Mary's Hospital, London W2, UK.*

This is a report of the use of single injection of BRL43694A at 40 $\mu g\ kg^{-1}$ i.v. over 30 minutes as an anti-emetic in patients naive to chemotherapy. The dose scheduling was selected on the basis of the long plasma half-life (10 hours) and preliminary information showing that the compound may have a clinical effect for up to 24 hours as demonstrated by the flare inhibition model. The patients consisted of 11 males and 6 females, mean age 64 years. Ten were receiving combinations which included cisplatin at a dose of $>50$ mg $m^{-2}$ and the remainder were receiving emetogenic combinations including Doxorubicin and cyclophosphamide. Assessment of emesis control was performed on an inpatient basis for the 24 hours after chemotherapy. During this time, 11 patients experienced no nausea, vomiting or retching and received no additional anti-emetic therapy (7/10 receiving cisplatin regimens and 4/7 receiving non-cisplatin regimens). The remaining 6 patients developed nausea or vomiting between 2.5 and 14 hours after chemotherapy. In no case did the number of episodes of vomiting exceed 4 in the first 24 hours. There were no adverse events reported. In particular, patients did not experience sedation headache or extrapyramidal side-effects. BRL43694A is a well tolerated effective anti-emetic against cytotoxic drug-induced emesis including regimens which contain cisplatin. The very favourable side-effect profile and the 24 hour protection after a single injection indicates it will be of particular value in outpatient chemotherapy.

### Analysis of prognostic factors for response and survival in advanced breast cancer patients receiving first line chemotherapy (CT)

A. Bowman, S.G. Allan, G. White & R.C.F. Leonard

*Imperial Cancer Research Fund Medical Oncology Unit; and Scottish Cancer Trials Office, Western General Hospital, Edinburgh EH4 2XU, UK.*

This paper is an analyis of potential prognostic factors for response and survival in patients (pts) receiving 1st-line CT for relapsed or metastatic breast cancer. 237 pts were studied, median age 50 (26–80), 48% premenopausal, distribution of disease at CT: soft tissue alone 32%, bone $\pm$ soft tissue 14%, visceral $\pm$ any other site 54%. 14 pts remained alive at analysis. Prognostic factors analysed were age, menopausal status, ER status of primary, disease-free interval (DFI), response to prior endocrine therapy, distribution of disease and number of sites involved at CT. Overall response rate to CT was 44%; factors predictive of response were age (45–64 *vs* <45 or >64, $P=0.05$) and use of doxorubicin-containing CT *vs* other CT ($P=0.006$). Median survival from CT was 10.03 months (0.25–104 months). Survival was significantly shorter in pts with visceral disease (lung, liver, marrow, CNS) *vs* non-visceral ($P=0.006$), and in those with multiple *vs* one site of involvement ($P=0.0004$). Within these categories, long DFI (≥20 months) significantly improved survival. No other factors influenced survival from CT. Only patients with 1 site of disease (usually soft tissue) and DFI≥20 months are likely to have relatively long survival from CT (median 22.5 months *vs* 9.4–10.7 months for all other categories).

### A phase I/II evaluation of a novel oral formulation of APD for the treatment of bone metastases

R.E. Coleman, P.J. Woll & R.D. Rubens

*ICRF Clinical Oncology Unit, Guy's Hospital, London, UK.*

The bisphosphonate APD (Pamidronate) is a potent inhibitor of osteolysis. It is the most effective agent for the treatment of hypercalcaemia of malignancy and repeated intravenous administration may promote bone healing and reduce the morbidity of skeletal metastases (Coleman *et al.*, *Br. J. Cancer*, 1988, **58**, 621). In the past the use of oral APD has been limited by upper GI intolerance due to the drug's corrosive properties. We have tested a novel effervescent formulation of oral APD (provided by Ciba-Geigy Pharmaceuticals).

Eleven patients (pts) with bone metastases from breast cancer have been studied. Ten pts had evidence of increased osteolysis (urinary calcium excretion >0.4 mmol mmol creatinine$^{-1}$). The first four pts were treated with 150 mg daily for 4 weeks. Subsequent cohorts of 4 pts received 300 mg and 450 mg daily. Dose escalation within pts was allowed only after completion of four weeks treatment at a constant dose. Pts were assessed weekly for toxicity and biochemical response.

APD was tolerated without toxicity up to a dose of 450 mg a day. In two pts dose escalation to 600 mg a day resulted in unacceptable nausea, vomiting and indigestion. All pts showed a fall in urinary calcium excretion to within the normal range during the first two weeks of treatment indicating adequate absorption of APD and inhibition of bone resorption at all dosages. Serum calcium fell significantly but remained within the normal range.

Effervescent oral APD inhibits bone resorption secondary to breast cancer at doses which are non-toxic. Studies of long-term tolerance of oral APD and its use as an adjunct to sytemic treatment are now indicated.

### The importance of serum aspartate aminotransferase (AST) in breast cancer patients with liver metastases

S.M. O'Reilly, C.J. Twelves, M.A. Richards & R.D. Rubens

*ICRF Clinical Oncology Unit, Guy's Hospital, London SE1 9RT, UK.*

We have studied the significance of AST•measurements in breast cancer patients with liver metastases. The relationship between AST at diagnosis of liver metastases and subsequent survival was investigated retrospectively in 312 patients. Changes in AST occurring during chemotherapy were studied prospectively in 36 patients receiving weekly epirubicin.

263/312 (84%) patients had an elevated AST at diagnosis of liver metastases. There was no difference in survival between patients with normal AST and those whose AST was 1–2 x normal ($P=0.23$). Marked elevation (>2 x normal) of AST was, however, an adverse feature ($P<0.001$) and, within this group, further elevation of AST was strongly associated with a progressively worse prognosis ($P<0.001$ trend test). Multivariate analysis of clinical and biochemical features at diagnosis of liver metastases showed AST to be the most significant variable predicting survival.

Serial measurements of AST from patients receiving weekly epirubicin 25 mg m$^{-2}$ were analysed. All patients had a pretreatment AST >2 x normal. In the 11 patients who had an objective response to treatment, two patterns of change in AST were observed: (a) 7/11 showed steady improvement in AST while (b) 4/11 showed an initial deterioration of >25% in AST during the first month of treatment, with subsequent improvement as chemotherapy continued. In all, 4/20 patients whose AST deteriorated during the first month of treatment subsequently had an objective response.

We have found AST at diagnosis to be the most significant single predictor of survival in patients with breast cancer and liver metastases. An initial deterioration in AST during chemotherapy does not preclude a subsequent response.

### Toxicity of intra-arterial doxorubicin (DOX) in patients with locally advanced breast cancer (LABC)

C.J. Twelves, M. Chaudary, J. Reidy, R.D. Rubens & M.A. Richards

*ICRF Clinical Oncology Unit and Dept of Radiology, Guy's Hospital, London SE1 9RT, UK.*

In patients (pts) with LABC chemotherapy reduces, but does not eliminate, local recurrence. Intra-arterial (i.a.) chemotherapy may increase local drug delivery and enhance cell kill making subsequent local treatment more effective.

We have studied i.a. DOX in pts with inoperable LABC. Under local anaesthetic a catheter was introduced percutaneously via the femoral artery and positioned in the internal mammary artery. The area to be perfused was imaged by a radionucleide perfusion scan. Treatment was with DOX 30 mg m$^{-2}$ by 6 h i.a. infusion on 2 successive days. A second course of i.a. DOX and 2 intravenous (i.v.) courses were planned after which pts were to be graded as operable or inoperable.

Four pts were treated. In each pt the catheter was positioned without complications. Within 48 h of starting i.a. DOX, all 4 developed extensive erythema (WHO grade 1) over the chest wall corresponding to the area shown by the perfusion scan. In 1 pt this progressed to superficial ulceration (grade 3). Erythema resolved in the remaining 3 pts over 1–2 weeks, but 1 pt had a persistent area of increased pigmentation and a marked skin reaction to subsequent radiotherapy. 2 pts developed a raised hemidiaphragm and phrenic nerve paralysis. The incidence of systemic side-effects was similar to that expected after i.v. administration. No pt received more than 1 course of i.a. DOX because of the unacceptable local toxicity. After 3 further courses of i.v. DOX, 2 pts were operable, but 2 were not.

We conclude that although pts with LABC can be treated with i.a. DOX, the toxicity of this schedule is unacceptable. The study closed prematurely, and therefore we cannot assess the activity of DOX given in this way. If this approach to local control is to be studied further in LABC, lower DOX doses of different drugs should be used.

### Mitozantrone (M) and prednimustine (P) in advanced breast cancer (BC)

M.E.R. O'Brien, D.M. Eccles, S.G. Allen, G. Knight, A. Rodger, J.F. Smyth & R.C.F. Leonard

*Imperial Cancer Research Fund Medical Oncology Unit and Radiation Oncology Unit, University Department of Clinical Oncology, Western General Hospital, Edinburgh; Scottish Cancer Trials Office; University Department of Clinical Surgery, Longmore Hospital, Edinburgh, UK.*

Two phase II studies of M+P reported response rates of around 50% in patients with advanced BC. In this trial either 3 or 9 courses of M+P were given to patients with advanced BC (no prior chemotherapy). M was given as $12\,mg\,m^{-2}$ on day 1 with P orally $130\,mg\,m^{-2}$ days 1–5 repeated 4 weekly. Thirty-four patients were treated. The median age was 58 years (range 34–73). Performance status was 0–1 in 26 patients and 2 in 4. Locoregional disease only was present in 13. Nine had lung involvement, 8 liver, 3 bone and 1 stomach. Nine patients had received no prior hormone therapy. Median disease free interval from initial diagnosis was 24 months (range 0–144). 14/23 patients had an oestrogen receptor level of >20 fmol. Two patients are non-evaluable for toxicity or response (disease related deaths). Three others are non-evaluable for response due to progressive disease (PD) after one course. 25/34 patients received >3 courses. Nine patients had grade 2 or more neutropenia with 2 major infections and 1 minor. There was 1 grade 1 thrombocytopenia. Only 6/29 patients had no nausea or vomiting, 14 had grade 1 and 8 patients had grade 2–3. Four patients had grade 2 alopecia. 29 are assessible for response. There was 1 CR and 2 PR both in soft tissue and 1 PR in soft tissue and lung. There were 4 minor responses: one in stomach, 2 PR in soft tissue with SD in liver and lung and 1 SD in soft tissue and an improved bone scan. 14 patients had stable disease after 3 courses, 6 of these lasting >5 months. 11 patients had PD.

The low response rate (14%, 95% confidence intervals 6–35%) is not accounted for by patient selection. One possibility is the relatively low dose – intensity of M compared to the usual dose of 14 mg $m^{-2}$ 3-weekly (i.e. 64% of the usual dose).

### Mitomycin C and vincristine in advanced pre-treated breast cancer

M.J. Millward, A. Hendrick & B.M.J. Cantwell

*University Department of Clinical Oncology, Newcastle General Hospital, Westgate Road, Newcastle-upon-Tyne NE4 6BE, UK.*

We have treated 30 heavily pre-treated patients with advanced breast cancer (median prior chemotherapy regimens 2; median prior hormone therapies 2) with mitomycin C 10 mg total and vincristine 2 mg total every 3 weeks to a maximum of 4 cycles. Objective responses were seen in 4 patients (complete response (CR) 2, partial response (PR) 2), 9 patients had static disease (SD) and 14 progressive disease (PD). Three patients are not evaluable for response. The overall response rate was 15% (95% CI 4–33%). Three responding patients had CR, PR and SD respectively to prior doxorubicin therapy, but none had responded to previous mitoxantrone. Response durations were CR –15 weeks and 16+ weeks, PR –26 weeks and 29 weeks and 4 patients with SD had progression free intervals of >24 weeks. The overall median survival was 35 weeks. Haematological toxicity consisted of 5 patients requiring dose reductions and/or delays because of WHO grade 1 or 2 myelosuppression. One patient was withdrawn because of persisting thrombocytopenia after one cycle of therapy and another patient had persisting thrombocytopenia after 4 cycles of therapy. No grade 3 or 4 haematological toxicity and no renal or hepatic toxicity was seen. Five patients had peripheral and/or autonomic neuropathy requiring cessation of vincristine. Treatment was stopped in one patient because of grade 3 vomiting and in another because of generalised herpes zoster in the absence of myelosuppression.

Low dose, short course mitomycin C and vincristine is a well tolerated regime with moderate activity in this poor prognosis group of patients.

### Phase 1 trial of GR63178A

J. Cassidy, C. Lewis, A. Setanoians, L. Adams, D.J. Kerr, A.H. Calvert, G.W. Brown, A.J. Pateman, E.M. Rankin & S.B. Kaye

*CRC Dept of Medical Oncology, Glasgow University, UK; Royal Marsden Hospital, Sutton, UK; Glaxo Group Research, Greenford, UK.*

GR63178A is a water-soluble analogue of mitoquidone, the first of a new group of pentacyclic pyrroloquinones developed as potential antitumour agents. It has preclinical activity in a range of solid tumours, but not in P388 or L1210 leukaemia. Preclinical toxicology indicated no signs of significant delayed toxicity, and the i.v. $LD_{10}$ in mice was reached at $191\,mg\,kg^{-1}$ ($600\,mg\,m^{-2}$). The starting dose for this phase 1 study was therefore chosen as $70\,mg\,m^{-2}$. The drug is given once weekly for 3 weeks as a 20 minute infusion in 5% dextrose (final concentration not less than 0.5 mg GR63178A per ml). A total of 18 patients (8 male, 10 female) have been entered to date. The mean age is 45.9 years, 5 had colorectal tumours, and 4/18 had received no prior chemotherapy. Five patients received a total of 23 doses at $70\,mg\,m^{-2}$, with minor symptoms of headache, nausea and back pain. Three patients received a total of 13 doses at $90\,mg\,m^{-2}$, and three received a total of 12 doses at $110\,mg\,m^{-2}$. Similar symptoms occurred with some but not all doses at both levels. Seven patients have received a total of 24 doses at $140\,mg\,m^{-2}$. At the highest dose level, potentially dose-limiting side-effects were observed in 2 patients; these comprised severe headache, generalised pain (particularly back pain) and nausea and vomiting.

An HPLC assay has been developed for GR63178A and GR54374 (an active metabolite) with a sensitivity of $10\,ng\,ml^{-1}$ and $2\,ng\,ml^{-1}$ respectively. Preliminary pharmacokinetics suggest that peak plasma levels and AUCs achievable in patients are close to the levels associated with activity in murine tumour models. This study is continuing to define the maximum tolerated dose.

**The stability of the intravenous preparation of etoposide in isotonic fluids**

S.P. Joel, P.I. Clark, M.C. Maclean & M.L. Slevin

*Imperial Cancer Research Fund Department of Medical Oncology, St Bartholomew's and Homerton Hospitals, London EC1A 7BE, UK.*

Etoposide has limited stability in solution but the reported data show a wide variation in results. The stability of etoposide has therefore been re-examined in 2 studies. In the first study solutions of etoposide at a range of concentrations were regularly inspected for precipitation and sampled for HPLC analysis to determine the time at which concentration fell by >10%.

*Study 1*

| *Etoposide ($mg\,ml^{-1}$)* | *Time of precipitate* | *Time of >10% fall in conc.* | *Minimum stability* |
|---|---|---|---|
| 0.25 | >8 days | >8 days | >8 days |
| 0.5 | 30 hours | 30 hours | 24 hours |
| 0.75 | 10 hours | 10 hours | 8 hours |
| 1.0 | 6 hours | 8 hours | 5 hours |

*Study 2*

| *Etoposide ($mg\,ml^{-1}$)* | *Time of precipitate* | *Minimum stability* |
|---|---|---|
| 0.25 | >3 weeks | >3 weeks |
| 0.5 | 11 days | 10 days |
| 1.0 | 24 hours | 18 hours |
| 2.0 | 10 hours | 8 hours |

The stability of etoposide was identical in saline 0.9%/dextrose 5%, and saline 0.15%/dextrose 4% (all in Viaflex infusion bags). No picro etoposide was detected. This study has demonstrated that etoposide stability is concentration-dependent and that the visual detection of precipitate is as sensitive as HPLC in detecting loss of stability. In addition etoposide at a concentration of $0.5\,mg\,m^{-1}$ was stable for 10 days if unsampled, compared with 36 hours if sampled regularly. In a second study the stability of etoposide at 20–23°C in unsampled infusion bags has been determined according to appearance of precipitate. These data show that the stability of etoposide is dependent on concentration but is much greater than previously thought. The appearance of precipitate indicates loss of stability. Considerable saving of etoposide is possible as a whole course of infusions can be prepared at the start of treatment. Previous estimates of etoposide stability have failed to realise the loss of stability that occurs with sampling.

**A pharmacokinetic hypothesis for the clinical efficacy of etoposide in small cell lung cancer**

P.I. Clark, S.P. Joel & M.L. Slevin

*Imperial Cancer Research Fund Department of Medical Oncology, St Bartholomew's and Homerton Hospitals, London EC1A 7BE, UK.*

Etoposide is a phase-specific and schedule-dependent drug. In 2 sequential phase III randomised trials investigating the role of scheduling of etoposide, 3 schedules have been compared. Single-agent etoposide has been given intravenously to patients with untreated extensive small cell lung cancer (SCLC) at the same total dose of 500 mg $m^{-2}$ per course (every 3 weeks) for a maximum of 6 cycles. In the first study a 24-hour infusion was compared with 5 consecutive daily 2-hour infusions. In the second study 5 consecutive daily 2-hour infusions were compared with 8 consecutive daily 75-minute infusions. The pharmacokinetics of etoposide were studied in patients on several occasions. The times over which plasma concentrations of etoposide exceeded 1, 5 and 10 $\mu g\,ml^{-1}$ per course of therapy have been calculated. Median patient and pharmacokinetic data are presented below.

| | *Study 1* | | | *Study 2* | | |
|---|---|---|---|---|---|---|
| | *24-hour* | | *5-day* | *5-day* | | *8-day* |
| No. patients | 20 | | 19 | 25 | | 25 |
| Response rate | 10% | ($P<0.05$) | 90% | 72% | | 80% |
| Remission (months) | – | | 4.5 | 4.7 | | 5.6 |
| Drug AUC ($\mu g\,ml^{-1}\,h^{-1}$) | 483 | | 472 | 512 | | 492 |
| $>10\,\mu g\,ml^{-1}$ (hours) | 24 | ($P<0.05$) | 12 | 12 | ($P<0.05$) | 3 |
| $>5\,\mu g\,ml^{-1}$ (hours) | 32 | | 34 | 33 | ($P<0.05$) | 26 |
| $>1\,\mu g\,ml^{-1}$ (hours) | 49 | ($P<0.05$) | 97 | 101 | | 106 |
| No. etoposide peaks | 1 | | 5 | 5 | | 8 |

The results of study 1 suggested that the superiority of the 5-day schedule was either due to the presence of 5 separate daily exposures to etoposide or to the greater duration of plasma etoposide $>1\ \mu g\ ml^{-1}$. The 8 daily exposures in study 2 failed to increase the duration of remission, but provided a similar duration of plasma etoposide $>1\ \mu g\ ml^{-1}$. This data suggests that the cytotoxicity of etoposide in man, at least in SCLC, is related to maintenance of low plasma concentrations of drug.

**Intraperitoneal cisplatin – a new approach to the adjuvant treatment of gastric cancer**

D. Cunningham, P. Sauven, M. Parker, G. Glazer, D. Coleman, C. Coulter, D. Cunningham, M. Lennox, J. Hermon-Taylor, R.C. Coombes, J.S. Kirkham & R.D. Rosin

*St Mary's Hospital, London; St Georges Hospital, London; QEII Hospital, Welwyn Garden City, UK.*

The five year survival for patients with gastric cancer is 5–10%. Although 30–40% of patients have a potentially 'curative resection' only one-third of these patients remain disease free. Serosal penetration is one of the best predictors of relapse and in Japan has been shown to be associated with malignant cells in peritoneal washings taken at the time of surgery. In view of this we initiated a pilot study to evaluate the feasibility of giving intraperitoneal cisplatin. 14 patients have entered the study. All patients had a curative resection. 12/14 had serosal involvement with tumour. 9/14 had lymph node involvement. Peritoneal lavage with 1 litre of N-saline was performed pre-operatively, immediately postoperatively and before each course of chemotherapy. Cisplatin 60 mg $m^{-2}$ was given intraperitoneally in 2 litres of N-saline via a Tenckhoff catheter (7 patients) or peritoneal catheter (7 patients) every 3 weeks for 4 courses beginning within 6 weeks of surgery. Four of 14 patients had malignant cells in the peritoneal washings taken before chemotherapy. Chemotherapy was generally well tolerated. There were 2 episodes of Tenckhoff catheter sepsis and the catheters were replaced. Technicium was given intraperitoneally before chemotherapy and after 4 courses of chemotherapy in 5 patients and in each case there was good distribution of the fluid in the abdominal cavity. Four of 14 patients experienced nausea and vomiting. There was no renal toxicity. Three of 14 patients have relapsed and 1 has died. One had +ve peritoneal washings. This pilot study demonstrates the safety and feasibility of administering cisplatin 60 mg $m^{-2}$ intraperitoneally following surgery for gastric cancer.

**A phase II study of epirubicin and mitoxantrone in advanced hepatobiliary and pancreatic carcinoma**

C.M. Barton, G.R.P. Blackledge, M. O'Brien & J. Neoptolomos

*West Midlands CRC Clinical Trials Unit, Queen Elizabeth Medical Centre, Birmingham B15 2TH, UK.*

Surgery may cure localised hepatoma and carcinomas of the pancreas and biliary tract but the prognosis of advanced disease is poor even with chemotherapy and radiotherapy. Chemotherapy response rates rarely exceed 20% with responses of short duration. The best results have been reported with doxorubicin or doxorubicin containing chemotherapy. Epirubicin and mitoxantrone have also been shown to be effective and there is *in vitro* evidence of a synergistic effect between doxorubicin and mitoxantrone (Alberts *et al.*, *J. Drug Dev.*, 1988, **1**, 15). A combination of the less cardiotoxic analogue, epirubicin, and mitoxantrone may have the same synergy with fewer side effects. Sixteen patients with locally advanced, recurrent or metastatic hepatoma (8), cholangiocarcinoma (1) and pancreatic carcinoma (7) have been treated with a combination of mitoxantrone, 8–12 mg $m^{-2}$ $day^{-1}$ as a bolus on day 1, and epirubicin 15–25 mg $m^{-2}$ $day^{-1}$ on days 1 to 3, given as a continuous infusion at three weekly intervals, with dose modifications according to the degree of myelosuppression. A total of 52 courses have been administered with 14 patients receiving at least 2 courses and 2 patients completing a maximum of 6 courses. One patient awaits assessment; 9 patients ceased chemotherapy due to disease progression (4 hepatoma, 3 pancreatic carcinoma); 4 had static disease (2 hepatoma, 2 pancreatic carcinoma) and 2 complete responses were seen. Both responders remain in complete remission more than 6 months (pancreas) and 2 years (cholangiocarcinoma) after starting treatment. Myelosuppression, particularly leucopenia, was the most frequent and serious side effect with 25 of the 52 courses complicated by grade 3 or 4 neutropenia, and 17 courses by pyrexia or frank infection. Myelosuppression was more common with doses of epirubicin greater than 20 mg $m^{-2}$ $day^{-1}$. One patient with coincidental septicaemia and steatorrhoea experienced symptomatic hypokalaemia and hypomagnesaemia after her third course of treatment. Alopecia occurred in most patients but other side effects were mild and infrequent and no cardiotoxicity was seen. These data suggest that double intercalating therapy can be administered with myelosuppression being the major toxicity. Responses were seen in tumours generally insensitive to chemotherapy.

**Comparative pharmacokinetics of doxorubicin (DOX) given by bolus injection or 4 day infusion**

C.J. Twelves, N.A. Dobbs, M. Aldhous, S. O'Reilly, M.A. Richards, P.G. Harper & R.D. Rubens

*Clinical Oncology Unit, Guy's Hospital, London SE1 9RT, UK.*

Although there has been much interest in dose intensity, drug scheduling has been studied less widely. We have compared the pharmacokinetics of DOX given by i.v. bolus or 4-day infusion in 11 patients (pts) with advanced breast cancer, normal liver biochemistry and no evidence of liver metastases. Six pts were treated with DOX 70–75 mg $m^{-2}$ i.v. bolus on day 1 and blood samples collected at timed intervals for 72 hours after injection. Five pts received DOX 60–75 mg $m^{-2}$ by continuous i.v. infusion days 1–4 via a Hickman indwelling catheter using a Travenol infusor. Blood samples were collected during the infusion and for 48 hours after its completion.

Plasma levels of DOX and its metabolite doxorubicinol (DOXol) were assayed by high performance liquid chromatography (HPLC) with fluorimetric detection. Peak plasma concentrations (PPC) of DOX and DOXol were measured, areas under the concentration-time curves (AUC) derived by the trapezoidal method and clearance (Cl) calculated; the white blood count (WBC) was checked on day 10–12. Results are shown below as mean values and the groups compared using Wilcoxon's rank sum test.

| | *PPC* ($ng\,ml^{-1}$) | | *AUC* ($ng\,ml^{-1}\,h^{-1}$) | | *Cl* ($m^{-2}\,h^{-1}$) | | *WBC* ($\times 10^9\,l^{-1}$) |
|---|---|---|---|---|---|---|---|
| | *DOX* | *DOXol* | *DOX* | *DOXol* | *DOX* | *DOXol* | *d10–12* |
| Bolus | 4,278 | 39.1 | 2,787 | 1,006 | 28.1 | 79.1 | 2.1 |
| Infusion | 107 | 28.3 | 5,617 | 2,260 | 12.5 | 38.3 | 3.7 |
| *P* value | <0.01 | NS | <0.01 | NS | <0.01 | <0.05 | NS |

Despite a similar dose intensity, 4-day infusion significantly reduced peak DOX levels, but increased total drug exposure. Drug scheduling may have an important effect on the efficacy and toxicity of doxorubicin.

**Quantitative distribution of $^{131}I$ labelled monoclonal antibodies administered by the intra-ventricular route**

D.B. Smith, R.H.J. Begent, M. Glaser, S. Dewhurst & A. Kelly

*Cancer Research Campaign Laboratories and Dept of Radiotherapy, Charing Cross Hospital, London, UK.*

Tumours of the CNS often remain confined to the neuraxis throughout their natural history and thus intra-cavity radiolabelled monoclonal antibody treatment represents an attractive therapeutic option. In order for such therapy to be effective there must be distribution of the antibody complex throughout the CSF and in this study we make a quantitative assessment of distribution in 3 patients following administration via the intra-ventricular route. The patients were (1) a 34-year-old man with CNS relapse from a teratoma (SB10 × W14 monoclonals raised against HCG were used), (2) a six-year-old with a relapsed primitive neuro-ectodermal tumour (antibody used was UJ13A), (3) a 22-year-old with a recurrent medulloblastoma (antibody used MEL14). The antibodies were labelled with 20–50 mCi $^{131}I$ and were administered in each case via an Ommaya reservoir. Patients were scanned using an IGE Gemini 700 gamma camera at intervals up to 10 days post injection. Counts were assessed over the head, upper half spine and lower half spine. In all 3 patients there was distribution of radiolabel throughout the neuraxis reaching the three study areas within 24 hours. In the first 2 patients 41–56% of the counts at each time point remained in the head with 18–39% in each of the upper and lower spine areas. Similar results were achieved in the third patient despite a partial block to the flow of CSF although distribution took 12–24 hours longer. The $T_{1/2}$ for total neuraxis counts was 31.5 h in patient 1, 19.8 h in patient 2 and 15.5 h in patient 3. There was no evidence of antibody binding to known sites of tumour.

The data indicate that adequate distribution of radiolabelled monoclonal antibodies can be achieved using the intra-ventricular route even in the presence of partial CSF block.

**Peritoneal lavage fluid (PLF) CA125: a tumour marker and independent prognostic factor in epithelial ovarian cancer (EOC)**

C.W.E. Redman, D.M. Luesley, G. Blackledge, E.J. Buxton & K.K. Chan

*West Midlands CRC Clinical Trials Unit, Queen Elizabeth Medical Centre, Birmingham B15 2TH, UK.*

The monitoring and assessment of response in EOC can be difficult, especially when the disease burden is small. Serum CA125 is more sensitive than clinical and radiological modes of assessment and its levels have an excellent correlation with disease burden. Unfortunately there is a significant false negative rate when the amount of residual disease is small. As EOC is largely an intraperitoneal disease, theoretically intraperitoneal CA125 levels may be a more sensitive index of disease status. Following peritoneal lavage with 1 l of 0.9% saline, CA125 levels in peritoneal lavage fluid were measured 40 healthy controls and 67 EOC patients with residual disease prior to treatment. There was a highly significant difference in both serum ($t_{105}=8.0$; $P<0.001$) and PLF CA125 ($t_{105}=5.6$; $P<0.001$) levels between the two groups of patients. The presence of ascites and amount of residual disease had a significant association with serum and PLF CA125 levels but there was no significant association with histological type and grade of differentiation. Cut-off point for serum and PLF CA125, that achieved the maximum discrimination between cancer and control patients were 30 and 60 U ml$^{-1}$ respectively. On the basis of these values the sensitivity for serum and PLF CA125 were 89.5 and 64.2%, and overall predictive value 91.5 and 77.6% (Mcnemar $\chi_1^2=9.0$; $P=0.003$). Although not as sensitive as its serum counterpart, PLF CA125 was better than clinical findings, ultrasound, CT scan and peritoneal cytology in detecting residual disease. In 59 evaluable EOC patients changes in serial PLF CA125 levels corresponded with observed response in 71% of cases compared to 84% for serum CA125. PLF CA125 was a poor marker of response when initial levels were negative. To assess the prognostic significance of PLF CA125 levels at the start of treatment in conjunction with other prognostic parameters, including serum CA125, an initial univariate and subsequent multivariate analysis was performed. Stage, size of residual disease, positive peritoneal cytology, the presence of ascites, serum and PLF CA125 levels were identified as important prognostic variables on the basis of univariate log-rank analysis. Because of the interaction between these factors, and as neither serum nor PLF CA125 conformed to the proportional hazards model, a multivariate stepwise discriminant analysis was performed, using survival at 12 months after primary surgery as the end point. The presence of ascites and PLF CA125 levels prior to treatment achieved the greatest discrimination correctly predicting the outcome at 12 months in 81% of patients. These findings indicate that whilst PLF CA125 is not a more sensitive tumour marker than its serum counterpart its pre-treatment levels have an independent prognostic value.

**Analysis of survival in epithelial ovarian carcinoma**

D.M. Eccles, S.G. Allan & R.C.F. Leonard

*Department of Clinical Oncology, Western General Hospital, Edinburgh EH4 2XU, UK.*

Survival from epithelial ovarian carcinoma has not improved significantly over the past 5–10 years (yr) and it is now the leading cause of death from gynaecological cancer. Several factors have been identified as having some prognostic significance including FIGO stage, age, performance status, ascites adhesions, extent of debulking surgery and histological type and grade. In the present series of 320 patients presenting to the Department within the years 1975–88, 109 had FIGO stage I and II (limited stage) disease and 205 had stage III and IV (advanced stage) disease. The mean age of all patients was 57.8 yr (range 28–82). Stage IV mean age 64 yr, stage III mean age 58.6 yr (23–82), stage II mean age 60.9 yr (45–78), stage I mean age 53.6 yr (31–79). In advanced stage disease 30% had good debulking surgery (maximum dimension of residual tumour <2 cm), mean survival (MS) was 18.8 months (m) (median 16 m); 30% of advanced stage disease was partially debulked (residual tumour <5 cm maximum dimension), MS was 16.2 m (median 12 m); 34% had no debulking of advanced disease and MS was 12.2 m (median 8 m). In 6% surgical debulking was not assessable. Only 4 patients with advanced disease were still alive at 5 yr. Chemotherapy used in advanced disease was a single alkylating agent regimen, 54 patients, MS 15.4 m (median 9 m); single agent cisplatinum, 22 patients, MS 16 m (median 12 m); or a cisplatinum combination regimen, 66 patients, MS 16.5 m (median 15 m). Data on haematological and biochemical parameters recorded within 8 weeks of operation will be included in the multivariate analysis of prognostic factors.

**Phase I and pharmacokinetic study of thiotepa (T) administered intraperitoneally (i.p.) to patients with ovarian cancer**

C.R. Lewis, N. Lawson, E.M. Rankin, G. Morrison, J. Cassidy, D. Kerr, A. MacLean, J. Cordiner & S.B. Kaye

*Dept of Medical Oncology & Dept of Gynaecology, University of Glasgow, UK.*

Fifteen patients with ovarian cancer, all of whom had previously received first line systemic chemotherapy with platinum analogues or cisplatin, were given T via a Tenckhoff peritoneal dialysis catheter in 2 l of normal saline with a 4 h dwell time. Doses ranged from 30 mg m$^{-2}$ to 80 mg m$^{-2}$. Dose limiting toxicity was myelosuppression, which occurred at 80 mg m$^{-2}$ and was frequently prolonged (white cell count WHO grade 3, 5pt; grade 4, 1pt; platelets WHO grade 3, 3pt; grade 4, 1pt). Short lived nausea and vomiting (WHO grade 2 or less) was easily controlled, and there was no local toxicity. Five patients remain free from disease progression 2 to 7 months after commencing intraperitoneal T, and 3 patients had temporary reduction in ascites formation.

Thiotepa concentrations were measured by gas chromatography. Peritoneal fluid concentrations declined in a first order fashion with a half-life of 1.1 h. A mean of 90% of the drug was absorbed during the 4 h dwell time. Plasma levels peaked 1–1½ h after drug instillation and were substantially lower than corresponding peritoneal levels. Maximum peak level peritoneal/plasma pharmacological advantage was 30 (mean 25), and mean AUC advantage was 9.

This study demonstrates a selective pharmacological advantage for the i.p. delivery of T, and the treatment is well tolerated. The recommended dose for i.p. T is 60 mg m$^{-2}$ every 3–4 weeks.

**High dose intensity combination chemotherapy with cisplatin and cyclophosphamide for advanced epithelial ovarian carcinoma – results of a pilot study**

J.W. Sweetenham & C.J. Williams

*CRC Wessex Medical Oncology Unit, Southampton General Hospital, Southampton SO9 4XY, UK.*

The influence of dose intensity on outcome for patients (pts) with advanced ovarian carcinoma treated with chemotherapy has been the subject of a large retrospective study by Levin and Hrynuik (*J. Clin. Oncol.*, 1987, **5**, 756). A significant correlation was demonstrated between average relative dose intensity (ARDI), response rates and survival. As part of a proposed randomised trial to assess the effect of dose intensity on outcome in this disease, we undertook a pilot study to determine the toxicity and tolerability of the high intensity therapy. Eligibility: histologically confirmed epithelial ovarian cancer, age 16–75, KPS > 50%, FIGO stage II with > 2 cm res. disease, FIGO stage III or IV, normal FBC, creatinine clearance > 70 l/24 h. Chemotherapy: cisplatin 120 mg m$^{-2}$ i.v. d1., cyclophosphamide 1000 mg m$^{-2}$ i.v. d1. q = 21 days. No. of cycles = 6. ARDI = 1.14 (using CHAP regimen (Greco *et al.*, *Obstet. Gynecol.*, 1981, **58**, 200) as standard ARDI of 1). Patient characteristics: 16 entered, median age = 62 years (34–72). FIGO stage II, 3; III, < 11; IV, < 2; > 2 res. dis., < 11; < 2 cm res. dis., < 5. 87 courses of chemo have been given. 42 courses have been delayed or given at reduced dose because of leucopenia (14) or renal impairment (28). Only 1 pt has received the total planned dosage of chemotherapy at an ARDI of 1.14. Median received dose intensity = 0.93 (0.61–1.14). Toxicity: all pts had WHO grade 2 or 3 nausea and vomiting and alopecia. Treatment was discontinued in 2 pts because of severe ototoxicity. Nine pts had peripheral neuropathy which was disabling in 4. Clinical response: CR, < 9; PR, < 4; PD, < 2; inev., < 1. Conclusion: Prospective randomised trials of dose intensity are essential. An initial step is to test whether the projected high intensity arm can be delivered. In this study we were only able to administer chemotherapy of an ARDI of 0.93. Although this is considerably less than the projected dose, it still approaches that of the standard regimen (CHAP). A regimen of such a dose intensity may be suitable for a randomised trial.

### Cerebral metastases in small cell lung cancer (SCLC) and non-small cell lung cancer (NSCLC) treated by chemotherapy

C. Twelves, R. Souhami, P. Harper, J. Tobias, S. Spiro, D. Geddes, C. Ash & J. Drake

*Clinical Oncology Unit Guy's, University College & Middlesex Medical Schools, Brompton & London Chest Hospitals, London, UK.*

Cerebral metastases are conventionally treated by cranial irradiation even when the primary tumour may be chemosensitive because it is assumed they are protected by the blood brain barrier (BBB). We have assessed response in SCLC and NSCLC patients with symptomatic, CT proven cerebral metastases at presentation treated by chemotherapy alone. Patients did not receive cranial irradiation; steroids were given where clinically indicated but were reduced or withdrawn during chemotherapy.

In 25 SCLC patients treatment was with cyclophosphamide 1 g m$^{-2}$ i.v. day 1, vincristine 2 mg i.v. day 1 and etoposide either 100 mg p.o. tds days 1–3 or 120 mg i.v. day 1 and 100 mg p.o. bd days 2 and 3 on a 21-day cycle. On repeat brain scan 10 of 17 patients responded. In responders who were scanned after only 1 course of chemotherapy, response was detectable at this early stage. Eight patients were assessed only clinically and 3 responded. All cranial responders improved on chest X-ray. Overall NSCLC cranial response rate was 2/6 (33%).

We conclude that symptomatic SCLC and NSCLC cerebral metastases are not protected from chemotherapy by the BBB. Indeed, the 'blood–tumour barrier' may be the same in these metastases as other sites of metastatic disease. Chemotherapy has the advantage of treating both cerebral metastases and extra-cranial tumour.

### Educating the public about testicular cancer – experience with university students

S.M. Crawford, G.B. Littlejohn & P.G.O. Kamill

*Clinical Oncology Unit, Dept of Social Analysis, Student Health Service, Bradford University, Bradford BD7 1DP, UK.*

The treatment of testicular cancer carries a higher morbidity, and its success may be compromised, when patients present with very advanced disease. Such presentation is usually associated with delay on the part of the patient or his doctors. The delay might be reduced by educating patients about the significance of gonadal abnormalities.

Male students at this University were offered a leaflet at the time they collected their grants in April and October 1988, which described testicular self examination (TSE). Consultations for scrotal lesions at the Health Centre were monitored and in January 1989 students were interviewed about the leaflet.

Nine consultations occurred, none with findings indicative of tumours. Further consultations took place during the period of the survey. Preliminary analysis of the survey based on 171 interviews shows that only 21.6% male students recalled seeing the leaflet. Of these 70.2% knew that TSE was to detect cancer compared with 27.6% of those did not remember the leaflet ($P < 0.001$). Half of those who had seen the leaflet had performed TSE compared with 3% of those who had not seen it.

The TSE leaflet is a useful method of educating the public but a more positive method of drawing it to people's attention is needed.

### BACR – oral presentations

### Investigations of the mechanisms of epithelial carcinogenesis using XB2 keratinocytes

S.D. Hewitt, P.A. Duffy, R.C. Garner & T.C. Orton

*Cancer Research Unit, University of York, Heslington, York YO1 5DD; ICI Pharmaceuticals, Mereside, Alderley Park, Macclesfield, Cheshire SK10 4TG, UK.*

XB2 keratinocytes, derived from Rheinwald and Green's teratoma line are routinely cultured in our laboratory in a low calcium medium (0.03 mM) and used in the study of mechanisms of epithelial carcinogenesis. When cultured at low density (50 cm$^{-2}$), cells begin to differentiate and this process can be affected by certain carcinogenic agents. At high density (5000 cm$^{-2}$), there is less tendency to differentiate and the process will be perturbed by only the strongest carcinogens, e.g. DMBA. The degree of differentiation can be assessed by measurement of keratin production and stratification; cell numbers are used as an index of proliferation.

Using this system, we have found that the strong stage I and II promoters TPA, teleocidin and okadaic acid all greatly increase parameters of proliferation and differentiation but, whereas okadaic acid achieves this at low density only, the protein kinase C-mediated promoters also exert an effect at high density. The stage II promoter mezerein affected stratification only. BHA, the non-mutagenic carcinogen of rodent forestomach, had opposite effects to its non-carcinogenic partner BHT. BHT increased proliferation only and BHA increased only the parameters of differentiation.

These data suggest that the mechanism of carcinogenesis by BHA does not involve protein kinase C but is possibly similar to the mechanism of action of mezerein. This system of low/high density culture also seems to provide a method whereby non-mutagenic carcinogens may be recognised as a group distinct from promoters.

**Amplification of specific DNA sequences in C127 mouse cells transformed by bovine papillomavirus type 4**

K.T. Smith & M.S. Campo

*The Beatson Institute for Cancer Research, Garscube Estate, Bearsden, Glasgow G61 1BD, UK.*

The mode of malignant transformation of cells by BPV-4 is the result of a 'hit and run' mechanism: viral DNA is always found in oesophageal papillomas whereas the viral genome is rarely detected in carcinomas arising from those papillomas. Furthermore, BPV-4 DNA can transform C127 mouse fibroblasts *in vitro* but again the viral genome is often absent from the transformed cells, indicating that the viral DNA is not involved in the maintenance of the transformed state. As an initial attempt to characterise the event(s) initiated by the virus, leading to the stably transformed phenotype, we have used high molecular weight DNA from the BPV-4-transformed cells to re-transfect C127 cells. This resulted in morphological transformation of the C127 cells in the absence of any BPV-4 DNA, thus the virus has activated a dominant oncogene. Southern blot analysis of DNA from these secondary transformants demonstrated that there was amplification of a specific DNA sequence, previously isolated from a BPV-4-transformed cell line where it was linked to a rearranged viral genome. Amplification of this, or a related sequence, was also observed in a chemically induced mouse skin carcinoma. Therefore, we have identified an oncogene that is activated by BPV-4 resulting in malignant transformation of cells. We are currently investigating the mechanism of this activation.

This research was supported by the Cancer Research Campaign.

**What is the role of c-*erb*B-2 protein in Paget's disease of the nipple?**

D. M. Barnes, G.A. Lammie, R.R. Millis & W.J. Gullick

*ICRF Clinical Oncology Units, Guy's Hospital, London SE1 9RT, and Hammersmith Hospital, London W12 0HS, UK.*

Amplification of the c-*erb*B-2 oncogene in mammary carcinoma has been proposed as an indicator of poor prognosis. Antibodies are now available and comparative studies have found a close relationship between positive immunohistochemical staining of sections of human breast tumours and amplification of the gene. Retrospective studies on archival material from patients with extended follow up are therefore possible. We have carried out two such studies using antibody 21N (Gullick *et al.*, *Int. J. Cancer*, 1987, **46**, 246) with a peroxidase conjugated avidin-biotin technique. We have examined the presence of c-*erb*B-2 protein in 195 infiltrating carcinomas and in tissue from 45 patients with Paget's disease of the nipple. When the staining patterns of the 195 patients, who had a 10-year follow-up, were evaluated, strongly positive membrane staining was found in 9% of the tumours. An association between positive staining and histological grade III tumours ($P=0.04$) was the only significant finding. Although log rank analysis did reveal a tendency for patients with unstained tumours to have a slightly better prognosis, this trend disappeared in a multivariate Cox regression analysis. When we examined the tissue from the 45 patients with Paget's disease of the nipple we found 90% positivity, both in the Paget cells and in the underlying carcinoma. In 35% of the cases the underlying carcinoma was purely *in situ* and in 65% infiltrating tumour was also present. In the majority of cases the malignant cells in both components were large and pleomorphic and the *in situ* ductal carcinoma was of the comedo type. It is suggested therefore, that c-*erb*B-2 amplification is related to this architectural and cytological pattern of ductal carcinoma and may play a role in the manner of local spread of tumour cells seen in Paget's disease of the nipple.

**Biochemical characterisation and localisation of normal *bcr* gene products**

S. Dhut, T.S. Ganesan, T. Chaplin & B.D. Young

*ICRF, Department of Medical Oncology, St Bartholomew's Hospital, West Smithfield, London EC1A 7BE, UK.*

A monoclonal antibody (7C6) derived against a synthetic *bcr* peptide was used to study normal *bcr* gene products. In $^{35}$S-methionine labelled KG1 cells, a *bcr* phosphoprotein of 130 kDa was demonstrated in addition to the previously identified *bcr* phosphoproteins of 160 kDa. Sequential immunoprecipitation demonstrated both p130 and p160 had determinants from two separate regions of the putative *bcr* translated sequence. The rate of synthesis of normal *bcr* products as estimated by metabolic labelling was comparable with that of p210 *bcr-abl* in BV173 and K562 cells. In K562 cells, the *in vivo* phosphorylation of p160 exceeded that of p130 and both normal products were unaffected by the increased phosphorylation of p210 *bcr-abl*. The half-life of both normal *bcr* products was estimated to be more than 48 hours by pulse-chase experiments. Immunofluorescence analysis by conventional and confocal microscopy established that both normal *bcr* products were located predominantly in the cytoplasm. This was confirmed by subcellular fractionation of $^{35}$S-methionine labelled cells and immunoprecipitation with 7C6 antibody. In conclusion, there are two normal *bcr* gene products, p160 and p130, both phosphoproteins located in the cytosol, and the level of expression of p210 *bcr-abl* and the normal *bcr* products are similar in cell lines K562 and BV173.

**Association of an abnormal c-*raf*-1 gene, a radioresistant phenotype and aberrant topoisomerase II activity in a cancer-prone family**

J.M. Cunningham, K. Pirollo, E. Chang & G.E. Francis

*Molecular Cell Pathology Unit and Department of Haematology, The Royal Free Hospital, London NW3; and Dept of Pathology, Uniformed Services University of the Health Sciences, Bethesda MD 20814 USA.*

Susceptibility to mutation at many sites in the genome is consistent with the behaviour of malfunctioning DNA topoisomerases *in vitro*. Studies of the Li-Fraumeni cancer family syndrome show an association between susceptibility to a wide variety of neoplasms, inheritance (in non-cancerous cells) of the c-*raf*-1 oncogene and a radioresistant phenotype. This association, and the fact that topoisomerases are known to be activated *in vitro* by serine/threonine kinases similar to *raf*, prompted investigation of DNA topoisomerase activity in non-cancerous fibroblasts from a cancer-prone family. Since radioresistance is transferred to HIH-3T3 cells when the family's aberrant c-*raf*-1 gene is transfected, we also examined transformants containing this and other oncogenes (c-*myc* and EJ-*ras*). The latter were examined because *myc* is amplified 3–8-fold in lines derived from this family, but does not appear to convey the radioresistant phenotype and because radioresistance can also be conveyed by several other oncogenes including *ras*. All three family members cell lines and three transfected mouse lines containing either the abnormal c-*raf*-1 or *ras* oncogenes or v-*raf* with *myc*, showed a similar significant perturbation of a spermidine and ATP dependent DNA catenation activity typical of DNA topoisomerase II. Relaxation of DNA supercoiling (DNA topoisomerase I activity and other DNA nicking enzymes) was not abnormal. Since topoisomerases are error prone, particularly when their activity is disturbed, these findings may be relevant to the mechanisms of oncogenesis and related radioresistance in this family.

### A new *in vitro* colony assay for multipotential progenitors in human bone marrow peripheral blood

I.B. Pragnell, M. Freshney, A. Sproul, D. Kerr, D.G. Wright, N. Lucie, N. Wilkie & G. Konwalinka

*Beatson Institute, Glasgow; MRC Radiobiology, Didcot; Western Infirmary, Glasgow; University of Innsbruck, Austria.*

The physiology of human hematopoietic stem cells can be studied in normal and diseased states by clonal *in vitro* cultures in which the primitive progenitor cells proliferate and differentiate into different lineages to form mixed colonies. For many applications it is essential that such assays detect a high proportion of primitive progenitor cells. We describe an *in vitro* assay for human pluripotent progenitor cells which detects a considerable proportion of the primitive progenitor compartment. Mononuclear cells from bone marrow were plated at low cell concentrations in semisolid agar cultures with recombinant human granulocyte-macrophage colony-stimulating factor. These culture conditions support the formation of macroscopic multilineage colonies at an average incidence of $137 \pm 85$ (mean $\pm$ s.d.) per $10^5$ mononuclear bone marrow cells. The colony forming cells, (CFR-A, colony forming unit, type A) detected in normal bone marrow give rise to colonies displaying a marked heterogeneity, a variable cycling status and a high replating efficiency. These observations are consistent with the CFU-A being primitive progenitor (stem) cells. Colonies with similar properties could also be detected upon culture of mononuclear cells from peripheral blood at a 10–20-fold lower incidence. The demonstration of fluctuatary levels of CFU-A in peripheral blood after cytotoxic drug treatment suggests that the assay could be exploited for clinical use. The application of this technique to the analysis of neoplastic haemopoietic cells will be discussed. The studies are supported by grants from the Cancer Research Campaign.

### Onco-suppressor action of the normal human H-*ras*1 gene

D.A. Spandidos & N.M. Wilkie

*The Beatson Institute for Cancer Research, Garscube Estate, Bearsden, Glasgow G61 1BD, UK; and Biological Research Center, National Hellenic Research Foundation, Athens, Greece.*

The transformed phenotype of rat 208F cells transfected with the T24 H-*ras*1 oncogene is suppressed by simultaneous or subsequent transfection with the normal H-*ras*1 gene. The suppressed cells express both the normal and mutant forms of *ras* p21 but the normal form predominates. Rare transformed cells obtained after simultaneous transfection express mainly the T24 p21. Some suppressed cells induce tumours in nude mice after a long lag period and these tumour cell lines have much reduced expression of normal p21. The normal H-*ras*1 gene also suppresses the transformed phenotype induced by mutant N-*ras*, albeit less effectively. The tumorigenicity of the EJ bladder carcinoma cell line, which contains only the T24 mutant allele of H-*ras*1, is also suppressed following transfection with the normal H-*ras*1 gene. The results suggest that transforming alleles of *ras* genes do not behave in a truly dominant manner and that expression of the normal allele at elevated levels can lead to suppression of the transformed and tumorigenic phenotypes.

### The effect of H-*ras* and c-*myc* oncogene transfection on response of epithelial cells to growth factors and cytotoxic drugs

D.J. Kerr, J. Plumb, M. Khan, R.I. Freshney & D.A. Spandidos

*CRC Dept Medical Oncology & Beatson Institute for Cancer Research, Glasgow, UK.*

Mink lung epithelial cells were transfected with c-*myc* and activated H-*ras* genes. The transfected sublines formed colonies in soft agar and were tumorigenic when injected subcutaneously into athymic nude mice. DNA synthesis was measured in each of the cell lines by $^3$H-thymidine incorporation and in the parent line there was dose related stimulation of DNA synthesis by epidermal growth factor [EGF: 6 fold stimulation at 1 ng ml$^{-1}$) and inhibition by transforming growth factor-$\beta$ (TGF-$\beta$: $ID_{50} = 310$ pg ml$^{-1}$). The c-*myc* transfected line had a reduced inhibitory response to TGF-$\beta$ ($ID_{50} = 3{,}880$ pg ml$^{-1}$) and an exaggerated stimulatory response to EGF (8.5-fold increase at 1 ng ml$^{-1}$) whereas the activated H-*ras*1 transfected line did not respond to TGF-$\beta$ and EGF. Cellular sensitivity to cytotoxic drugs was assessed using a colorometric assay based on the ability of living cells to reduce a tetrazolium dye. The activated H-*ras*1 transfected line was significantly more resistant to doxorubicin ($ID_{50}$, 2.4nM) than the parent mink lung epithlial cell line ($ID_{50}$, 1.4nM). Incubation of the different cell lines with TGF-$\beta$ and EGF for 24 hours before and during drug exposure did not alter their sensitivity to doxorubicin. It would appear that oncogene transfection can alter the sensitivity of mink lung epithelial cells to both exogenous growth factors and cytotoxic drugs.

### Carboplatin dose determination using a simple formula: single agent, combination and high-dose studies

D.R. Newell, L.A. Gumbrell, F.E. Boxall, R.A. Eeles, A. Horwich & A.H. Calvert

*Institute of Cancer Research and Royal Marsden Hospital, Sutton, Surrey, UK.*

Carboplatin is an active platinum complex with reduced non-haematological toxicities. However, the full therapeutic potential of the drug may not have been realised because of inadequate dosing. Carboplatin is cleared primarily by urinary excretion (about 70% dose in patients with normal renal function), so that patients with impaired or higher than average renal function may experience over or under dosing, respectively. We have developed (Newell *et al.*, *Br. J. Cancer*, 1987, **56**, 233) a carboplatin dosage formula which compensates for variations in glomerular filtration rate (GFR) and gives doses which result in far more consistent drug exposure than doses calculated on the basis of surface area, i.e. dose (mg) = target AUC $\times$ (GFR + 25). In the equation AUC is the required area under the 0–24 h free carboplatin plasma concentration *vs* time curve and GFR is the absolute value (ml min$^{-1}$) as measured by $^{51}$CrEDTA, *not* creatinine, clearance. In previously treated patients given single agent carboplatin, an AUC of 4–6 mg ml$^{-1}$ was well tolerated (platelet count at week 3, 36–83% pretreatment) while in previously untreated patients an AUC of 6–8 mg ml$^{-1}$ min$^{-1}$ was associated with manageable thrombocytopenia (3 week count 36–73% pretreatment). In combination studies with other myelosuppressive agents the target AUC should be reduced and with carboplatin, etoposide ($120\,mg\,m^{-2} \times 3$) and bleomycin (30 mg weekly), for the treatment of testicular teratoma, the formula has been used with a target AUC of $4.5\,mg\,ml^{-1}\,min^{-1}$. In a crossover study in 4 patients (8 courses of carboplatin, 16 courses of etoposide) there was no interaction between carboplatin and etoposide pharmacokinetics and the schedule was well tolerated. The formula accurately predicted the carboplatin dose required to achieve the desired AUC (observed AUC $4.8 \pm 0.6\,mg\,ml^{-1}\,min^{-1}$). Retrospective analysis of pharmacokinetics following high-dose carboplatin ($800–1600\,mg\,m^{-2}$) also indicated that the formula is predictive (AUC range $10–28\,mg\,ml^{-1}\,min^{-1}$, observed/predicted ratio $1.01 \pm 0.15$). To simplify future studies, the relationship between AUC and the 24 h total plasma platinum level was investigated. After 58 courses (AUC range $0.1–28\,mg\,ml^{-1}\,min^{-1}$) there was a highly significant correlation ($r = 0.96$, $P < 0.00001$) and hence the AUC achieved can be determined by a single plasma platinum determination. Thus the dose formula provides a simple and consistent method of determining carboplatin doses for single agent, combination and high dose studies. Its application should allow the full therapeutic potential of carboplatin to be determined and exploited.

### Preclinical and clinical pharmacokinetic studies with 1-(4-carboxyphenyl)-3, 3-dimethyl triazene (CB10-277)

B.J. Foster, D.R. Newell, J. Carmichael, A.L. Harris, L.A. Gumbrell, D.E.V. Wilman & A.H. Calvert

*Inst. Cancer Res., Sutton, Surrey; Newcastle General Hospital, Newcastle-upon-Tyne, UK.*

CB10-277 is an analogue of dacarbazine (DTIC) which is being evaluated in early clinical trials. DTIC and CB10-277 require metabolic activation (*N*-demethylation) to a monomethyl (MM) metabolite and in preclinical experimental systems possess equivalent antitumour efficacy. As part of the preclinical and phase I evaluation of CB10-277 the pharmacokinetics in patients and mice have been compared. In mice treated with an i.v. maximum tolerated dose (MTD) of $750\,mg\,m^{-2}$, CB10-277 was cleared monophasically from plasma ($25\,ml\,min^{-1}\,m^{-2}$), $t_{\frac{1}{2}} = 32$ min. One major and two minor metabolites were found in mouse plasma using HPLC analysis. The major metabolite was sensitive to hydrolysis by glucuronidase yielding CB10-277. One of the minor metabolites was identified by HPLC as the MM derivative of CB10-277 with levels up to 50 $\mu$M at the MTD. Urinary excretion of CB10-277 and the glucuronide metabolite at the MTD was $0.3 \pm 0.3\%$ and $38 \pm 9\%$ (mean ± s.d.), respectively. Thirty-six patients with various tumour histologies have received 80 courses of CB10-277 by short i.v. infusion over a dose range of $80–6000\,mg\,m^{-2}$. Pharmacokinetic analyses have been performed on 46 courses. CB10-277 plasma clearance in patients ($61 \pm 25\,ml\,min^{-1}\,m^{-2}$) was biphasic with infusion times of <10 min ($t_{\frac{1}{2}\alpha} = 13 \pm 7$ min, $t_{\frac{1}{2}\beta} = 86 \pm 40$ min). Three metabolites have been identified in plasma: CB10-277 glycine conjugate, CB10-277 glucuronide conjugate and the MM. The highest MM levels were detected at the MTD where the range was 18–32 $\mu$M. The two conjugates were the major metabolites identified in the urine. Urinary excretion of CB10-277, glycine and glucuronide conjugates was $0.9 \pm 2.5\%$, $19 \pm 11\%$ and $31 \pm 15\%$, respectively. Nausea and vomiting were observed at all levels $>900\,mg\,m^{-2}$ and became dose limiting (WHO grade 3) at the human MTD of $6000\,mg\,m^{-2}$. The only other significant toxicity observed was flushing without haemodynamic consequences in 76% of patients treated with $\geqslant 2000\,mg\,m^{-2}$. Responses occurred in patients with melanoma (1 complete, 2 partial, 1 mixed/11 patients) and sarcoma (1 mixed/6 patients). These pharmacokinetic results support the conclusion that there are qualitative metabolic similarities between mice and patients following CB10-277 i.v. administration. In addition, the clinical CB10-277 antitumour spectrum appears similar to DTIC; whether it will be more useful should be determined by further trials in a DTIC sensitive tumour.

### Pharmacokinetic aspects of plasma drug concentration monitoring for prediction of etanidazole (SR 2508) toxicity

P. Workman, R. Ward, T.S. Maughan, H.F.V. Newman & N.M. Bleehen

*MRC and University Department of Clinical Oncology and Radiotherapeutics Unit, Hills Road, Cambridge CB2 2QH, UK.*

Although considerably less neurotoxic than misonidazole because of its hydrophilic character leading to rapid renal clearance and partial exclusion from the nervous system, peripheral neuropathy remains dose-limiting for the developmental hypoxic cell sensitiser etanidazole. Monitoring plasma drug concentrations by HPLC to determine the area under the curve ($AUC_{0-\infty}$) provides a quantitative method of predicting patients at risk. We have analysed data for 18 patients receiving multiple doses of $2\,g\,m^{-2}$ etanidazole to identify the optimum time-points for accurate estimation of AUC. Kinetic analysis was first carried out using time-points at 0, 15 and 30 min and 1, 2, 4, 8, 12 and 24 h. Data fitted the 2-compartment model, with mean $t_{\frac{1}{2}\alpha}$ and $t_{\frac{1}{2}\beta}$ values of 24.3 min and 5.67 h respectively, with CVs of 40 and 18%. AUC was estimated from the rate equation as $A/\alpha + B/\Delta$. The average AUC was $503\,\mu g\,ml^{-1}\,h$ with a CV of 30%. Omitting either the 0 or 24 h time points gave quite small average errors (2.5%) but individual patient errors of up to 16 and 7% respectively were seen. Leaving out both the 8 and 12 h points together gave a similar low mean error of 2.9% and a highest value of 7%. Omitting all points after 4 h gave an average error of 25% and 15/18 patients had errors greater than 10%. Failure to correct for infusion time results in an average underestimation of AUC by 5%. We conclude that omitting the 8 and 12 h points only combines maximum staff and patient convenience with accurate estimation of AUC for toxicity prediction.

### The effect of ifosfamide and its metabolites on intracellular glutathione levels *in vitro* and *in vivo*

A.T. McGown, M.J. Lind, D.G. Poppitt, J.A. Hadfield, N. Thatcher, D. Crowther & B.W. Fox

*Paterson Institute for Cancer Research, Dept of Medical Oncology, Christie Hospital, Manchester M20 9BX, UK.*

The technique of flow cytometry and the glutathione-specific fluorochrome monochlorobimane have been used to study the effect of ifosfamide and its metabolites on P388 cells *in vitro*, and also on the glutathione levels in lymphocytes of a patient undergoing an 8 h infusion with ifosfamide. Of all metabolites only 4-hydroperoxyifosfamide and chloroacetaldehyde are capable of reducing intracellular GSH. However the concentration of 4-hydroperoxyifosfamide required to reduce GSH by 50% (1 mM) was far in excess of that achievable in patients receiving the drug. In contrast chloroacetaldehyde was found to reduce GSH at levels known to be reached in patients receiving ifosfamide (100 $\mu$M).

The glutathione levels in the lymphocytes isolated from a patient undergoing an 8 hour infusion of ifosfamide show a marked decrease to about 30% of their original values. It is concluded that ifosfamide causes glutathione depletion *in vivo*, the majority of which can be accounted for by the production of chloroacetaldehyde.

**Pharmacokinetics of pyridoglutethimide, a new aromatase inhibitor**

B.P. Haynes, L.J. Griggs, M. Jarman, R.C. Coombes, P.E. Lønning & T.J. Powles

*Institute of Cancer Research, Sutton, Surrey SM2 5NG; St George's Hospital, Cranmer Terrace, London SW17; and Royal Marsden Hospital, Sutton, Surrey, UK.*

Pyridoglutethimide (PG) is an analogue of aminoglutethimide, an aromatase inhibitor currently in use for the treatment of hormone-dependent metastatic breast cancer. PG is a selective aromatase inhibitor which, unlike its parent compound, does not inhibit the desmolase enzyme complex (Foster *et al., J. Med. Chem.*, 1985, **28**, 200). Furthermore, PG lacks the CNS side-effects associated with aminoglutethimide (sedation, ataxia, anticonvulsant activity) in standard tests in mice. As part of a phase I trial the pharmacokinetics of PG have been studied in postmenopausal patients with metastatic breast cancer. Each patient received a single oral dose of PG (1000 mg) on day 0 and plasma samples were obtained at 0, 0.5, 1, 2, 4, 6, 8, 12, 15, 24, 28, 32, 36 and 48 hours after this. Further oral doses of PG (1000 mg) were then given daily for 5 days (days 2–6). After the last dose of PG plasma samples were taken at the same time-points as they were following the first dose of PG on day 0. Plasma concentrations of PG and its principal metabolite, the *n*-oxide, were measured by reversed-phase HPLC with UV detection at 254 nm.

Curvilinear plasma concentration–time profiles for PG were obtained in all patients. Fitting of these curves to the integrated Michaelis–Menten equation yielded excellent parameter estimates for $C_o$, $K_m$ and $V_{max}$ as indicated by small standard errors of determination. The mean $C_o$, $K_m$ and $V_{max}$ values after a single dose of PG were 24.74 $\mu g l^{-1}$, 1.88 $\mu g l^{-1}$ and 0.80 $\mu g l^{-1} h^{-1}$ respectively. After repeat dosing both the Km and Vmax were found to be increased (3.96 $\mu g l^{-1}$ and 1.89 $\mu g l^{-1} h^{-1}$ respectively) while there was no change in Co (24.85 $\mu g l^{-1}$). It was also observed that the AUC of PG was over 30% less and the AUC of PG *N*-oxide over 50% greater after repeat dosing compared to that after a single dose. The data obtained indicate that the pharmacokinetics of PG are best described by a non-linear system with Michaelis–Menten elimination kinetics. It would also appear that PG induces its own metabolism, a phenomenon observed with aminoglutethimide (Murray *et al., J. Clin. Pharmacol.*, 1979, **19**, 704), and that the site of induction may be the *N*-oxidation process.

**Non-renal clearance of methotrexate (MTX) in patients with bladder cancer**

S. Harland & J. Hartley

*Institute of Urology and Department of Oncology, University College, London, UK.*

There is very little information on the non-renal clearance of MTX in adult patients – a matter which may be a determinant of toxicity in patients with poor renal function. Nineteen patients with incurable bladder cancer were treated with carboplatin in combination with MTX.

MTX was given in a dose determined by body weight and glomerular filtration rate (GFR) (range: 330–680 mg) infused over 24 hours. Sampling of plasma and urine at steady state, towards the end of the infusion, permitted calculation of total and renal clearance, and by subtraction, non-renal clearance.

Mean MTX concentration at steady state was $3.0 \pm 0.4$ (s.e.) $\mu$M. Mean total clearance was 240 ml min$^{-1}$. 50% of this was renal and 50% non-renal. The renal clearance was $2.3 \pm 0.3$ fold greater than the GFR. Large variations in the non-renal clearance were seen (range 14–94% of total clearance). Non-renal clearance correlated very strongly with body weight. Thus for patients whose weight was >70 kg this value was $191 \pm 42$ ml min$^{-1}$ ($n=6$); weight <70 kg, $86 \pm 18$ ml min$^{-1}$ ($n=8$). Repeated estimations in a patient who recovered his weight loss on chemotherapy showed improvement from 91 to 192 ml min$^{-1}$. Four patients with grade 3 performance status had a mean value of $56 \pm 28$ ml min$^{-1}$. for PS 0 and 1 it was $140 \pm 23$ ml min$^{-1}$ ($n=15$).

Patients with low body weight and poor performance status in addition to a low GFR may have a compounded susceptibility to methotrexate toxicity.

**Potentiation of the anti-tumour effect of melphalan by the calcium antagonist nifedipine**

J. Godden, I.J. Stratford, J. Bremner & G.E. Adams

*MRC Radiobiology Unit, Chilton, Oxon OX11 0RD, UK.*

It has been shown that the vasoactive agent hydralazine can substantially increase the anti-tumour effect of melphalan (*Br. J. Cancer*, 1988, **58**, 122). Hydralazine is an anti-hypertensive agent effecting the peripheral vascular supply causing reduced blood flow to tumours. We report here a study on nifedipine, another clinically used vasoactive agent, for its ability to alter tumour blood flow in mice and to modulate tumour and tissue damage caused by melphalan. the KHT sarcoma implanted either s.c. or i.m. requires a dose of 6 mg kg$^{-1}$ melphalan to reduce surviving fraction of tumour cells to 0.1. In contrast when 10 mg kg$^{-1}$ nifedipine is given 15 min after melphalan the dose required to reduce survival to this level is 2 mg kg$^{-1}$. In poorly vascularised pulmonary metastases the drug combination is less effective, suggesting that nifedipine induced vascular changes associated with the larger tumours may contribute to the increased efficiency of the alkylating agent. This contention is supported by measurements of changes in tumour blood flow caused by nifedipine. However, blood flow changes by this dose of nifedipine are not sufficient to cause induction of close to 100% tumour hypoxia, which contrasts with our previous results with hydralazine, nor is there any increase in hypoxia related binding of $^3$H-misonidazole to tumour tissue. Further, nifedipine also acts by increasing melphalan induced damage to the mouse intestine. However, the DMF for this normal tissue damage is less than the increased efficacy of melphalan against the s.c. or i.m. tumours.

**Monitoring drug-induced hypoxia in murine tumours by $^{31}$P NMR**

J.F. Dunn, N. Howells, I.J. Stratford, G.E. Adams & G.K. Radda

*MRC Clinical and Biochemical Spectroscopy Unit, Dept of Biochem., Oxford and MRC Radiobiol. Unit, Chilton, Didcot, Oxon., UK.*

A knowledge of the oxidative status of tumours is important since hypoxic cells in tumours are resistant to radiation and can be refractory to a number of commonly used cytotoxic drugs. Experiments have been carried out to manipulate tumour oxygenation in mice using either the vasoactive agent hydralazine or BW12C, an agent which shifts the oxyhaemoglobin curve to the left. Changes in tumour oxygenation were measured by determining the proportion of hypoxic cells using an *in vitro–in vivo* assay of tumour response to radiation (the radiobiological hypoxic fraction). Tumour NMR spectra were measured using a vertical bore 4.2T magnet with a Bruker spectrometer, or a horizontal bore 4.7T magnet with a Varian spectrometer. Mice were anaesthetised, placed in the magnet for control spectra, removed for i.v. administration of the drug, and placed back in the magnet to follow the time-course of alteration in phosphorus containing metabolites. After administration of the vasodilator hydralazine (on KHT, RIF-1 and 16C murine tumours), we have shown that the ratios of inorganic phosphate (Pi) to ATP increase with a time course which is tumour specific. Further, the time course is similar to that observed for the radiobiological hypoxic fraction. KHT tumours in mice given BW12C show variable NMR spectra. However, in those tumours where changes are observed, the Pi/ATP ratio increases by 1.5–3 times that of the control value and the time course of the change is similar to that seen in the radiobiological hypoxic fraction. These observations indicate that substantial changes in oxidative metabolism can result from administration of BW12C and hydralazine.

**The hepatic asialoglycoprotein receptor as a target for drug delivery**

L. Seymour, P. Flanagan, K. Ulbrich & R. Duncan

*Cancer Research Campaign Laboratory, University of Keele, Staffs. ST5 5BG; Institute of Macromolecular Chemistry, Prague 6, Czechoslovakia.*

A soluble synthetic polymer (*N*-(2-hydroxypropyl)-methacrylamide), developed as a lysosomotropic drug carrier, has been tailored to interact with the hepatocyte asialoglycoprotein receptor by incorporation of galactose (5.5 wt%). Doxorubicin (DOX, 7.3 wt%) was covalently attached to the polymer carrier via a tetrapeptide spacer designed for intracellular cleavage by a lysosomal enzymes. When administered intravenously a single dose of this conjugate (containing 5 mg DOX $kg^{-1}$) shows therapeutic activity against L1210 (i.p.). The mechanism of action is unknown.

At low doses (0.05 mg DOX $kg^{-1}$) up to 48% of administered radiolabelled conjugate was taken into the liver, the amount of DOX deposited increasing (up to 23 $\mu g\,g^{-1}$) as a function of the dose administered (up to 20 mg $kg^{-1}$). At a dose of 5 mg $kg^{-1}$, 7 $\mu g$ of DOX was captured per g liver tissue (60 min) but at this time 24% of the dose remained in the circulation due to saturation of the receptor. Circulating material is rapidly excreted in the urine, however, and repeat administration of drug conjugate after 24 h produced an identical profile of blood clearance, suggesting that receptors were again fully available. In addition there was little or no IgG response against the drug conjugate (5 mg $kg^{-1}$ DOX administered thrice in adjuvants).

Although the concept of a liver depot slowly releasing drug to treat peripheral disease is still not established, we feel that this route of targeting should be clinically useful in the treatment of malignant disorders of the liver.

**Comparative toxicity of HPMA copolymer-adriamycin conjugates and free adriamycin in the rat**

T.K. Yeung, R.H. Simmonds, J.W. Hopewell, L.W. Seymour, R. Duncan & K. Ulbrich

*CRC Radiobiology Research Group, Churchill Hospital, Oxford, UK and CRC Polymer Controlled Drugs Delivery Group, University of Keele, Keele, UK.*

A rat model has been used to evaluate the general toxicity and the late cardiotoxicity of 4 mg $kg^{-1}$ adriamycin (free drug) and *N*-(2-hydroxypropyl)-methacrylamide (HPMA) copolymer conjugates containing 4 mg $kg^{-1}$ adriamycin. These were bound to the copolymer via peptide linkages which were either (i) non-biodegradable (Gly-Gly) or (ii) biodegradable (Gly-Phe-Leu-Gly) or as (ii) but with the incorporation of galactosamine into the copolymer. After i.v. administration of the drugs all animals showed a transient reduction in body weight in the first two weeks. The average maximum reductions in body weight in the three groups of animals receiving HPMA–copolymer conjugates were not significantly different from each other and were significantly less than those measured in animals receiving free adriamycin ($P < 0.01$). Over the 12-week period of this study, deaths were only observed in animals receiving free adriamycin. Cardiac output (COP) was measured using an external counting technique. In all animals receiving the conjugates the COP was not significantly different from that measured in age-matched control animals ($P > 0.05$). However, these animals did show a slight (~9%) but significant increase in heart rate from 8 weeks after drug administration ($P < 0.01$). The COP measured in surviving animals receiving free adriamycin showed a ~70% reduction, with a reduced heart rate, after 4 weeks. This study demonstrated that conjugates of adriamycin HPMA–copolymer significantly reduced its general toxicity and increased animal survival. The present results suggest a greater than 4-fold reduction in cardiotoxicity when adriamycin was administered as a HPMA–copolymer conjugate. Further studies are planned to evaluate the dose-related cardiotoxicity of the agents.

**Antitumour activity of *N*-(2-hydroxypropyl)Methacrylamide copolymers containing anthracyclines**

R. Duncan, I.C. Hume, K.B. O'Hare, J. Strohalm, J. Kopecek & K. Ulbrich

*Cancer Research Campaign Laboratory, University of Keele and Institute of Macromol. Chem., Prague, Czechoslovakia.*

Biocompatible soluble synthetic polymers show excellent potential as carriers of anticancer agents for controlled and targeted drug delivery. HPMA copolymer conjugates have been synthesized to contain daunorubicin (DNR) and doxorubicin (DOX), the drugs being bound to the polymer by lysosomally degradable peptide side-chains. The conjugates have a $Mw \sim 20{,}000$, $Mw/Mn \sim 1.2$ and drug loading 6–10 wt%. The antitumour activity of such conjugates has been evaluated against L1210 leukaemia and B16 melanoma in $DBA_2$ or C57/6J mice (i.p. or s.c.) and Walker sarcoma in rats. Conjugates were administered i.p. or i.v. (range 5–100 mg $kg^{-1}$ in respect of drug bound). When tested against L1210, free drug (DNR, DOX) always showed lower activity than polymeric derivative over a wide concentration range and using a variety of protocols for administration. Conjugates were substantially less toxic ($LD_{50}$ 50–75 mg $kg^{-1}$) and always more active (up to 4/5 surviving at 50 days) than free drug (no survivors). Preliminary experiments against a solid tumour model (Walker) showed activity of DOX–HPMA copolymer conjugate (single dose, day 1, 5 mg kg$^{-1}$, i.p.) at day 14 giving a 70% reduction in tumour size. The above conjugates show superior activity due to controlled delivery of drug and perhaps by passive tumouritropism. Conjugates have also been synthesised to contain carbohydrates, sugars or peptides/proteins to promote tumour-specific capture. For example, conjugates containing melanocyte stimulating hormone (MSH) and DOX were administered (5 mg kg$^{-1}$ i.p.) to mice bearing B16 melanoma (i.p. or s.c.). The conjugate containing MSH was more active than than without (3/5 of survivors at day 50: 0/5 survivors), but in this case free drug at equivalent dose also displayed activity against this tumour model (2/5 survivors).

### Activity of HPMA copolymers containing daunomycin (DNM) against a rat tumour model

J. Cassidy, R. Duncan, G.J. Morrison, J. Strohalm, D. Plocova, J. Kopecek & S.B. Kaye

*CRC Dept of Medical Oncology, Glasgow, UK; CRC Polymer Controlled Drug Delivery Group, Univ. of Keele, UK; and Institute of Macromolecular Chemistry, Prague, Czechoslovakia.*

Polymeric drug carriers based on water soluble non-immunogenic copolymers of *N*-(2-hydroxypropyl)methacrylamide (HPMA) have been developed in recent years by Duncan *et al.* This group have shown that it is possible covalently to bind drugs to these carriers in such a way as to be stable in the bloodstream, but then to be cleaved inside the cell by lysosomal cysteine proteinases. We present data to show modification of pharmacokinetics and a degree of 'targeting' in a rat tumour model, using HPMA copolymers containing DNM.

5 mg kg$^{-1}$ of free DNM or HPMA bound DNM was administered i.v. to Wistar rats bearing subcutaneous implants of Walker 256 tumour. At various times up to 24 hours later animals were killed and tissues removed for determination of free DNM by means of an HPLC assay. The polymer bound drug was found in the tumour at greater concentration than the free drug at all time points. Tumour AUC was increased approximately 4-fold.

Five animals in four groups were given; saline as control, 5 mg kg$^{-1}$ of free DNM, the same dose of DNM bound to HPMA via a biodegradable 'spacer' or the same dose of DNM bound to HPMA by a non-biodegradable linkage. The injections were performed on the same day as subcutaneous implantation of 0.5 cm cubes of Walker tumour. Subsequent tumour growth was measured by calipers every 2–3 days. The only group with a significant growth delay were the animals given the biodegradable polymer preparation. In fact 4/5 animals in this group were long-term survivors (>180 days), compared with only 1/5 with free DNM. In summary, this carrier favourably influences pharmacokinetics and efficacy in this model system. Further studies are planned with other models before clinical trials.

### Will biodegradable emboli enhance regional chemotherapy for hepatic metastases?

D. Chang, S.A. Jenkins, D.M. Nott & T. Cooke

*Department of Surgery, University of Liverpool, Royal Liverpool Hospital, Liverpool, UK.*

Embolisation by degradable starch microspheres (DSM) with regional chemotherapy to treat hepatic metastases is thought to result in intra-tumour vascular stasis. As the true mechanism is unknown we have studied this using a hypovascular hepatic tumour derived from the intraportal injection of SN fibrosarcoma cells.

Intrahepatic distribution of $^{99}$Tc methylene diphosphonate (MDP), representing a cytotoxic drug, injected via the hepatic artery was measured before and after hepatic artery clamping or mixed with DSM with and without portal vein clamping. The distribution of 15 $\mu$m $^{57}$Co microspheres was also measured with or without co-administration with DSM.

Administration of MDP alone resulted in a tumour to liver ratio of $0.79 \pm 0.07$: $0.61 \pm 0.04$. Co-injection with DSM significantly increased this 14 fold to $11.11 \pm 1.08$: $-0.69 \pm 0.08$ ($P<0.001$, Mann–Whitney) but hepatic arterial clamping had minimal effect on MDP retention with a tumour to liver ratio of $1.19 \pm 0.34$: $0.53 \pm 0.35$. Following portal vein occlusion DSM increased retention in liver but less so in tumour ($4.35 \pm 3.63$: $2.21 \pm 1.16$). The tumour to liver ratio of 1:2 with $^{57}$Co microspheres was reversed using DSM.

These results demonstrate that DSM enhances the retention of a low molecular weight marker in hepatic tumour, not by causing intratumour stasis, but by reducing arterial flow to normal liver thereby allowing selective uptake by tumour.

### The effects of vasopressin on hepatic and tumour haemodynamics in an experimental model of liver tumour

D. Hemingway, D.M. Nott, D. Chang, S.A. Jenkins, S. Grime & T. Cooke

*Department of Surgery, University of Liverpool, Royal Liverpool Hospital, Liverpool, UK.*

The ineffectiveness of regional chemotherapy in potentiating the delivery of cytotoxic drugs to hepatic metastases may be related to the avascular nature of most liver tumours. Since vasopressin (AVP) is known to alter extra- and intra-hepatic haemodynamics, the aim of this study was to investigate its effects on blood flow to normal liver tissue and hepatic metastases. Systemic, splanchnic, hepatic and tumour haemodynamics were measured before and after a 10 minute infusion of 0.1 mU kg$^{-1}$ min$^{-1}$ AVP in rats with liver tumour derived from intraportal administration of HSN sarcoma cells using a dual microsphere technique. AVP infusion was associated with significant increase in arterial blood pressure, portal venous inflow (mean ± s.e.m. $2.03 \pm 0.3$ to $6.07 \pm 1.17$ ml min$^{-1}$; $P<0.01$ Student's $t$ test) and the tumour to liver blood flow ratio (0.62:1 ± 0.24 to 1.31:1 ± 0.3; $P<0.05$). Heart rate was reduced but hepatic arterial flow was not significantly changed by AVP administration. These results indicate that the alterations in hepatic haemodynamics effected by low dose AVP infusion increase blood flow to liver tumour and suggest that the drug may potentiate the delivery of cytotoxics to hepatic metastases.

### Immunogold visualisation of adriamycin in a human breast cancer cell line (2R 75)

W. Aherne, H. Henneberry & S. Stallard

*Dept of Biochemistry, University of Surrey, Guildford GU2 5XH; and Dept of Medical Oncology, University of Glasgow, Glasgow, UK.*

The mechanism by which anthracyclines exert their effects on normal and neoplastic cells is still largely undefined. Information on their cellular distribution at different concentrations may provide important evidence on sites and mechanisms of action. Silver enhanced immunogold (IGSS) techniques have been used to visualise the distribution of adriamycin (ADR) in a human breast carcinoma cell line (2R75). Cells were cultured until almost confluent and treated with 0–1 $\mu$g ml$^{-1}$ ADR for 24 hours. The cells were resuspended in drug free medium for 24 hours, trypsinised and harvested. Aliquots of each cell suspension were pipetted onto albumin-coated slides, air dried and freeze permeabilised on dry ice. A rabbit polyclonal antibody, produced against an ADR-BSA conjugate was purified on a controlled pore glass column to which BSA had been coupled. This procedure eliminated nonspecific binding to proteins. The slides were incubated with ADR antibody overnight, stained with gold labelled second antibody (Intense M, Janssen Life Sciences) and counterstained with eosin. The intensity and extent of immunostaining corresponded to ADR concentration in the cells measured with a specific ADR radioimmunoassay (0–721 pg $\mu$g$^{-1}$ protein). At low concentrations staining was confined to the cell membrane but at higher concentrations both the cell membranes and intracellular components were stained. Using IGSS immunocytochemistry it is possible to visualise the distribution of ADR in cells grown in culture and the technique has the potential of being used with electron microscopy for subcellular investigations. Supported by the CRC.

**Concentration and time-independent factors in the determination of cytotoxicity?**

S.M. Crawford, R.M. Phillips, J.A. Double & M.C. Bibby

*Clinical Oncology Unit, University of Bradford, BD7 1DP, West Yorkshire, UK.*

The anti-tumour effect of a cytotoxic agent is accepted as being determined by the area under the plasma concentration curve in *in vivo* studies, represented by the product of concentration (C) and exposure time (t) in *in vitro* work. Antimetabolites are regarded as potential exceptions to this because enzyme inhibition by drugs used clinically is usually reversible. The aim of this study was to assess *in vitro* whether or not long exposures to low drug concentrations were more toxic than short exposures to high drug concentrations whilst maintaining the same overall drug C x t exposure. The cell line used in this study was derived from an ascitic murine adenocarcinoma of the colon (MAC 15A) tumour and chemosensitivity *in vitro* was assessed using a modified clonogenic assay. ThioTEPA caused little variation in cell survival following a 3, 6 and 24 hour exposure with $ID_{70}$ values ranging from 20 to 15 $\mu$g h ml$^{-1}$. One hour exposure proved to be less active ($ID_{70}$ = 25 $\mu$g h ml$^{-1}$). Methotrexate was ineffective at all drug concentrations following a 1 hour drug exposure. A 3, 6 and 24 hour exposure produced dose–response curves with an initial exponential phase followed by a distinct plateau phase. Cell kill was strongly related to the duration of drug exposure. Flavone acetic acid induced exponential dose response curves. Long exposures to low drug concentrations were more toxic with $ID_{70}$ values for 1, 6, 24, 48 and 72 hour drug exposures of 8, 10, 7, 2.5 and 1.25 mg h ml$^{-1}$ respectively. Preliminary data suggest that adriamycin shows a similar but much smaller time-dependent effect against MAC 26 cells. This suggests that for drugs other than alkylating agents clinical schedules ensuring prolonged exposure should be investigated and that studies of tumour penetration by cytotoxics should address the dynamics of this process.

**Blood transfusion, recurrence of laryngeal carcinoma and AgNORs**

T. Alun-Jones, P.J. Clarke & J. Hill

*Department of Otolaryngology, Radcliffe Infirmary, Oxford and Nuffield Department Surgery, Oxford, UK.*

Blood transfusion induced immunosuppression may have a detrimental effect on prognosis in patients undergoing surgery for malignant disease but retrospective clinical trials have yielded conflicting results. In those studies which have found a positive correlation between transfusion and prognosis, it is possible that transfusion is only an epiphenomenon, and is a marker of patients with an intrinsically bad prognosis. In order to investigate this possibility, 69 patients who had a laryngectomy for carcinoma of the larynx were studied. Patients were excluded if they had metastatic disease at the time of surgery (either local or distant). 38 had received a peroperative transfusion and 31 had not. The groups were comparable for age, sex, site of tumour, stage, operating time and preoperative haemoglobin. At 5 years, survival in the transfused group was 44.7% compared with 90.3% in those patients who were not transfused ($P$<0.001, $\chi^2$ test). Blood transfusion was found to be the only factor of importance in determining outcome when prognostic factors were included as covariates in a multiple regression analysis ($P$<0.01). the paraffin sections of the tumours were then stained for the presence of AgNOR bodies. These are extranuclear collections of DNA, and the number of AgNORs per cell has recently been postulated as a measure of biological tumour aggression. In a preliminary study of the laryngeal tumours from our study there was no difference in AgNOR scores between transfused and nontransfused patients. This implied that the deleterious effect of transfusion in this study could not be explained by a difference in either known prognostic variables or by a marker of tumour aggression.

**Pregnancy outcomes in childhood cancer survivors: probable effects of abdominal irradiation**

R.A. Smith & M.M. Hawkins

*Childhood Cancer Research Group, Radcliffe Infirmary, Woodstock Road, Oxford, UK.*

A postal survey of 2,083 general practitioners of childhood cancer survivors of reproductive age has revealed that females who were directly abdominally irradiated (exposed), particularly for Wilm's tumour, have an increased risk of several untoward pregnancy outcomes as compared with female survivors of the same types of tumour not directly abdominally irradiated (unexposed). Among female survivors 22% and 41% of those exposed and unexposed, respectively, have children. The corresponding percentages of first pregnancies reported as ending in spontaneous abortion were 9/40 = 22% and 11/174 = 6%. The mean birthweight of first singleton children born to exposed mothers was over 300 g less than the corresponding value for unexposed mothers. We conclude that radiation is probably involved in the mechanism producing these effects but how it does so is uncertain. There are four hypotheses to explain this association: (a) radiation induced genetic damage; (b) uterine abnormalities; (c) lack of uterine distensibility; (d) late effects of radiation on the vasculature of the uterus. With the aid of modern ultrasound and pathological techniques it should prove possible to test these hypotheses. A register of such women is to be set up with the aim of appropriately monitoring these women and their pregnancies and to obtain the placentae, and placental site biopsies (where appropriate)

for histological examination. The findings have implications for counselling survivors, monitoring their pregnancies and treating future patients.

**Which smokers develop lung cancer?**

D.J. Hole, C.R. Gillis & V.M. Hawthorne

*West of Scotland Cancer Surveillance Unit, Glasgow, UK and Department of Epidemiology, Univ. of Michigan, USA.*

Cigarette smoking is a major risk factor for both lung cancer and ischaemic heart disease. What is not clear is whether other characteristics differentiate between these two diseases. This paper examines the role of respiratory symptoms, cholesterol and body mass index using data collected in a prospective general population cohort study of 15,399 men and women aged 45–64 resident in urban West of Scotland, an area with the highest lung cancer incidence in the world. 2,397 deaths and 1,594 cancer cases have occurred in the 12 years of follow-up. Low cholesterol, low body mass index and the presence of respiratory symptoms are all significant independent factors in identifying a high risk group for lung cancer. The relationship between low cholesterol and high lung cancer risk is steepest for heavy smokers (>15 cigarettes per day), less steep for light and ex-smokers and non-significant for those who have never smoked. In addition, a similar relationship holds for low cholesterol and other smoking-related cancers. These relationships remain even when cancer cases diagnosed within the first four years following screening are excluded. Further, there is no evidence to suggest that the findings are a result of individuals with high cholesterol and high body mass index dying first from ischaemic heart disease.

**Stage II melanoma in the West of Scotland: prognostic indicators for survival and effects of adjuvant chemotherapy**

D.M. Tillman, T.C. Aitchison & R.M. MacKie

*Departments of Dermatology & Statistics, University of Glasgow, UK.*

We have carried out a retrospective analysis of patients undergoing therapeutic lymph node dissection for stage IIA melanoma at the regional plastic surgery centre between 1976 and 1985. There were 142 patients (68 M, 74 F), mean age 52 yr (range 18–87). Features of the primary tumour were as follows. (1) Site: head and neck 15.5%, trunk 20.4%, lower limb 47.9%, upper limb 16.2%. (2) Histogenetic type ($n=120$): nodular 35.9%, superficial spreading 47.5%, acral lentiginous 8.3%, lentigo maligna melanoma 5%, unclassifiable 3.3%. (3) Breslow thickness ($n=110$): 0–1.49 mm 11%, 1.5–3.49 mm 44.5%, $\geqslant$3.5 mm 44.5%. (4) Clark level ($n=118$): II 2.5%, III 15.3%, IV 67.8%, V 14.4%.

The disease-free interval between excision of the primary tumour and Stage II disease was 0 in 15%, <1 yr in 41.5%, <2 yr in 23.2%, 2–4 yr in 9.1% and >4 yr in 11.2%. In 35.3% 1 node, 21.8% 2 nodes, 16.8% 3 nodes, and 26.1% 4 or more nodes were involved ($n=119$).

91 patients (64.1%) received no adjuvant therapy, 24 (16.9%) had 2 courses of adjuvant BELD (bleomycin, vindesine, lomustine, DTIC) and 27 patients (19%) other adjuvant monotherapy. The overall 5-year survival after lymphadenectomy (life table analysis) was 26%. Although there was a trend towards improved survival in both groups receiving adjuvant therapy, this was not significant. Using a proportional hazards model with stepwise variable selection, overall survival was significantly related to the number of involved nodes and the age of the patient.

This study confirms the poor prognosis of patients with stage II melanoma and that the extent of nodal metastatic disease is the main determinant of survival. It also demonstrates the limited value of systemic adjuvant therapy and provides a database for the design of new treatment strategies.

**Cancer of the stomach: is it declining?**

J. Powell & C.C. McConkey

*Regional Cancer Registry, Q.E. Medical Centre, Birmingham B15 2TH, UK.*

The precise reasons for the well documented and world-wide decline in stomach cancer have never been proven. More recently a few centres have reported an increase.

Data in the West Midlands Regional Cancer Registry include details of both subsite and histology. An analysis of the period 1962–1981 was undertaken as part of a much larger study. Age standardised incidence rates were used to allow for the ageing population. The overall decrease in stomach cancer was confirmed, but focusing on specified sites within the stomach showed a considerable increase in adenocarcinoma of the cardia (from 0.75 to 2.01 per 100,000) and smaller increases in the other sites with the exception of the pyloric antrum which remained relatively constant (at about 2.6). Unspecified sites have decreased in number but more precise localisation is unlikely to be the main reason for the changes observed since increases were also observed in adenocarcinoma of the oesophagus.

The aetiological implications are intriguing but there are also implications for patient management since lesions of the cardia have the worst prognosis. Further, the above opposing trends could not be demonstrated if only mortality data is studied.

**Analysis of prognostic factors for survival in soft tissue sarcomata**

J.N. El-Jabbour, K.M. McLaren, G.R. Kerr, S.S. Akhtar, A. Rodger, J.F. Smyth & R.C.F. Leonard

*University Department of Pathology, Teviot Place, Edinburgh EH8 9AG; and Clinical Oncology Department, Western General Hospital, Crewe Road, Edinburgh EH4 2XU, UK.*

Histological review of 223 cases referred to the Clinical Oncology Department, Edinburgh in a 10 year period with a diagnosis of sarcoma or Mullerian tumour confirmed the diagnosis of sarcoma in 163 patients. Since 33 of these were not referred at initial diagnosis but presented to us as local recurrence (21), metastases (5) or both (7), they were excluded from the survival analysis to minimise the effect of referral bias. Uni- and multivariate analyses were performed to detect significant prognostic factors on the remaining 130 patients.

The five-year survival was 21.7%. By univariate analysis, the following factors were of prognostic significance: radiotherapy ($P<0.0001$), surgical treatment ($P<0.0001$), site ($P=0.001$), mitotic activity ($P=0.001$), grade ($P=0.005$) and histological type ($P=0.039$). Necrosis ($P=0.354$), size ($P=0.409$), age ($P=0.58$) and sex ($P=0.17$) were not.

The multivariate analysis on 68/130 patients with known size revealed the following independent prognostic factors: surgical treatment ($P=0.0001$), radiotherapy ($P=0.0031$)

and necrosis ($P=0.0090$). When size was not among the variables analysed (105/130 cases) the independent prognostic parameters were: surgical treatment ($P<0.0001$), necrosis ($P=0.0006$), radiotherapy ($P=0.0096$) and mitotic activity ($P<0.0337$).

These data will be compared with those from other major series.

### Determination of prognosis in advanced colorectal cancer

K. Moran, T. Cooke, G. Forster, P. Gillen, S. Sheehan, P. Dervan & J.M. Fitzpatrick

*Departments of Surgery and Pathology, Mater Hospital, Dublin, Ireland, and Royal Liverpool Hospital, Liverpool, UK.*

Conventional clinical and pathological prognostic parameters are inaccurate in colorectal cancer. One-third of patients with Dukes' B die with 1/3 of those with Dukes' C surviving long-term, irrespective of histological differentiation. We studied the prognostic value of NORs and ploidy status and their correlation with established prognostic parameters in advanced colorectal cancer. Fifty-one patients aged 35–81 (mean 61.6 years) with Dukes' C tumours (8 with liver metastases) were studied. All were followed for a minimum of 5 years. Sections from primary tumours (1°) and lymph node metastases (LN) were stained for NORs. Ploidy status was determined for primary tumours in 40 patients using flow cytometry. NOR and ploidy status were correlated with age, sex, histological differentiation, presence of liver metastases and survival time and the independent significance of prognostic variables was determined using Cox's multivariate regression analysis. NORs and ploidy values did not correlate with age or sex. Sixteen patients who survived 5 years had lower NOR counts than non-survivors ($P<0.05$) Mann–Whitney U test. Survivors had a lower percentage of aneuploid tumours. NORs were the most important individual variable for predicting survival ($P<0.05$). Ploidy values correlated with histological differentiation and liver metastases.

| | *Survivors* | *(1° LN)* | *Non-survivors* | *(1° LN)* |
|---|---|---|---|---|
| NOR (median, range) | 12 (8–15) | 11 (8–15) | 17 (14–25) | 18 (13–25) |
| Ploidy (% aneuploid) | 40% | | 57% | |

In conclusion, NORs were more accurate and ploidy values were as accurate as conventional clinical and pathological parameters in predicting 5-year survival in advanced colorectal cancer.

### Will DNA ploidy predict response to chemotherapy in end-stage squamous carcinoma of the head and neck?

L.D. Cooke, T.G. Cooke, G. Forster, T.R. Helliwell, D. Spiller & P.M. Stell

*Departments of Otorhinolaryngology, Surgery, Pathology and Radiation Biology, Univ. of Liverpool, UK.*

In two phase III trials we have shown that cisplatinum significantly prolongs survival in patients with end stage squamous carcinoma of the head and neck region. Factors such as performance status, age, site, nodal status and serum albumin are prognostic but are not significantly clear cut to define which patient should be treated. In an attempt to improve on this we have measured tumour cellular DNA in this same group.

We studied tumours from 41 untreated and 52 patients receiving cisplatinum as part of a prospectively randomised trial. 50 $\mu$m sections of tumour were enzymatically disaggregated, treated with RNAase, and the DNA stained with propidium iodide. Following analysis by flow cytometry they were classified as diploid or aneuploid.

Fifty-four per cent of all tumours were aneuploid and 46% diploid, with similar proportions in both treated (57–43%) and untreated patients (51–49%). Survival for untreated patients with diploid tumours of 68 days was increased to 128 days after chemotherapy; this difference was not significant ($\chi^2=1.91$). However, the survival of aneuploid tumours increased four-fold from 55 to 224 days ($\chi^2=10.29$ $P<0.001$).

This study suggests that cisplatinum chemotherapy in end stage head and neck cancer should be restricted to patients with aneuploid tumours.

### Steroid receptors and enzyme activity in human breast carcinoma

V. Ramalingam & P. Govindarajulu

*Dept of Endocrinology, P.G. Institute of Basic Medical Sciences, Madras 600 113, Dept. Zoology, Tagore Arts College, Pondicherry 605 008, India.*

The potential markers of oestrogen action in breast cancer is of greatest importance to improve the selection of patients for hormonal therapy. Since the activities of lactate dehydrogenase (LDH) and glucose-6-phosphate dehydrogenase (G6PDH) have been reported to be oestrogen responsive in reproductive tissues, an attempt has been made to evaluate oestrogen receptor (ER), progesterone receptor (PR), LDH and G6PDH in the cytosol of 160 human breast cancer tissues. 71% and 64% of the subjects were found to be ER positive and PR positive (>5 fmol mg protein$^{-1}$) respectively. More than 80% of the cases showed increased activities of LDH and G6PDH when compared to the normal tissues. The enzyme activities were similar in ER positive cases and ER negative cases though they were observed with elevated activity. However, both the enzyme activities were significantly higher in the PR positive cases when compared to PR negative cases. PR has been shown as a post-nuclear marker of oestrogen action. From the results of this study, it may be suggested that LDH and G6PDH could be considered as sensitive markers of functional ER sites and it may provide a better monitor for the selection of hormone dependent cancer.

### The relationship between myeloma-mediated inhibition of osteoblast-like cells and TNF

C.E. Evans, C.S.B. Galasko & C. Ward

*University of Manchester, Dept of Orthopaedic Surgery, Hope Hospital, Eccles Old Road, Salford M6 8HD, UK.*

We have previously reported that the bone-resorbing tumour myeloma will inhibit the proliferation and DNA synthesis of human osteoblast-like cells (BDC) *in vitro*. Further experiments have demonstrated that myeloma cells from other sources also have this effect. Thus, having established that this is a reproducible effect and common feature of this tumour, we are now attempting to find the substances responsible. We report here preliminary results for this project. Using the cytotoxicity test for TNF, we have found this cytokine to be present in medium conditioned by the myeloma cells (MCM), at a concentration of 35 U ml$^{-1}$. We then incubated our BDC with pure TNF-$\beta$ at the same concentration as that in MCM. Proliferation of the BDC was inhibited by a mean of 39%. Incubation of the BDC with TNF-$\beta$ at a range of concentrations showed that this effect was dose-dependent. As the cytokine concentration increased, the inhibition of the BDC growth increased. These results confirm those found with MCM itself. A plateau was reached at 35U/ml, after which there was no further increase in inhibition. TNF-$\beta$ also had a dose-dependent effect on DNA synthesis, as with increasing concentrations of TNF-$\beta$, increasing stimulation was seen. No plateau was reached, however. These results are dissimilar to those seen with MCM. We are now attempting to isolate TNF-$\beta$ from the MCM. Initial purification work has yielded some fractions with similar effects to TNF-$\beta$. Our results suggest that more than one factor is involved in myeloma mediated osteoblast inhibition.

### Comparative effects of two gastrin receptor (GR) antagonists on the growth of gastrin responsive gut tumours

S.A. Watson, L.G. Durrant & D.L. Morris

*Cancer Research Campaign Laboratories, University of Nottingham, NG7 2RD, and Dept of Surgery, University Hospital, Nottingham NG7 2UH, UK.*

The effect of 2 GR antagonists (Proglumide and CR1409, Rotta) on the growth and gastrin response of gut tumour cells was investigated.

For a comparison of GR binding, a GR positive tumour cell line was used (AR42J, rata pancreatic). The concentrations inducing 50% inhibition of binding ($IC_{50}$) of $^{125}$I-gastrin-17 (G-17) to GR was $5\times10^{-10}$M for G-17 alone, $3\times10^{-6}$M for CR1409 and $>10^{-4}$M for proglumide.

Basal growth of a human gastric tumour cell line, MKN45G which produces its own gastrin (30 ng l$^{-1}$ day$^{-1}$ as measured by an RIA) was inhibited by CR1409 to 30% of control values as measured by $^{75}$Se-selenomethionine uptake at a concentration 10 times the $IC_{50}$. The compound had no effect on cell viability. Proglumide (up to $10^{-3}$M) had no effect on basal growth.

AR42J cell growth is increased by G-17 (as measured by $^{75}$Se-selenomethionine uptake). Proglumide ($3\times10^{-4}$M) reduced the growth response to gastrin at $5\times10^{-11}$M. G-17 (150–100% of control) but not at $10^{-10}$M G-17 (280% of control). GR1409 ($3\times10^{-6}$M) reduced the growth response at both G-17 concentrations; $5\times10^{-11}$, 150–100% control, $10^{-10}$M, 280–130% of control.

Antagonists of GR can reduce both gastrin-stimulated gut tumour growth and basal growth (if gastrin is involved in autocrine/paracrine growth stimulation) and may therefore be of great therapeutic benefit.

### Epidermal growth factor receptors in cervical cancer

M. Harding, J. McIntosh, F. Rinaldi, R.P. Symonds, N. Reed, T. Habeshaw & R. Leake

*Departments of Oncology and Biochemistry, University of Glasgow, UK.*

Epidermal growth factor receptor (EGFR), a transmembrane glycoprotein is present in large amounts on many squamous carcinomas and a cervical cancer cell line (A431). EGFR has been measured in 50 non-pretreated cervical tumours (FIGO stages I, $n=9$, II $n=15$, III $n=19$ and IV $n=7$) by radioligand binding alone and in the presence of an excess of non-labelled ligand, and data subjected to Scatchard analysis. Most tumours (70%) had both high (mean KD 3.1 nmol l$^{-1}$; range 0.3–9.9) and low (mean KD 136 nmol l$^{-1}$; range 16–500) affinity binding sites, although 15 tumours had a single binding site (mean KD 5.7 nmol l$^{-1}$; range 1.1–13.7). Binding capacity expressed as fmol per mg membrane protein was 1.9 (range 0.4–5.9), 10 (range 0.8–34) and 3.2 (range 0.7–10.8) respectively: expressed as fmol per $\mu$g DNA the values were 7.5 (range 1–23), 48 (range 5–288) and 10.3 (range 2–28). There was no apparent association between the binding capacity of the high affinity or sole receptor and tumour stage. The majority (78%) of tumours were oestrogen receptor negative ($<20$ fmol per mg protein) and although this group included tumours with the highest levels of high affinity receptor, the mean values were similar (2.33 *vs* 2.3 fmol per mg membrane protein; 8.83 *vs* 6.6 fmol per $\mu$g DNA). Thus the inverse relationship between oestrogen and EGF receptors reported in breast cancer is not evident in cervical carcinoma. Median follow up is 12 months and 4 pts have died: there is no correlation, as yet, between survival and EGFR.

### Nature of mitogenic activity in extracts of colon carcinoma

J. Sweetenham, A. Richter, D. Davies & P. Alexander

*CRC Medical Oncology Unit, Centre Block CF99, Southampton General Hospital, Southampton S09 4XY, UK.*

Ten of ten surgical specimens of carcinoma were found to stain in frozen section with monoclonal antibodies directed against either human EGF or TGF$\alpha$. In the majority both the carcinoma cells (identified by staining for cytokeratin) and the macrophages (identified with a monoclonal antibody directed against CD11C) stained for either hEGF or TGF$\alpha$ or both. Acid as well as detergent extracts from these tumours were prepared and fractionated by anion chromatography. The fractions were analysed for mitogenic activity of density inhibited human fibroblasts and by ELISA for hEGF. An antibody against the EGF receptor blocked mitogenic activity induced by either EGF or TGF$\alpha$ and was used to identify TGF$\alpha$ activity after removal of hEGF by immunoabsorption. Evidence was found that the mitogenicity in some fractions was produced by very low concentrations of hEGF which were below detection by ELISA, due to potentiation by TGF$\beta$. However, the principal mitogenic activity was not due either to hEGF or TGF$\alpha$, but was fibroblast growth factor-like as it was absorbed by heparin. We cannot determine if this FGF-like activity within the tumour is produced by the cancer cells, by stromal components or by infiltrating leukocytes.

### Effects of bryostatins (BRYOs) on protein kinase C (PKC) in A549 human lung carcinoma cells

T.D. Bradshaw, I.L. Dale, A. Gescher & G.R. Pettit

*CRC Experimental Chemotherapy Group, Pharmaceutical Sciences Institute, Aston University, Birmingham B4 7ET, UK; and Cancer Research Institute, Arizona State University, Tempe, USA.*

BRYOs are macrocyclic lactones isolated from the marine bryozoan *Bugula neritina* which possess antineoplastic activity against the murine P388 lymphoma. Like the tumour promoter 12-*O*-tetradecanoylphorbol-13-acetate (TPA), the BRYOs are activators of PKC. At nanomolar concentrations the BRYOs mimic some effects of TPA, for example inhibition of growth of A549 cells, but at higher concentrations

BRYOs 1 and 2 antagonise TPA. We have tested the hypothesis that the concentration-dependent biphasic effect of the BRYOs is correlated with their ability to interact with PKC. BRYOs 1, 2, 4 and 5 competed with $^3$H-labelled phorbol dibutyrate ($^3$H-PDBu) for specific receptor sites in intact cells. On incubation for 30 min at 37°C BRYOs 1, 2, 4 and 5 at lnM inhibited $^3$H-PDBu binding by $28 \pm 2\%$, $18 \pm 7\%$, $29 \pm 7\%$ and $62 \pm 10\%$ respectively, and at 100 nm by $74 \pm 3\%$, $46 \pm 6\%$, $73 \pm 3\%$ and $78 \pm 4\%$ respectively (mean ± s.d., $n = 8$). PKC activity in the cytosolic and particulate fractions of the cells was quantified after partial purification by non-denaturing PAGE. On incubation for 1 h, 10 nM BRYO 1 induced translocation of 31% of PKC activity from the cytosol to the membrane. After 5 h, 47% of enzyme activity was down-regulated. Treatment with 1 μM BRYO 1 caused a more rapid down-regulation; after 1 h, 65% ($n = 2$) of PKC activity had disappeared. The results show that the BRYOs possess high affinity for the phorbol receptor and modulate PKC location and activity in a manner similar to TPA, which suggests that their ability to antagonise TPA-induced growth inhibition does not involve PKC or is mediated by a PKC isoform insensitive to TPA.

### Specific biochemical or less-specific biophysical action of ether lipid SRI 62-834 on tumour cell membranes *in vitro*?

C. Dive, M.G. Thompson, J.A. Hickman, J.V. Watson & P. Workman

*Pharmaceutical Sciences Institute, Aston University, Birmingham B4 7ET, and MRC Clinical Oncology Unit, Cambridge, UK.*

SRI 62-834 is a novel analogue of the phospholipid platelet activating factor currently undergoing preclinical evaluation as a membrane-directed antitumour agent. We previously showed that it modulates membrane permeability to a fluorescein derivative BCECF and propidium (P) by flow cytometry (FCM), and causes non-toxic elevation of cellular calcium ($Ca^{2+}$i) by fluorimetry using fluorescent probe Quin-2. FCM offers unique advantages of both twin-probe analysis and individual subpopulation identification. Here we report simultaneous measurement of $Ca^{2+}$i and permeability to P in drug-treated EMT6 mouse mammary tumour cells by FCM. Cells ($10^6$ ml$^{-1}$ in medium) were preloaded with Quin-2 for 1 h via its lipophilic ester (20 μM), washed and resuspended in $Ca^{2+}$ buffer (pH 7.2) before addition of propidium iodide (20 μg ml$^{-1}$) and SRI 62-834 (1–80 μM). UV laser excitation was used and blue (460–510 nm, Quin-2) and red fluorescence (>630 nm, P) were immediately analysed together with light scatter and time. After 8 min the % of cells permeable to P increased from 2% at 1 μM to 80% at 80 μM SRI 62-834. Blue fluorescence in this subpopulation was minimal. For P-impermeable cells a time- and concentration-dependent rise in blue fluorescence was followed by a decrease. Importantly, at only 1 μM drug, $Ca^{2+}$i was elevated for 20 min before falling to control levels at 35 min. At this concentration no cytotoxicity was seen by MTT assay. These results indicate a specific effect on cell membranes resulting in $Ca^{2+}$i elevation at low drug concentrations (<15 μM), with more dramatic, possibly biophysical damage allowing influx of large charged molecules at higher doses. They also support the possibility of a selective therapeutic effect against particular cell types.

### Investigation of DNA/topoisomerase II interactions during retinoic acid induced neutrophil-granulocyte differentiation: evidence for DNA site-specificity

R.J. Anderson, J.M. Cunningham, D. Fisher & G.E. Francis

*Molecular Cell Pathology Unit, Royal Free Hospital School of Medicine, London NW3 2PF, UK.*

We have shown that DNA breakage/reunion events, mediated by topoisomerase II, are necessary and very early changes during retinoic acid induced differentiation of haemopoietic cells (Francis *et al.*, *Leukaemia*, 1987, **1**, 653). Although a method (Trask *et al.*, *EMBO J.*, 1984, **3**, 671) exists for purifying DNA/topoisomerase complexes, this is most efficient for DNA sites where many enzyme molecules are attached and thus favours the recovery of replication fork DNA (where topoisomerase II operates, possible to separate daughter chromatids). DNA cleavage sites in the regulatory sequences of known genes are isolated or in small clusters, implying that this method may be unsuitable for examination of differentiation-associated topoisomerase binding sites. We have therefore devised a novel strategy to purify covalently bound DNA/protein complexes (hence topoisomerase II bound DNA), using liquid-liquid phase partitioning. A significant increase in recovered protein-bound DNA was achieved in HL60 cells with the addition of $10^{-9}$ to $10^{-5}$M VP16-213 (etoposide) which is known to stabilise the DNA-Topoisomerase II complexes. Using the new technique, we obtained highly purified topoisomerase II-associated DNA from retinoic acid induced differentiating cells. Hybridisation of this DNA with DNA enriched for, or depleted of, protein-bound DNA from undifferentiated HL60 cells and cells induced to differentiate by phorbol ester or retinoic acid, demonstrated that the isolated DNA is enriched for specific sequences (since significant differences in hybridisation were obtained). This is, as far as we are aware, the first demonstration that topoisomerase II interacts at specific (or limited) sites in the genome during the induction of differentiation. Break-site DNA is currently being cloned for further evaluation of topoisomerase II's role in differentiation.

### Expression of *mdr1* and *gst-pi* in breast tumours, compared with the chemosensitivity of the same breast tumour cells *in vitro*

S. Stallard, R. Brown, N. Keith & S.B. Kaye

*CRC Dept of Medical Oncology, University of Glasgow, UK.*

Breast cell lines resistant to adriamycin have been shown to express elevated levels of *mdr1* and/or *gst-pi* mRNA. However, it is still to be demonstrated that these genes have a role in chemoresponsiveness of breast tumours *in vivo*. We have measured the mRNA levels of *mdr1* and *gst-pi* in more than 41 freshly excised human primary breast cancers by dot blot filter hybridisation, and in some cases Northern hybridisation. Relative levels of mRNA have been standardised by hybridisation of the same filter to a probe for a ubiquitously expressed gene, B2-microglobulin, and to poly d(T) for total mRNA levels. Detectable, if low, levels of *mdr1* expression were observed in 70% of the tumours, however in 30% there was considerably higher levels of expression (up to 100-fold higher). A range of *gst-pi* expression has also been observed in these tumours, but not with as large a fold variation. Chemosensitivity of cells grown in short-term culture from these tumours has been measured by an *in vitro* colony forming assay in the presence of adriamycin. The dose of adriamycin causing 50% inhibition of growth ($ID_{50}$) shows more than 100-fold variation between tumour samples. Correlations between $ID_{50}$ and gene expression levels did not reach significance; however, all the sensitive tumours *in vitro* had low *mdr1* expression levels. Similarly the resistant tumours tended to show high levels of *mdr1* expression. These data would support the hypothesis that the *mdr1* gene may play a role in determining the response of breast cancer cells, and in some tumours it may play a role in conjunction with other mechanisms of resistance.

### Are established small-cell lung cancer (SCLC) cell lines an appropriate model for clinical resistance?

R. Milroy, J.A. Plumb, S.W. Banham & S.B. Kaye

*CRC Dept of Medical Oncology, University of Glasgow and Dept of Respiratory Medicine, Royal Infirmary, Glasgow, UK.*

Many studies of drug resistance concentrate on cell lines made resistant by exposure to drug *in vitro* but the relevance of these lines to clinical resistance is not known. We have established a number of SCLC cell lines from biopsies from both untreated and treated patients and have demonstrated a 30-fold range in sensitivity to doxorubicin (DOX). In one case, an apparently resistant line, LS274 ($ID_{50}$ 220 nM), was established from an untreated patient. The patient showed a partial response to chemotherapy but later relapsed. A second cell line established from relapse tumour from the same patient was 4-fold more resistant to DOX (LS310, $ID_{50}$ 1050 nM) than LS274. In contrast, an apparently sensitive cell line (LCPH3, $ID_{50}$ 39 nM) was obtained from tumour taken from a patient at relapse. All the lines express L-dopa decarboxylase (DDC) and creatine kinase BB isoenzyme (CKBB) activities. The highest DDC activities (LS274 = 14, LS310 = 12 mIU $mg^{-1}$ protein) are observed in the two most resistant lines. Activities of CKBB do not relate to chemosensitivity *in vitro*. Furthermore, apparent resistance could not be explained by the growth characteristics of the cells since LS111 which grows as tight aggregates was of intermediate sensitivity ($ID_{50}$ 105 nM) while LS263 which grows as loose easily disaggregated clusters was more resistant ($ID_{50}$ 147 nM). The resistance modifier Verapamil (V) increased the drug sensitivity of a number of the cell lines by 2–3-fold but there was no relationship between either sensitivity *in vitro* or patient history and the activity of V. Thus, in one case chemosensitivities of two lines obtained from the same patient do reflect the patient history. However, the absolute chemosensitivity of an individual cell line does not reflect the clinical history of the tumour from which it was derived.

### Is there a role for resistance modifiers in cancer chemotherapy?

J.A. Plumb, R. Milroy & S.B. Kaye

*CRC Dept of Medical Oncology, University of Glasgow, Glasgow, UK.*

Activity of resistance modifiers is concentration dependent and for many, maximal activity is observed at a concentration of about 6.6 μM. Thus for the resistant small-cell lung cancer (SCLC) cell line H69LX10, derived from NCIH69 by chronic exposure to doxorubicin (DOX), drug sensitivity is increased 10-fold at 6.6 μM Verapamil (V) but only 3-fold at 2 μM (which is the maximum plasma concentration achievable clinically). We have shown that at similar concentrations the D-isomer of V (DV), thought to be less cardiotoxic than the L-isomer, is an effective resistance modifier in H69LX10. Further clinical studies will determine the role of DV based on achievable plasma levels. We have also determined the activity of a number of other resistance modifiers in H69LX10 which we have shown to express P-glycoprotein. Both Quinidine (Q, 6.6 μM) and Bepridil (B, 4 μM) increased the sensitivity to DOX, by 10 and 8-fold respectively, and these concentrations can be achieved in patients. All the modifiers stimulate the rate of lactate production by the cells and the increase correlates with the degree of resistance modification achieved with DOX. These observations are consistent with the theory that the modifiers act on the efflux pump protein, P170, possibly by inhibition of the energy supply. However, preliminary observations with intrinsically resistant non-SCLC cell lines indicate that V can increase DOX sensitivity to an extent which other modifiers, including DV, may not achieve; hence V may have more than one mechanism of action. Thus Q or B are better candidates for modification of the MDR phenotype, but V may still have a role through other undefined mechanisms.

### Resistance modification by cyclosporins in mouse multidrug-resistant (MDR) cell lines

P.R. Twentyman, K.A. Wright & J.G. Reeve

*MRC Clinical Oncology and Radiotherapeutics Unit, Hills Road, Cambridge CB2 2QH, UK.*

We have previously reported that the non-immunosuppressive cyclosporin, B3-243 (Sandoz), is a more potent resistance modifier (RM) than cyclosporin A (CsA) in the human small cell lung cancer MDR subline H69/LX4. These two compounds have now been examined for their RM properties in the EMT6 mouse tumour cell line and three MDR sublines (AR1.0, VR1.0, CR0.2) derived *in vitro* by exposure to adriamycin (ADM), vincristine (VCR) and colchicine (COL) respectively. Dose–response curves to cytotoxic drugs were obtained using a 3 day MTT assay in the presence or absence of the RM agent. Doses of RM used were 2.5 and 5.0 μg $ml^{-1}$ for CsA and 0.5, 1.0 and 2.5 μg $ml^{-1}$ for B3-243. Sensitisation ratios (SR = ratio of $ID_{50}$s in absence/presence of RM) are shown in the table for 2.5 μg $ml^{-1}$ of the RMs.

| | *Expression of* | | *SR (CsA/B3-243)* | | |
|---|---|---|---|---|---|
| *Cell line* | *P-170* | *Sorcin* | *ADM* | *VCR* | *COL* |
| EMT6 parent | ± | – | 9.4/9.2 | 4.6/2.4 | 6.6/7.8 |
| AR1.0 | ++ | ++ | 29.0/5.0 | 38.0/3.5 | 36.0/3.0 |
| VR1.0 | +++ | ± | 29.0/6.9 | 53.0/6.3 | 50.0/7.8 |
| CR0.2 | + | +++ | 22.2/9.6 | 34.0/20.0 | 26.0/16.2 |

It is clear that, at 2.5 μg $ml^{-1}$, CsA is generally a much more effective RM than B3-243 in sublines AR1.0 and VR1.0 where high level expression of P-170 is observed. However, the relative efficiency of B3-243 is much greater in CR0.2 (which has a lower level of MDR than AR1.0 and VR1.0).

### Measurement of individual cell glutathione content in human cancer biopsies using flow cytometry

D.W. Hedley, A.R. Hallahan & E.H. Tripp

*Ludwig Institute for Cancer Research, University of Sydney, NSW 2006, Australia.*

Glutathione (GSH) is the principal thiol-containing free radical scavenger in mammalian cells. Elevated levels may be an important cause of clinical resistance to agents which act through radical generation, and GSH depletion using buthionine sulphoximine (BSO) can improve the therapeutic index of alkylating agents used to treat human tumour xenografts. Rice *et al.*, (*Cancer Res.*, 1986, **46**, 6105) have described a flow cytometric (FCM) method in which the non-fluorescent probe monochlorobimane (MBCl) forms a fluorescent adduct with GSH under the action of GSH-S-transferase. We have confirmed that >99% of fluorescence is found in a low molecular weight cytoplasmic fraction of MBCl-stained EMT6 carcinosarcoma cells. By variably depleting these cells of GSH using BSO, and then performing simultaneous FCM and biochemical (Eyer & Podhradsky, *Anal. Biochem.*, 1986, **153**, 57) analyses we were able to show an excellent correlation between the two ($n=11$, $r=0.969$). Fluorescent calibration beads were run immediately prior to the FCM GSH samples, and were assigned a fluorescence value equivalent to a known cellular GSH content. Subsequent FCM measurements were quantitated by reference to this fluorescence standard. To date we have examined 4 non-Hodgkin's lymphomas and 8 carcinomas. Six samples were obtained by fine needle aspiration biopsy (FNAB) and 6 were surgical specimens. Mean GSH content was $0.24 \pm 0.12$ for the lymphomas and $0.99 \pm 0.39$ fmol cell$^{-1}$ for the carcinomas. The carcinomas showed a wide range in individual cell values, which was not simply related to cell size. Because it is rapid (<30 min), quantitative and can be performed using FNAB samples, FCM measurement of GSH content should be particularly useful for monitoring the effects of agents such as BSO which are intended to overcome GSH-mediated resistance.

### Mechanisms associated with differential cisplatin sensitivity expressed by three human ovarian carcinoma cell lines

S.A. Shellard, L.K. Hosking & B.T. Hill

*Cellular Chemotherapy Lab., Imperial Cancer Research Fund, Lincoln's Inn Fields, London WC2A 3PX, UK.*

Certain *in vitro* characteristics of two recently established human ovarian carcinoma cell lines (TR170 and TR175) (Hill *et al.*, *Int. J. Cancer*, 1987, **39**, 219) have been compared with those of the well established SK-OV-3 cell line of similar origin. Drug concentrations reducing cell survival by 50%, obtained from colony forming assay data, indicated a 10-fold and 26-fold hypersensitivity to cisplatin in the TR170 and TR175 cells respectively, when compared to the SK-OV-3 cell line. However, no significant differences in cellular uptake and DNA binding of drug were observed in these three cell lines. One possible explanation for the hypersensitivity of TR170 and TR175 cell lines to cisplatin may be attributed to their reduced levels of total glutathione content, glutathione reductase and glutathione peroxidase activities. In contrast a two-fold elevation in glutathione S-transferase activity (GST) was observed in the TR170 cells, whereas GST activities were comparable in the TR175 and SK-OV-3 cell lines. Preliminary data obtained from alkaline elution studies suggest that at equimolar concentrations of cisplatin less DNA damage is induced in the TR175 cells than in the more resistant SK-OV-3 cells. Furthermore both cell lines appear proficient in the repair of interstrand cross-links. In order to more clearly define the mechanisms of differential cisplatin sensitivities observed in these cell lines it is now proposed to quantitate the induction and removal of cisplatin adducts using polyclonal antisera, according to the procedure described earlier (Bedford *et al.*, *Cancer Res.*, 1988, **48**, 3019).

### Mitomycin C resistance: association with decreased NADPH cytochrome P-450 reductase activity in Chinese hamster ovary (CHO) cells *in vitro*

M.I. Walton, P.R. Hoban, C.N. Robson, P. Workman, A.L. Harris & I.D. Hickson

*MRC Clinical Oncology Unit, Cambridge CB2 2QH and Dept Clinical Oncology, Newcastle NE2 4HH and Oxford OX3 7LJ, UK.*

Mitomycin C (MMC) is the prototype bioreductive alkylating drug in clinical use. To investigate the mechanism of resistance, a MMC resistant cell line (MMC$^r$) was derived by exposing normal CHO (K1) cells to increasing drug concentrations in air. MMC$^r$ exhibited a 16-fold resistance to MMC but only a 2-fold resistance to the more readily activated MMC analogue BMY 25282. We examined the rates of MMC and BMY 25282 reduction in CHO-K1 and MMC$^r$ cells under aerobic (air) and anoxic ($N_2$) conditions. In addition we characterised some of the reductases present in these cells. MMC and BMY 25282 loss were monitored by reverse-phase HPLC. Reactions were carried out under $N_2$ using cell sonicates in the presence of NADH, NADPH and drug (50 $\mu$M). Rates of reduction of MMC and BMY 25282 were at least 30-fold greater under $N_2$ than air. Under $N_2$, MMC reduction was 2–3-fold lower in MMC$^r$ compared to K1 cells, e.g. $0.0782 \pm 0.018$ and $0.238 \pm 0.054$ nmol-min$^{-1}$ mg$^{-1}$ protein, respectively (mean $\pm 2$ s.e., $n=4$, $P<0.01$). Aerobic MMC reduction rates were too low to be assayed. By contrast rates of BMY 25282 reduction in MMC$^r$ and K1 cells were comparable in either air or $N_2$ and 10–20-fold faster than for MMC, e.g. ~3.0 nmol min$^{-1}$ mg$^{-1}$ protein in $N_2$. The activity of NADPH cytochrome P-450 reductase, an enzyme implicated in MMC bioactivation, was 3–4-fold lower in MMC$^r$ compared to K1 cells, e.g. $0.582 \pm 0.11$ versus $2.43 \pm 0.033$ nmol cytochrome c reduced min$^{-1}$ mg$^{-1}$ protein, respectively (mean $\pm 2$ s.e., $n=3$, $P<0.001$). No DT-diaphorase activity was detectable in MMC$^r$ cells. These results clearly implicate decreased P-450 reductase activity in the mechanism of increased resistance to MMC in MMC$^r$ CHO cells *in vitro*. Similar mechanisms may operate *in vivo*, and more readily activated derivatives might potentially overcome such resistance.

### Sensitivity of a methotrexate-resistant tumour cell line to a methotrexate-albumin-monoclonal antibody conjugate

K. Affleck & M.J. Embleton

*Cancer Research Campaign Laboratories, University of Nottingham, Nottingham NG7 2RD, UK.*

An osteogenic sarcoma cell line, 791T, was rendered resistant to methotrexate (MTX) by growth in increasing concentrations of MTX at consecutive passages, in the continuous presence of 12-*O*-tetradecanoylphorbol 13-acetate. A subline designated 791T-R120 was derived which required approximately 50 times more MTX than the parental line to achieve an $IC_{50}$ in a $^{75}$Se-selenomethionine incorporation cytotoxicity assay. Studies on the uptake of $^{3}$H-MTX suggested that resistance in this subline was predominantly due to diminished MTX transport.

A conjugate constructed by linking a monoclonal antibody against 791T (791T/36) to MTX via human serum albumin as a carrier was provided by Dr M.C. Garnett. This was tested for cytotoxicity against both parental and resistant cell lines in comparison with free MTX. 791T-R120 showed sensitivity to the conjugate equal to that of parental 791T, the respective $IC_{50}$s being 11 and 14 ng ml$^{-1}$ for the conjugate and 756 and 18 ng ml$^{-1}$ for MTX. It thus appeared that the altered (i.e. antibody-mediated) mechanism of uptake of MTX in conjugate form overcame the transport deficiency in the resistant cell line. it is suggested that drug–antibody conjugates might be useful in overcoming some clinical cases of drug resistance if administered in the appropriate form and by a suitable route.

**Enhancing the effectiveness of bioreductive drugs *in vivo***

J.C.M. Bremner, I.J. Stratford, L. Ding & G.E. Adams

*MRC Radiobiology Unit, Chilton, Oxon OX11 0RD, UK.*

Increasing tumour hypoxia to 100% can substantially enhance the effect of bioreductive drugs such as RSU1069 and SR4233. We have demonstrated this using a growth delay assay in KHT, RIF-1 and 16/C tumours. An *in vitro* cell survival assay of tumour cells treated *in vivo* 24 h previously, shows that when KHT tumours are treated with 80 mg $kg^{-1}$ RSU1069 followed 15 min later by clamping for 90 min, the surviving fraction is reduced to $5 \times 10^{-5}$. This is wholly consistent with the growth delay data. Other means of inducing hypoxia, such as using hydralazine, are less effective than clamping in enhancing the effect of bioreductive drugs both for growth delay and cell survival assays. A feature of tumours which could affect bioreductive drug therapy is the state of the tumour vascular bed. An aspect of this has been studied by implanting KHT and RIF-1 tumours into previously irradiated sites (15Gy). Tumour growth rate is reduced and there is evidence that the number of hypoxic cells increases. When treated with RSU1069 and clamping, the growth delay induced is substantial. However, when the slower growth rate of the untreated controls is considered, the overall response is no different from that of tumours in unirradiated sites.

*In vitro* studies show that an 80% reduction of (control) GSH, by BSO, produces a marked increase in the effect of RSU1069. However when tumour GSH levels are reduced to this level *in vivo* the enhancement of RSU1069 toxicity in clamped and unclamped tumours is only slight.

**Comparative effects of BW12C, hydralazine (HDZ) and nicotinamide (NCT) on relative perfusion of RIF-1 tumour and normal murine tissues**

D.J. Honess & N.M. Bleehen

*MRC Clinical Oncology and Radiotherapeutics Unit, Hills Road, Cambridge CB2 2QH, UK.*

The haemoglobin (Hb) modifier BW12C at 70 mg $kg^{-1}$ (Wellcome) and the vasodilator HDZ at 5 mg $kg^{-1}$ both increase tumour hypoxia; NCT at 1000 mg $kg^{-1}$ can radiosensitise some murine tumours. BW12C is thought to act primarily by inhibiting $O_2$ release from Hb; HDZ and NCT by reducing or increasing tumour blood flow respectively. All 3 drugs are potential modifiers of tumour response to chemotherapy. To improve our understanding of the systemic effects of the agents we have measured their effects on relative tissue perfusion (RTP) assayed by $^{86}$Rb uptake (10 $\mu$Ci per mouse 1 min before killing).

| | | *$^{86}$Rb extraction (RTP): % of control* | | |
|---|---|---|---|---|
| | | *BW12C (60 min)*[b] | *HDZ (15 min)*[b] | *NCT (60 min)*[b] |
| Leg tumour | 400 $mm^3$ | 64[a] | 26[a] | 101 |
| Leg tumour | 800 $mm^3$ | n.a. | 12[a] | n.a. |
| Flank tumour | 400 $mm^3$ | 34[a] | 9[a] | 104 |
| Leg muscle | | 90 | 107 | 66[a] |
| Kidney | | 127[a] | 64[a] | 150[a] |

[a]Value different from 100%, [b]$P<0.05$; time after injection.

Tumour RTP is unaffected by NCT and reduced by BW12C and HDZ, HDZ being more effective, and the changes are size and site dependent. Hence reduction in RTP may contribute to BW12C-induced tumour hypoxia. BW12C and NCT increase kidney perfusion while HDZ reduces it, suggesting that the half life of any renally cleared cytotoxic agent may be changed by these drugs. The variation between tissues indicates that tumour perfusion modifiers for combination with cytotoxic agents should be selected on the basis of their effects in normal tissues at risk.

**Biodistribution and molecular enzymology of SR 4233 reductive metabolism in mice**

M.I. Walton & P. Workman

*MRC Clinical Oncology Unit, Hills Rd, Cambridge CB2 2QH, UK.*

SR 4233 is the prototype of a novel class of benzotriazine di-*N*-oxide bioreductive agents which show high selective cytotoxicity towards hypoxic cells. This probably results from reductive bioactivation to toxic species. We have studied the pharmacokinetics of SR4233 in mouse plasma, tumour, liver and brain using an HPLC assay for SR4233 and its reduced metabolites SR 4317 (2e) and SR 4330 (4e). A dose of 0.2 mmol $kg^{-1}$ i.v. (35.6 $\mu g\,g^{-1}$) produced peak plasma concentrations of about 20 $\mu g\,ml^{-1}$. Elimination was biphasic with a $t_{1/2\alpha}<2$ min and a $t_{1/2\beta}$ of $26.5 \pm 1.4$ min (mean $\pm 2$ s.e.; $n=3$). The $AUC_{0-\infty}$ was 13.6 $\mu g\,ml^{-1}$ h. Peak plasma concentrations of SR4317 were between 6–8 $\mu g\,ml^{-1}$. The apparent $t_{1/2}$ was $43.3 \pm 12$ min and the $AUC_{0-\infty}$ was 12.1 $\mu g\,ml^{-1}$ h. Very similar results were produced after i.p. administration. SR 4233 tumour/plasma ratios in im KHT tumours were low at $34 \pm 12\%$ ($n=21$) compared to $174 \pm 42\%$ and $196 \pm 51\%$ for SR 4317 and SR 4330 respectively. Results for s.c. KHT, 16C, and RIF-1 tumours were similar but SR4233 ratios were significantly reduced in s.c. EMT6 tumours at $6.6 \pm 2.5\%$ ($P<0.01$). Brain/plasma ratios were similar to those in im KHT tumours. With time, SR 4233 liver/plasma ratios increased from 5–100% and SR 4330 ratios decreased from 1850–540%. Mouse liver microsomes readily reduced SR 4233 to SR 4317 under $N_2$ in the presence of NADH and particularly NADPH. SR 4233 reduction was rapid, e.g. 266 nmol $min^{-1}$ $mg^{-1}$ protein at 2 mM. SR 4233 was reduced predominantly by cyt P-450 with a more minor role for cyt P-450 reductase. Buttermilk xanthine oxidase catalysed this reaction but cytotosolic aldehyde oxidase did not. These results show that SR 4233 is readily bioactivated via reduction *in vivo* and *in vitro*, and are consistent with the expectation of extensive hypoxic tumour cell killing.

**The effects of bioreductive drugs on radiation sensitive cell lines *in vitro***

A. Keohane, J. Godden, M.A. Stephens, L. Ding, I. Stratford & G.E. Adams

*MRC Radiobiology Unit, Chilton, Didcot, Oxon, OX11 0RD, UK.*

We have used a group of cell lines (*irs*) selected for their sensitivity to ionising radiation and assessed the effects of Mitomycin C, RSU-1069 and SR-4233 under both aerobic and hypoxic conditions. The cells were cloned from V79 fibroblasts (Jones *et al.*, *Mutat. Res.*, 1987, **183**, 279) and exhibit a range of sensitivity to ionising radiation in air. Treatment of cells with RSU-1069 and SR-4233 resulted in selective toxicity towards hypoxic cells. Hypoxic toxicities to the *irs* cells were similar to those of V79's indicating that the radiation sensitive cells have not lost their capacity to reduce the drugs under anaerobic conditions. In air the mutant cells are 10 × more sensitive than V79's which may be related to the hypersensitivity of these cells to radiation in air.

Levels of intracellular GSH can profoundly alter sensitivity to drugs and radiation. The concentration of GSH in the *irs* cells is 3 × lower than in V79. However, if this is important it would also be expected to affect sensitivity to the bioreductive drugs under $N_2$ and is clearly not the case.

To further characterise these cell lines we have measured radiosensitivity in $N_2$ and radiosensitisation by misonidazole. The OER is 3 and 1.7 in wild type and *irs1* cells respectively. 1mM misonidazole gives an ER of 1.7 in both cell lines. Similar experiments are being carried out with RSU 1069 to determine if the *irs* cells are also hypersensitive to the radiosensitising action of this drug. (Supported by NCI grant No. R01 CA44 126-01).

### Comparison of uptake, free radical generation and cytotoxicity of C1941 with doxorubicin in MCF-7 human breast cancer cells

G.R. Fisher & L.H. Patterson

*Dept of Pharmacy, Leicester Polytechnic, Leicester LE1 9BH, UK.*

CI941 is an anthrapyrazole DNA complexing agent broadly based on doxorubicin (DOX) and is currently undergoing phase 1 clinical trials. The NAD(P)H dependent metabolic reduction of DOX to an anthraquinone free radical intermediate which concomitantly generates reactive oxygen species from dioxygen (termed redox cycling) has long been implicated in the cytotoxicity of this agent. This study compares redox cycling by CI941 with doxorubicin in MCF-7 cells and its relationship to cytotoxicity. Drug induced redox cycling in MCF-7 S9 cell fraction was monitored by X-band ESR spectroscopy, NADPH oxidation at 340 nm and superoxide anion ($O_2^-$) generation as Superoxide Dismutase inhibitable reduction of acetylated cytochrome c. Cytotoxicity was measured after 24 h drug treatment of $10^5$ exponentially growing cells and a subsequent 96 h drug free incubation. To measure drug uptake, MCF-7 cells were incubated with drug (15 $\mu$M) for between 1–90 min, the cells centrifuged and the remaining supernatant analysed by HPLC. The results show that the basal rate NADPH oxidation ($72 \pm 18$ pmol min$^{-1}$ mg protein$^{-1}$) was stimulated by DOX (11.5-fold) but was unaffected by CI 941. Basal rate $O_2^-$ formation was undetectable in MCF-7 S9 but in the presence of DOX (100 $\mu$M) was 7.5 nmol min$^{-1}$ mg protein$^{-1}$ while CI941 showed no effect on $O_2^-$ formation. Doxorubicin produced a broad singlet ESR spectrum in both MCF-7 S9 fraction and intact cells under anaerobic conditions. However, CI941 did not generate an ESR signal under such conditions. The cytotoxic concentration of CI941 ($ED_{50} = 1$ nM) was considerably less than DOX (>40 nM) while cell uptake after 90 min for both drugs was between 45–50%. The results indicate that CI941 does not redox cycle in MCF-7 cells although it is more cytotoxic than doxorubicin in this cell line. It appears that although free radical generation may be important in the mechanism of cytotoxicity of DOX it is not related to the MCF-7 cell-kill activity of CI941.

### *In vivo* uptake of $^{131}$I-5-iodo-2-deoxyuridine by malignant tumours in man

P.A. Philip, K.D. Bagshawe, F. Searle, R.H.J. Begent, A. Green, E.S. Newlands & G.J.S. Rustin

*Cancer Research Campaign Laboratories, Charing Cross Hospital, London W6 8RF, UK.*

5-Iodo-2-deoxyuridine (IUdR) is a synthetic analogue of thymidine which is taken up by cells in the S-phase of the cell cycle, being incorporated into newly synthesised DNA. Labelled DNA (with IUdR) confers upon the cell reduced viability and increased sensitivity to ionising irradiation and UV light. IUdR uptake by normal cells results in many side effects precluding its effective use in therapy. It has been shown recently that selective uptake can be significantly enhanced when mice bearing xenografts were pretreated with hydroxyurea (HU) and antemetabolites. The former arrests DNA synthesis in the normal cells (and sensitive tumour cells) and the latter inhibits *de novo* thymidine synthesis (Bagshawe *et al.*, *Br. J. Cancer*, 1987, **55**, 299).

26 patients with various malignancies, judged to be resistant to conventional therapy, were investigated. HU 2.0 g twice weekly p.o. was given for 2–3 weeks to enhance resistance of tumour cells. 5-Fluorouracil 200 mg m$^{-2}$ was given i.v. at time 0 min, followed by 600 mg m$^{-2}$ at time 30 min. HU 3.0 g m$^{-2}$ was given i.v. at time 35 min. 10–15 min later $^{131}$I labelled IUdR (5–10 mCi) was given i.v. over 10 min. Planar imaging with a gamma-camera was performed approximately 24 and 48 hours from administration of the radiolabelled IUdR. Thyroid blockade was with KI 120 mg tds for 7 days and $KClO_3$ was given 200 mg tds (4 days) to reduce secretion of iodide into the stomach.

13 (50%) patients showed positive uptake in at least one disease site. Of the 43 known active disease sites only 15 (34.9%) exhibited uptake of radioactivity. Three out of 4 brain tumours showed significant uptake. Other sites of uptake include liver, lung and pelvis, and a subcutaneous nodule. No false positive images were encountered.

### Enhancement of 1-$\beta$-D-arabinofuranosylcytosine (ARA-C) metabolism and toxicity by pre-treatment with gallium

D.W. Hedley & E.H. Tripp

*Ludwig Institute for Cancer Research, University of Sydney, NSW 2006, Australia.*

Gallium nitrate (NSC15200) has significant activity against refractory lymphomas in man, while sparing normal proliferating tissues. We recently identified ribonucleotide reductase as the probable main enzyme target (Hedley *et al.*, *Cancer Res.*, 1988, **48**, 3014). It has been previously shown that inhibitors of this enzyme can enhance the metabolism and toxicity of ARA-C, and we therefore examined the interaction of gallium and ARA-C in CCRF-CEM human T-lymphoblasts. Pre-treatment for 24 h with a cytostatic (480 $\mu$m) concentration of gallium increased the percentage of cells in S-phase from 44 to 66%, as determined by DNA flow cytometry. There was a fall in the size of the dCTP pool from 27 to 19 pmol $10^{-6}$ cells, and the activity of the salvage enzyme deoxycytidine kinase (which is feedback inhibited by dCTP) increased from 29.8 to 62.4 pmol h$^{-1}$ $10^{-6}$ cells. Uptake of 5 $\mu$M $^3$H-labelled ARA-C was measured using the method of Plagemann *et al.* (*Cancer Res.*, 1978, **38**, 978). Pre-treatment with gallium approximately doubled the rate of isotope incorporation. Separation of $^3$H-ARA-C metabolites using thin layer chromatography showed that 86% was present as ARA-CTP with gallium pre-treatment, compared to 81% for controls. Finally, coincubation of cells with cytostatic concentrations of gallium and ARA-C showed that the combination was more toxic than either agent alone. These results are further evidence that gallium acts primarily as a ribonucleotide reductase inhibitor, and suggest that a gallium/ARA-C combination be tested in patients with refractory lymphomas.

**Cisplatin induced mutation frequencies in human tumour cell lines**

C.N. Parris & J.R.W. Masters

*Institute of Urology, University College London, St Paul's Hospital, London WC2H 9AE, UK.*

Drug resistance rarely develops in testicular tumours (TGCT) and 80–90% of metastatic TCGT are cured. However chemotherapy is mutagenic and can induce drug resistance (Kerbel & Davies, *Lancet*, 1982, **ii**, 977). Therefore one explanation for the curability of TGCT is that they are less mutable than other solid tumours, such as bladder cancer. We compared spontaneous and cisplatin-induced mutation frequencies (MF) at the hypoxanthine guanine phosphoribosyl transferase (HGPRT) locus in two bladder (RT4, RT112) and two testicular (833K, SuSa) cancer cell lines. Following a 1 h exposure to an equimolar concentration of cisplatin ($4\,\mu g\,ml^{-1}$) and concentrations reducing survival by 50% in all cell lines ($IC_{50}$), MF were compared (see table).

| | | *Mutation frequency × $10^{-6}$ surviving cells* | |
|---|---|---|---|
| *Cell type* | *Spontaneous MF* | *Induced MF @ $4\,\mu g\,ml^{-1}$* | *induced MF @ $IC_{50}$* |
| RT112 | 11.5±5.5 | 55±35 | 55±35 |
| RT4 | 4.3±0.3 | 4.3±0.2 | 5.2±1.4 |
| 833K | 3.8±0.5 | 13.2±1.5 | 6.0±1.2 |
| SuSa | 4.6±0.3 | 9.5±1.5 | 4.5±0.3 |
| | $P$=0.462 | $P$=0.546 | $P$=0.462 |

These data indicate that MF are similar in testicular and bladder cancer cells, and do not account for differential sensitivity between these tumour types. However, following the same dose of cisplatin, fewer testicular tumour cells survive so fewer mutants develop, perhaps indicating why testicular tumours rarely develop resistance.

**A bispecific monoclonal antibody against methotrexate and a human tumour associated antigen: antibody production purification and characterisation**

M.V. Pimm, R.A. Robins, E. Jacobs, A.J. Markham, A.D. Curran & R.W. Baldwin

*Cancer Research Campaign Laboratories, University of Nottingham, NG7 2RD, UK.*

The development of monoclonal antibodies reactive with human tumour-associated antigens has led to interest in their use as targeting agents to deliver therapeutic agents to tumour sites. One approach is to produce hybrid bispecific antibodies, one combining site of which reacts with tumour associated target antigen and the other with the therapeutic agent. In the present study a bispecific monoclonal antibody, reactive with methotrexate (MTX) and a tumour associated gp72 antigen has been produced by fusing spleen cells from MTX-HSA immunised mice with the existing 791T/36 hybridoma.

The hybrid antibody had gamma-1 and gamma-2b heavy chains from the parent anti-MTX splenocyte and 791T/36 hybridoma respectively. It was purified from anti-MTX and anti-gp72 antibodies in the hybridoma culture supernatant by combinations of affinity chromatography on MTX-agarose and step-wise acid elution from Sepharose-protein A. In competitive ELISA assays, binding to MTX-HSA was inhibited by excess MTX-HSA but not by HSA. Free MTX and aminopterin inhibited binding, but much less efficiently than the equivalent amount of MTX as MTX-HSA conjugate. In reaction against tumour cells, hybrid antibody, but not the anti-MTX, reacted with gp72 positive 791T cells and not with antigen negative (Colo-205) cells as detected by subsequent reaction with fluorescein labelled anti-mouse IgG. Simultaneous dual binding between tumour cell surface antigen and MTX was demonstrated by the ability of hybrid antibody to bridge between 791T tumour cells and MTX as MTX-HSA conjugate, reaction here being detected with fluorescein labelled anti-HSA antiserum.

These studies indicate the potential of this bispecific antibody for improving therapeutic efficacy of MTX. The reaction with conjugated rather than free MTX could be an advantage since targeting of conjugates would be expected to increase many fold the number of molecules of drug carried by or localizing in pre-targeted antibody. The effect of the hybrid antibody on the cytotoxicity of MTX and MTX-HSA for gp72 positive tumour cells is now being investigated (Embleton *et al.*, next abstract).

**Selective cytotoxicity against tumour cell lines mediated by a bispecific monoclonal antibody and a methotrexate albumin conjugate**

M.J. Embleton, M.V. Pimm, R.A. Robins, A.J. Markham, A. Charleston & E. Jacobs

*Cancer Research Campaign Laboratories, University of Nottingham, Nottingham NG7 2RD, UK.*

A bispecific monoclonal antibody reactive with methotrexate (MTX) and a tumour-associated gp72 antigen was tested for ability to modify the cytotoxic effects of MTX and MTX-human serum albumin conjugate (MTX-HSA) against gp72-positive and gp72-negative tumour cell lines using a $^{75}$Se-selenomethionine incorporation cytotoxicity assay. In some tests the drug or conjugate was titrated against a fixed concentration of antibody, and in others the antibody was titrated against a single concentration of the cytotoxic agent.

The bispecific antibody did not influence the cytotoxic effect of free MTX at high or low doses on either antigenic or non-antigenic cell lines. However, it enhanced the cytotoxicity of MTX-HSA (normally much lower than MTX) against gp72-positive cell lines. At low antibody and high MTX-HSA concentrations augmentation was weak, but antibody at saturating levels produced significant cytotoxicity using MTX-HSA at a concentration too low to be cytotoxic in its own right. Monoclonal antibodies against MTX or gp72 alone had no consistent effect, and bispecific antibody was inactive in the absence of MTX-HSA. The combination of bispecific antibody and MTX-HSA appeared to result in capture and delivery of an optimum number of MTX molecules, consistent with the greater affinity of antibody with conjugated rather than free drug and the large number of drug residues per molecule of conjugate. Targeting of relatively non-toxic conjugates by pre-localised bispecific antibody might be a valid approach for anti-tumour therapy.

**Tumour localisation of 791T/36 Fab/c fragment compared to whole antibody**

S. Demignot, M.C. Garnett & R.W. Baldwin

*Cancer Research Campaign Laboratories, University of Nottingham, Nottingham NG7 2RD, UK.*

Fab/c, a monovalent antibody fragment containing Fab and Fc regions, was shown to have a similar half life to whole antibody (*BACR*, Dec. 1988), but it showed a faster equilibration and greater extravasation. The maximum binding of the Fab/c fragment was 10–30% compared to 70% for whole antibody as measured by assay of immunoreactivity at infinite antigen excess. We now report an experiment to determine the tumour localisation of this fragment in nude mice bearing 791T (antigen positive) and Colo 205 (antigen negative) xenografts. At 72 hours the tumour to blood ratio for antibody was 1.7 and for Fab/c fragment 1.2 in the 791T xenograft compared to 0.6 in the Colo 205 xenograft. Expressed as % injected dose per g tumour, both antibody and Fab/c showed a similar uptake with a maximum at about 5–6%. In tumour bearing mice the serum half life of the whole antibody was slightly less than the Fab/c fragment and there was some uptake of whole antibody, but not Fab/c, to liver and spleen (antibody tissue to blood ratios of 0.5 and 1.5 respectively). These differences between antibody and Fab/c distribution may be due to a small amount of circulating antigen causing some antibody but not Fab/c uptake to liver and spleen. Despite its lower affinity Fab/c shows good tumour localisation. The lower molecular weight and monovalency of this fragment may be an advantage for antibody targeting by permitting better tumour penetration than whole antibody.

**The effect of colorectal carcinoma on T-lymphocytes circulating through the tumour**

K.M. Rigg, B.K. Shenton, R.M.R. Taylor & T.W.J. Lennard

*University Department of Surgery, The Medical School, Framlington Place, Newcastle upon Tyne NE2 4HH, UK.*

Malignancy is known to depress the host immune response. To determine if passage of lymphocytes through the tumour affected their number and function, 11 patients undergoing curative resection for colorectal cancer were studied. At operation blood was taken from the artery supplying and vein draining the tumour. Separated lymphocytes from both artery and vein were stimulated with the T-cell mitogen, phytohaemagglutanin (PHA) for 72 hours, after which lymphocyte transformation (LT) was measured by uptake of tritiated thymidine. Using monoclonal antibodies and flow cytometry, the percentages of CD3(T), CD8(T suppressor/cytotoxic) and CD4(T helper) cells in the total circulating lymphocyte population, along with basal and stimulated interleukin-2 receptor (Il-2R) were enumerated. The results are expressed as median (range).

| | *LT* (*c.p.m.* $\times 10^3$) | *Basal Il-2R* (%) | *Stim. Il-2R* (%) |
|---|---|---|---|
| Arterial | 19 (7–52) | 5 (4–7) | 73 (39–91) |
| Venous | 12 (2–48)[a] | 5 (3–9) | 70 (28–88) |

[a]$P<0.04$ (paired $t$ test).

There was a significant decrease in lymphocyte transformation following passage of lymphocytes through the tumour, but there was no significant difference in T lymphocyte subpopulations or Il-2R expression between arterial and venous blood. Colorectal cancer may down regulate function of T lymphocytes circulating through the tumour.

**The role of oligosaccharide side-chains in the blood clearance of antibody–toxin conjugates**

A.J. Cumber, G.D. Parnell, R.V. Henry, J.A. Forrester & E.J. Wawrzynczak

*Institute of Cancer Research, Sutton, Surrey SM2 5NG, UK.*

The rate of blood clearance of a panel of antibody–toxin conjugates made by linking different ribosome-inactivating proteins to the monoclonal antibody Fib75 was monitored after intravenous administration to rats. Fib75-ricin A was rapidly lost from the circulation because of the preferential uptake of a proportion of molecules bearing oligomannose side-chains by the liver. In contrast, a Fib75 conjugate made with the purified Al-chain of ricin, which contains a single complex-type oligosaccharide side-chain, was not rapidly cleared and persisted in the bloodstream for a prolonged period of time (A.J. Cumber *et al.*, *Biochem. Soc. Trans.*, 1989, **17**, 137).

The clearance rates of Fib75 conjugates made with ricin A1-chain, gelonin and momordin, each containing a single oligosaccharide side-chain, were similar. However, these conjugates disappeared from the circulation more rapidly than a Fib75 conjugate made with abrin A-chain which is not glycosylated. A Fib75 conjugate made with gelonin which had been chemically treated to destroy carbohydrate residues persisted in the bloodstream longer than Fib75-gelonin suggesting that recognition of oligosaccharide side-chains of a type other than the oligomannose type may accelerate clearance.

**Human small cell lung cancer antigen recognised by monoclonal antibody SWA11 mediates immunotoxin cytotoxicity**

E.J. Wawrzynczak, R.V. Henry, G.D. Parnell & E.J. Derbyshire

*Institute of Cancer Research, Sutton, Surrey SM2 5NG, UK.*

The potential of mouse monoclonal antibodies raised against human small cell lung cancer (SCLC) to form cytotoxic conjugates with ricin A-chain was tested using an indirect assay. The classic SCLC line HC12 was first treated with a saturating concentration of anti-SCLC monoclonal antibody, washed to remove unbound antibody and then incubated with goat anti-mouse immunoglobulin (GAMIg) Fab′ fragment linked by a disulphide bond to a single molecule of ricin A-chain. At a non-toxic concentration of GAMIg Fab′-ricin A, protein synthesis by HC12 cells pretreated with the SWA11 monoclonal antibody (Smith *et al.*, *Lung Cancer*, 1988, **4**, suppl., A12; Smith *et al.*, *Br. J. Cancer*, 1989, **59**, in the press) was decreased to less than 20% of that in untreated cell cultures. In contrast, there was no significant inhibition of protein synthesis in cells exposed to SWA11 alone or in cells pretreated with isotype-matched antibodies (binding to antigens other than that recognised by SWA11) and then incubated with GAMIg Fab′-ricin A. Our experiments indicate that the SCLC antigen recognised by SWA11 mediates the internalisation of ricin A-chain via a route leading to cell intoxication and is a suitable target for the development of ricin A-chain immunotoxins with selective cytotoxicity to human SCLC.

### Theoretical studies on combined mIBG/TBI therapy of neuroblastoma micrometastases

J.A. O'Donoghue, T.E. Wheldon, A. Barrett, J.W. Babich, J. Moyes & S. Meller

*Beatson Oncology Centre, Belvidere Hospital, Glasgow G31 4PG; and The Royal Marsden Hospital, Sutton, Surrey SM2 5PT, UK.*

Measurements of $m^{125}IBG$ uptake in surgically excised samples of neuroblastoma from 6 patients were used to assess absorbed dose to micrometastases. Doses to achieve a 50% probability of cure were calculated using a simple analysis. Two categories were examined: (a) absorbed dose greater than 50% cure dose; (b) absorbed dose less than 50% cure dose. The number of patients falling into each category depends on the tumour size and on the radionuclide half-life in the tumour. For a 1 mm diameter tumour the number of category (a) patients increases from 0 ($t_{1/2}=1.2$ days) to 4 ($t_{1/2}=8$ days). MIBG is less effective for smaller tumours due to the reduced absorption of the beta-energy from $^{131}I$. For a 0.2 mm diameter tumour the number of category (a) patients increases from 0 ($t_{1/2}=1.2$ days) to 2 ($t_{1/2}=8$ days). The use of mIBG in combination with total body irradiation (TBI) and autologous bone marrow rescue was investigated. For a 1 mm tumour the number in category (a) increases from 3 ($t_{1/2}=1.2$ days) to 5 ($t_{1/2}=8$ days). For a 0.2 mm tumour all 6 patients remain in category (a) for the full range of tumour half-times considered. Tumour doses with combined mIBG/TBI are significantly higher than achievable with either modality alone. Additionally, the two modalities complement each other as TBI is relatively more effective at eliminating small (<1 mm) micrometastases and single cells whereas in mIBG is better for larger (≥1 mm) micrometastases.

### Radioimmunoimaging and CT in malignant melanoma

A.T. Elliott, R.M. MacKie, T. Murray, V.R. Doherty, G. Gillen & F.G. Adams

*Depts of Clinical Physics, Dermatology and Radiology, Western Infirmary, Glasgow G11 6NT, UK.*

A monoclonal antibody raised against the high molecular weight melanoma antigen was labelled with indium-111 and injected intravenously into 25 patients with malignant melanoma. The results obtained from images at 24 and 96 hours post i.v. administration of the antibody were compared with results obtained from computerised tomography studies with regard to detection of previously unrecognised sites of metastatic disease and apparent false positive localisation. Detailed study of the patients' clinical condition and detection rates using the two methods suggests that both methods detect approximately 80% of clinically and pathologically confirmed metastases. Of 62 known metastases, the antibody detected 60 (81%), with 17 false positive results. False negatives were most common in the lung. In 8 patients the two methods were considered of equal value, in 10 the monoclonal gave a greater amount of clinically relevant information, and in 7 the CT was superior. In three patients clinically significant metastatic lesions were detected by the radiolabelled monoclonal which had not been previously recognised either by CT scanning or on clinical grounds.

No patients had any adverse reaction to the antibody and in the course of our study the dose of antibody was reduced from 20 mg to 200 mg with no apparent loss of sensitivity. In at least 2 patients uptake of the labelled monoclonal into tumour sites would have been adequate for effective targeted radiotherapy.